# Briarane Diterpenoids from the Gorgonian *Dichotella gemmacea*

**DOI:** 10.3390/md12126178

**Published:** 2014-12-18

**Authors:** Ming-Ping La, Jiao Li, Cui Li, Hua Tang, Bao-Shu Liu, Peng Sun, Chun-Lin Zhuang, Tie-Jun Li, Wen Zhang

**Affiliations:** Research Center for Marine Drugs, and Department of Pharmacology, School of Pharmacy, Second Military Medical University, 325 Guo-He Road, Shanghai 200433, China; E-Mails: lmp12@163.com (M.-P.L.); lijiao_2012@126.com (J.L.); licuiwan@163.com (C.L.); tanghua0309@126.com (H.T.); liubaoshu@126.com (B.-S.L.); sunpeng78@126.com (P.S.); zclnathan@163.com (C.-L.Z.)

**Keywords:** structure elucidation, briarane diterpenoids, tumor cell growth inhibitory activity, gorgonian, *Dichotella gemmacea*

## Abstract

Seven new briarane diterpenoids, gemmacolides AS-AY (**1**–**7**), were isolated together with ten known analogues (**8**–**17**) from the South China Sea gorgonian *Dichotella gemmacea*. The structures of the new compounds were elucidated by the detailed analysis of spectroscopic data and comparison with reported data. The absolute configuration of compounds was determined based on electronic circular dichroism (ECD) experiments and genetic correlations as well. Compounds **15** and **16** were reported for the first time for the gorgonian. In the preliminary *in vitro* bioassays, compound **5** showed potential growth inhibitory activity against MG63 cells.

## 1. Introduction

Gorgonian corals of the family *Ellisellidae* are proven to be rich source of briarane diterpenoids that are well known for displaying a wide spectrum of bioactivities, including cytotoxic, anti-inflammatory, antiviral, antifouling, insecticidal and immunomodulatory effects [1,2,3,4]. In the course of searching for novel and bioactive secondary metabolites from marine organisms of the South China Sea [5,6], the tumor cell growth inhibitory activity of briarane diterpenoids attracted our attention, leading to the isolation of a series of briarane-type diterpenes the gorgonians *Dichotella* *gemmacea* and *Junceella* *gemmacea* [7,8,9,10,11].

**Chart 1:**
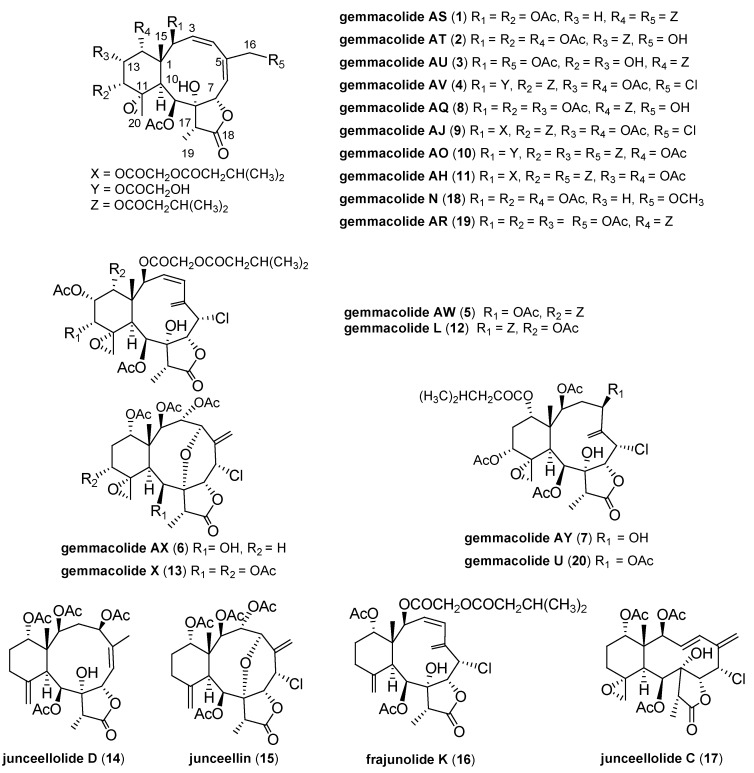
Structures for compounds **1**–**20**.

In particular, gemmacolides J, V and Y showed potential activity against A549 cells, being more active than the positive control adriamycin [9,10]. These results encourage subsequent studies of this class of metabolites, leading to the new collection of gorgonian *D. gemmacea*, a promising source of briarane diterpenoids. Chemical investigation on the acetone extract of these animals resulted in the isolation and structure elucidation of seven new briaranes, namely gemmacolides AS–AY (**1**–**7**), together with ten known analogues, gemmacolide L (**12**), gemmacolide X (**13**), gemmacolide AH (**11**), gemmacolide AQ (**8**), gemmacolide AJ (**9**), gemmacolide AO (**10**), Junceellolide C (**17**), junceellolide D (**14**), junceellin (**15**), and frajunolide K (**16**) [8,9,10,12,13,14] (Chart 1). The structures of these compounds were elucidated by detailed analysis of spectroscopic data and comparison with the reported analytical data for the known compounds. The isolates were tested *in vitro* for their tumor cell growth inhibitory activity. We report here the isolation, structure elucidation, and biological activity of these new compounds.

## 2. Results and Discussion

Freshly collected specimens of *Dichotella* *gemmacea* were immediately frozen to −20 °C and stored at this temperature before extraction. The usual workup for the extraction and isolation of briarane diterpenoids [7,8,9,10,11] yielded seventeen pure compounds (**1**–**17**).

Gemmacolide AS (**1**) was isolated as a white amorphous powder. Its molecular formula was established as C_36_H_50_O_14_ by HRESI-MS. The IR spectrum showed absorption bands of hydroxy (3478 cm^−1^), γ-lactone (1778 cm^−1^), and ester (1739 cm^−1^) functionalities. This observation was in agreement with the signals in the ^13^C NMR and DEPT spectra (Table 1) for ten *sp^2^* carbon atoms (6 × OC=O, CH=CH, CH=C) at lower field and twenty three *sp*^3^ carbon atoms at higher field (1 × C, 4 × CH, 3 × CH_2_, 9 × CH_3_, 2 × OC, 5 × OCH, 2 × OCH_2_), accounting for eight double bond equivalents. The remaining double bond equivalents were due to the presence of four rings in the molecule.

**Table 1:** ^13^C NMR data of gemmacolides AS–AY (**1**–**7**) ^a^.

No.	1	2	3	4	5	6	7
1	47.2, C	46.5, C	46.4, C	46.6, C	47.0, C	47.1, C	48.8, C
2	74.5, CH	75.6, CH	74.3, CH	75.3, CH	71.9, CH	72.8, CH	70.4, CH
3	132.5, CH	131.2, CH	128.6, CH	131.2, CH	129.2, CH	65.3, CH	42.8, CH_2_
4	127.6, CH	129.6, CH	127.8, CH	129.1, CH	129.1, CH	78.9, CH	70.5, CH
5	140.0, C	144.5, C	139.3, C	139.8, C	136.8, C	135.0, C	140.0, C
6	122.4, CH	123.8, CH	122.8, CH	126.3, CH	64.3, CH	54.6, CH	52.0., CH
7	78.9, CH	78.7, CH	78.8, CH	78.4, CH	78.7, CH	80.2, CH	81.3, CH
8	81.2, C	81.1, C	81.2, C	81.0, C	80.0, C	83.8, C	81.3, C
9	64.1, CH	63.9, CH	64.1, CH	63.7, CH	75.7, CH	71.7, CH	70.8, CH
10	32.8, CH	32.7, CH	31.3, CH	32.7, CH	32.7, CH	41.2, CH	35.7, CH
11	59.3, C	58.4, C	60.1, C	58.2, C	57.0, C	56.9, C	57.2, C
12	73.0, CH	73.2, CH	76.0, CH	72.6, CH	73.3, CH	-	72.8, CH
13	29.0, CH_2_	66.3, CH	66.3, CH	66.3, CH_2_	66.6, CH	24.8, CH	29.5, CH
14	73.1, CH	73.9, CH	77.5, CH	73.7, CH	72.5, CH	74.0, CH	72.6, CH
15	14.2, CH_3_	14.4, CH_3_	14.6, CH_3_	14.3, CH_3_	14.7, CH_3_	15.8, CH_3_	14.0, CH_3_
16	62.8, CH_2_	63.8, CH_2_	63.1, CH_2_	44.4, CH_2_	117.1, CH_2_	118.8, CH_2_	118.4, CH_2_
17	44.2, CH	44.1, CH	44.2, CH	44.0, CH	48.5, CH	49.6, CH	51.3, CH
18	175.4, C	175.2, C	175.3, C	174.9, C	175.6, C	175.0, C	175.8, C
19	6.4, CH_3_	6.3, CH_3_	6.4, CH_3_	6.3, CH_3_	8.6, CH_3_	7.2, CH_3_	5.9, CH_3_
20	49.1, CH_2_	49.0, CH_2_	49.1, CH_2_	49.1, CH_2_	49.7, CH_2_	51.9, CH_2_	50.7, CH_2_
9-OAc	170.2, C	170.2, C	170.2, C	170.1, C	169.7, C	No.	169.3, C
	21.6, CH_3_	21.6, CH_3_	21.6, CH_3_	21.5, CH_3_	21.0, CH_3_		21.3, CH_3_
R_1_	169.1, C	170.9, C	169.3, C	171.8, C	see 1′–6′	170.3, C	172.9, C
	21.3, CH_3_	21.5, CH_3_	21.3, CH_3_	61.1, CH_2_		20.6, CH_3_	21.6, CH_3_
R_2_	170.1, C	169.7, C	No.	see 1′–5′	169.5, C	169.7, C	No.
	21.2, CH_3_	20.9, CH_3_			20.8, CH_3_	20.4, CH_3_	
R_3_	No.	see 1′–5′	No.	169.7, C	170.5, C	No.	169.5, C
				20.5, CH_3_	20.6, CH_3_		21.3, CH_3_
R_4_	see 1′–5′	170.1, C	see 1′–5′	170.6, C	see 1ʺ–5ʺ	No.	see 1′–5′
		20.8, CH_3_		21.0, CH_3_			
R_5_	see 1ʺ–5ʺ		170.2, C			170.1, C	
			20.9, CH_3_			21.0, CH_3_	
1′	172.0, C	171.8, C	173.7, C	171.4, C	166.1, C		172.9, C
2′	43.3, CH_2_	42.6, CH_2_	43.1, CH_2_	43.5, CH_2_	60.9, CH_2_		43.2, CH_2_
3′	25.7, CH	25.0, CH	25.2, CH	25.7, CH	172.4, C		24.7, CH
4′	22.4, CH_3_	22.4, CH_3_	22.6, CH_3_	22.4, CH_3_	42.8, CH_2_		22.7, CH_3_
5′	22.3, CH_3_	22.3, CH_3_	22.3, CH_3_	22.3, CH_3_	25.7, CH		22.5, CH_3_
6′					22.3, 2 × CH_3_		
1ʺ	172.3, C				172.6, C		
2ʺ	42.9, CH				43.3, CH_2_		
3ʺ	24.8, CH_3_				25.1, CH		
4ʺ and 5ʺ	22.5, 2 × CH_3_				22.4, 2 × CH_3_		

^a^ 100 MHz, in CDCl_3_, assignments made by CHEPT, HSQC, and HMBC.

**Figure 1:**
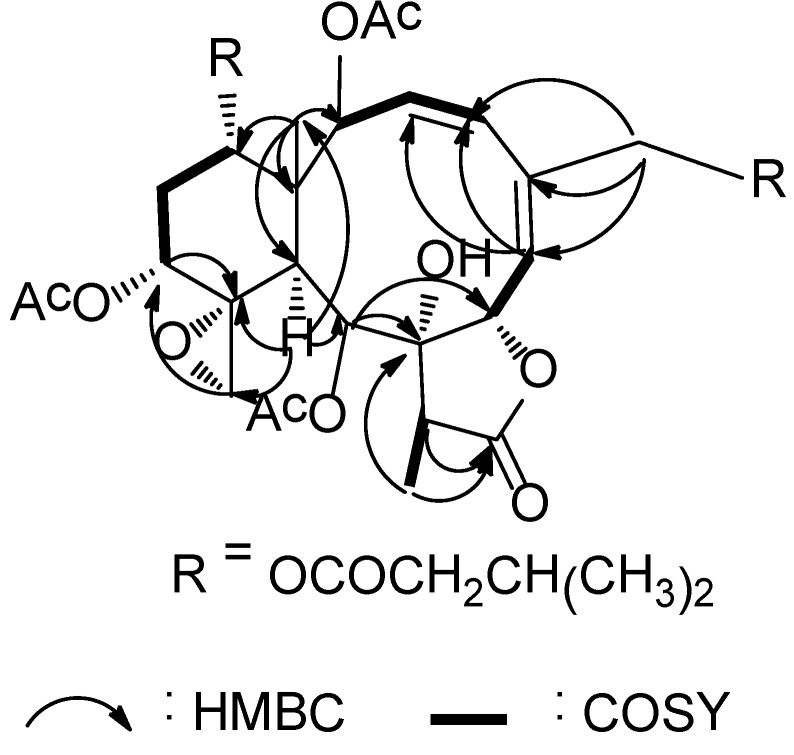
Key HMBC (arrow H→C) and COSY (bond) spin coupling systems for compound **1**.

**Figure 2:**
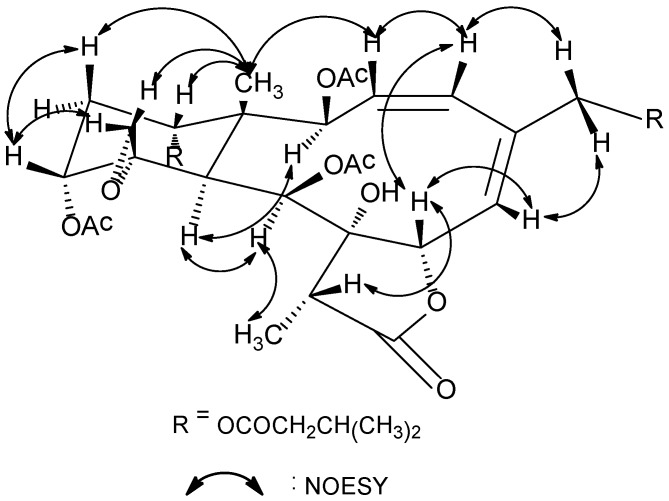
Key NOESY correlations for compound **1**.

^1^H and ^13^C NMR spectra of **1** showed great similarity to those of gemmacolide N (**18**) [11], the difference was the acetyl group at C-14 and the methoxy group at C-16 in gemmacolide N (**18**) being replaced by two isovaleric acetyl in **1** due to the obvious HMBC correlations from the secondary alcohol protons to the respective ester carbonyl groups. The established planar structure of **1** was further supported by the COSY and HMBC spectra as shown in Figure 1. The relative configuration of **1** at the stereogenetic centers was proved the same as that of **18** by a NOESY experiment (Figure 2), showing a β configuration of H-7, H-12, H-14, Me-15, H-17, and CH_2_-20, and an α configuration of H-2, H-9, H-10, and Me-18. The geometry of the Δ^3^ double bond was assigned as *Z* based on the proton coupling constant between H-3 and H-4 (*J* = 10.1 Hz) while that of Δ^5^ was determined as *E* due to the NOESY correlation between H-6 and H_2_-16. The relative configuration of **1** was thus determined as (1R*,2S*,7S*,8S*,9S*,10S*,11R*,12R*,14S*,17R*).

As gemmacolide AS (**1**) contained the same lactone and diene chromophores as gemmacolide N (**18**) and they differed only in the nature of substitutions for R_4_ and R_5_, the ECD spectrum of gemmacolide N could therefore be used as an ECD reference for the configurational assignment of gemmacolide AS (**1**) and its analogues. Because the absolute configuration of gemmacolide N had been unambiguously determined by a TDDFT calculation of its solution ECD spectrum [11], the absolute configuration of **1** was therefore determined as (1*R*,2*S*,7*S*,8*S*,9*S*,10*S*,11*R*,12*R*,14*S*,17*R*) due to the congruent ECD curves for **1** and that of gemmacolide N (**18**).

Gemmacolide AT (**2**), a white amorphous powder, had a molecular formula of C_33_H_44_O_15_ as established by HRESI-MS. The ^1^H and ^13^C NMR spectra data (Table 1 and Table 2) of **2** were almost identical to those of gemmacolide AQ (**8**) [8], showing the same functional groups for both compounds. The isovaleryl group, however, was found to be attached to C-13 of **2** instead of C-14 in gemmacolide AQ based on the analysis of HMBC spectra (see Supplementary Information). The structure of **2** had the same relative and absolute stereochemistry as that of **8** assessed by the NOESY and ECD measurements.

**Table 2:** ^1^H NMR data for gemmacolides AS–AV (**1**–**4**) ^a^.

No.	1	2	3	4
2	5.67, d (9.7)	5.62, ov	5.55, d (9.7)	5.64, ov
3	5.60, t (10.1, 9.7)	5.61, ov	5.60, t (9.7, 10.0)	5.65, ov
4	6.28, d (10.1)	6.35, d (9.1)	6.28, d (10.0)	6.41, d (7.7)
6	5.75, d (8.6)	5.83, d (8.5)	5.75, d (8.5)	6.07, d (8.7)
7	4.98, d (8.6)	4.96, d (8.5)	4.98, d (8.5)	4.94, d (8.7)
9	4.80, br d (4.7)	4.74, br d (4.8)	4.80, br d (4.5)	4.75, br d (3.8)
10	3.68, br d (4.7)	3.61, br d (4.8)	3.56, br d (4.5)	3.61, ov
12	4.53, br s	4.90, br d (2.8)	3.47, m	4.92, br d (2.9)
13β	1.96, ov	5.10, t (2.7, 2.8)	3.95, q (3.6)	5.07, t (2.8, 2.9)
13α	2.29, ov			
14	4.96, br d (3.0)	5.22, br d (2.7)	5.30, m	5.18, br d (2.8)
15	1.05, s	1.14, s	1.08, s	1.14, s
16a	5.37, d (15.7)	4.47, br s	5.28, d (15.9)	4.67, d (13.5)
16b	4.68, d (15.7)	4.47, br s	4.69, d (15.9)	4.54, d (13.5)
17	2.30, ov	2.29, q (7.1)	2.31, ov	2.31, ov
19	1.16, d (7.1)	1.15, d (7.1)	1.17, d (7.1)	1.14, d (6.9)
20a	3.53, br d (2.4)	3.63, br d (2.5)	3.51, br d (2.8)	3.60, ov
20b	2.79, br d (2.4)	2.94, br d (2.5)	2.73, br d (2.8)	2.94, br d (2.0)
9-OAc	2.19, s	2.20, s	2.18, s	2.19, s
R_1_	1.95, s	1.99, s	1.97, s	4.16, d (16.8)
				4.02, d (16.8)
R_2_	2.11, s	2.16 s	No.	see 2′–5′
R_3_	No.	see 2′–5′	No.	1.93, s
R_4_	see 2′–5′	2.09 s	see 2′–5′	2.09, s
R_5_	see 2ʺ–5ʺ	No.	2.14, s	No.
2′a	2.28, ov	2.09, ov (×2)	2.30, ov (×2)	2.34, ov
2′b	2.01, ov			2.20, ov
3′	2.17, m	1.99, m	2.08, m	2.14, m
4′	0.99, d (6.6)	0.92, d (6.6) (×2 Me)	1.00, d (6.7)	0.99, d (6.8)
5′	0.99, d (6.6)		0.99, d (6.7)	1.01 (d, 6.8)
2ʺa	2.15, ov			
2ʺb	2.06, ov			
3ʺ	2.04, ov			
4ʺ	0.96, d (6.6)			
5ʺ	0.94, d (6.6)			

^a^ 400 MHz, in CDCl_3_, assignments made by ^1^H-^1^H COSY, HSQC, HMBC and NOESY.

Gemmacolide AU (**3**) was isolated as a white amorphous powder and found to have a molecular formula of C_31_H_42_O_14_ established by HRESI-MS. ^1^H and ^13^C NMR spectra of **3** were similar to those of compound gemmacolide AR (**19**) [8]. The acetyl group at C-12 and C-13 in **19** was replaced by two hydroxy groups in **3**. This conclusion was supported by extensive 2D NMR (see Supplementary Information) and HRESIMS analysis. The structure of **3** was thus determined as (−)-(1*S*,2*S*,3*Z*,5*E*,7*S*,8*S*,9*S*,10*S*,11*R*,12*R*,13*S*,14*R*,17*R*) based on the ECD experiment.

Gemmacolide AV (**4**) was obtained as a white amorphous powder. Its HRESI-MS demonstrated the same molecular formula as C_33_H_43_O_15_Cl by HRESI-MS. ^1^H and ^13^C NMR spectroscopic data of **1** showed great similarity to those of gemmacolide AJ (**9**) [8], the only difference of the isovaleric acetyl at C-2 in **9** being replaced by a glycolyl group in **4**. The location of the glycolyl group at C-2 was indicated by the distinct HMBC correlations (see Supplementary Information). The established structure of **4** was further supported by detailed analysis of its 2D NMR data. Its absolute configuration was proven the same as that of **9** based on their similar ECD spectrum.

Gemmacolide AW (**5**) was found to be a white amorphous powder, having the molecular formula C_38_H_51_ClO_16_ based on the HRESIMS data. The ^1^H and ^13^C NMR data of **5** greatly resembled those of gemmacolide L (**12**) [10]. However, the substitutions of isovaleric acetyl group at C-12 and acetyl group at C-14 in **12** had to be interchanged in **5** based on the HMBC experiment (see Supplementary Information). The relative configuration for all chiral centers remained intact due to the NOEY experiment. Its absolute configuration was proved the same as that of **12** due to their similarity in ECD spectrum.

Gemmacolide AX (**6**) was isolated as a white amorphous powder. The molecular formula C_26_H_33_ClO_11_ was established by HRESI-MS. Comparison of overall ^1^H and ^13^C NMR data (Table 1 and Table 3) of **6** with those of gemmacolide X (**13**) [9] revealed great similarity except for the absence of two acetyl group. The acetyl group at C-9 and C-12 in **13** was replaced by a hydroxy group and a hydrogen atom in **6**. This conclusion was supported by extensive 2D NMR analysis. The relative configuration of all the asymmetric centers was determined as (1R*,2R*,3R*,4R*,6S*,7R*,8R*,9S*,10S*,11R*,14S*,17R*) based on a NOESY experiment (see Supplementary Information). The absolute configuration of **6** was tentatively suggested as (1*R*,2*R*,3*R*,4*R,*6*S*,7*R*,8*R*,9*S*,10*S*,11*R*,14*S*,17*R*) (Chart 1) due to its biogenetic correlation with compounds **1**–**5**.

**Table 3:** ^1^H NMR data for gemmacolides AW–AY (**5**–**7**) ^a^.

Proton	5	6	7
2	6.24, d (10.1)	5.38, d (6.8)	5.90, ov
3β	5.74, ov	6.13, dd (6.8, 10.6)	2.07, ov
3α			2.11, ov
4	6.00, d (11.5)	4.43, d (10.6)	4.43, ov
6	5.10, br s	5.48, br d (3.1)	4.43, ov
7	4.72, br d (3.6)	4.57, br d (3.1)	4.43, ov
9	4.95, br s	4. 37, br d (6.8)	5.79, br s
10	3.96, br s	2.44, br d (6.8)	3.62, br s
12	4.88, br d (3.4)	2.19, ov (×2)	4.61, br s
13β	5.23, t (3.4)	1.92, ov (×2)	2.19, ov
13α			2.09, ov
14	5.28, br s	4.97, br s	4.96, br s
15	1.31, s	1.33, s	1.21, s
16a	5.72, br s	5.54, br d (2.0)	6.04, br s
16b	5.70, br s	5.31, br d (2.0)	5.81, br s
17	2.93, ov	2.59, q (7.1)	2.99, ov
19	1.22, d (7.6)	1.30, d (7.1)	1.26, d (6.4)
20a	2.92, br d (2.9)	2.72, br d (3.0)	2.86, br d (2.1)
20b	2.64, br d (2.9)	2.82, br d (3.0)	2.35, br d (2.1)
9-OAc	2.17, s		2.23, s
R_1_	4.59, d (15.6)	2.04, s	2.03, s
	4.44, d (15.6)		
	2.28, ov		
	2.15, ov		
	0.98, d (6.6) (×2 Me)		
R_2_	2.11, s	1.98, s	3.08, ov
R_3_	1.95, s	No.	2.00, s
R_4_	2.25, m		2.18, ov
	2.15, m		2.06, ov
	2.15, m		2.04, ov
	0.98, d (6.6) (×2 Me)		0.98, d (6.3)
			0.94, d (6.3)
R_5_		2.07, s	

^a^ 400 MHz, in CDCl_3_, assignments made by ^1^H-^1^H COSY, HSQC, HMBC and NOESY.

Gemmacolide AY (**7**) was obtained as a white amorphous powder. The molecular formula C_31_H_43_ClO_13_ was established by HRE-SIMS. Comparison of overall ^1^H and ^13^C NMR data (Table 1 and Table 3) of **7** with those of gemmacolide U (**20**) [9] revealed great similarity with the only difference of the acetyl group at C-4 in **20** being replaced by a hydroxy group. This conclusion was supported by extensive 2D NMR and HRESIMS analysis (see Supplementary Information). The absolute configuration of **20** was tentatively assigned as that drawn in Chart 1 due to its biogenetic correlation with the co-isolates.

Compounds **1**–**7** were evaluated for their tumor cell growth inhibitory activity against A549 and MG63 cells. Compounds **4** and **7** showed moderate growth inhibitory effect against A549 while compound **5** displayed a promising growth inhibitory activity toward MG63 (Table 4).

**Table 4:** Tumor cell growth inhibitory activity of compounds **1**–**7** (IC_50_ in μM).

	1	2	3	4	5	6	7	Adriamycin ^a^
A549	>30.0	>30.0	>30.0	25.3	>30.0	>30.0	24.6	3.6
MG63	>30.0	>30.0	>30.0	>30.0	7.2	>30.0	>30.0	2.6

^a^ Positive control.

## 3. Experimental Section

### 3.1. General Experimental Procedures

Commercial silica gel (Yantai, China, 200–300; 400–500 mesh) and RP silica gel (Merck, Darmstadt, Germany, 43–60 μm) were used for column chromatography (CC). Precoated silica gel plates (Yantai, China, HSGF**-**254) and RP silica gel (Macherey-Nagel, Düren, Germany, RP-18 F254) were used for analytical thin-layer chromatography (TLC). Spots were detected on TLC under UV or by heating after spraying with anisaldehyde-sulphuric acid reagent. The NMR spectra were recorded at 300 K on a Bruker DRX 400 spectrometer (Bruker Biospin Inc., Ettlingen, Germany). Chemical shifts are reported in parts per million (δ), with use of the residual CHCl_3_ signal (δ_H_ 7.26 ppm) as an internal standard for ^1^H NMR and CDCl_3_ (δ_C_ 77.0 ppm) for ^13^C NMR; Coupling constants (*J*) are reported in Hz. ^1^H NMR and ^13^C NMR assignments were complemented ^1^H-^1^H COSY, HSQC, HMBC and NOESY experiments. The following abbreviations are used to describe spin multiplicity: s = singlet, d = doublet, t = triplet, q = quartet, m = multiplet, br s = broad singlet, dd = doublet of doublets, ov = overlapped signals. Optical rotations were measured in CHCl_3_ with an Autopol IV polarimeter (Rudolph, Flanders, NJ, USA) at the sodium D line (590 nm). Infrared spectra were recorded in thin polymer films on a Nexus 470 FT-IR spectrophotometer (Nicolet, Madison, WI, USA); peaks are reported in cm^−1^. UV absorption spectra were recorded on a Varian Cary 100 UV-Vis spectrophotometer (Varian, Palo Alto, CA, USA); peaks wavelengths are reported in nm. Circular dichroism spectra were recorded with a JASCO J-715 circular dichroism spectropolarimeter (JASCO, Mary’s Court Easton, MD, USA). The MS and HRMS were performed on a Q-TOF Micro mass spectrometer (Bruker, Bremen, Germany), resolution 5000. An isopropyl alcohol solution of sodium iodide (2 mg/mL) was used as a reference compound. Semi**-**preparative RP-HPLC was performed on an Agilent 1100 system (Agilent Technologies, Santa Clara, CA, USA) equipped with a refractive index detector using an YMC Pack ODS-A column (particle size 5 μm, 250 × 10 mm, YMC Co., Ltd., Kyoto, Japan).

### 3.2. Animal Material

The gorgonian coral *Dichotella* *gemmacea.* (2.0 kg, wet weight) was collected from the South China Sea (20°54′ N, 109°05′ E), in August 2007 and identified by Dr. Xiu-Bao Li, the South China Sea Institute of Oceanology, Academia Sinica. A voucher specimen was deposited in the Second Military Medical University, Shanghai, China.

### 3.3. Extraction and Isolation

The frozen specimen was extracted with acetone and methanol (3 × 2.0 L) by ultrasonication. The solvent was combined and removed *in vacuo*. The resultant residue was partitioned between H_2_O and EtOAc. The layers were separated, and then EtOAc was removed to afford 14.0 g of residue. The crude extract was further partitioned between MeOH and hexane, affording 10.0 g from the MeOH fraction. This residue was subjected to silicagel CC and eluted with hexane/acetone (from 100:0 to 0:100) as eluent. Fraction 11 was further fractionated by RP-silical gel CC (MeOH/H_2_O, 20:80 to 75:25, in 5% increments) to give three subfractions (A–C). Subfraction B was purified by HPLC (MeOH/H_2_O, 55:45, 1.0 mL·min^−1^) to yield **4** (2.9 mg, 40.8 min), **2** (2.0 mg, 36.5 min) and **7** (0.6 mg, 34.2 min). Fraction 10 was purified by HPLC (MeOH/H_2_O, 58:42, 1.2 mL·min^−1^), yielding **14** (10.3 mg, 36.4 min). Fraction 9 were repeatedly subjected to silica gel and Sephadex LH-20 CC, and then purified by HPLC (MeOH/H_2_O, 60:40, 1.5 mL·min^−1^) to yield **3** (0.7 mg, 25.3 min), **6** (0.5 mg, 28.5 min), **8** (1.1 mg, 32.6 min), and **17** (2.4 mg, 35.4 min). Fraction 8 was further fractionated by RP-silical gel CC (gradient elution from MeOH//H_2_O, 3:7 to MeOH, in 5% increments) and purified by HPLC (MeOH/H_2_O, 67:33, 1.2 mL·min^−1^), yielding **1** (2.3 mg, 36.4 min), **9** (4.9 mg, 40.1 min), **10** (0.5 mg, 28.5 min), **15** (1.1 mg, 45.6 min), and **16** (2.4 mg, 49.4 min). Fraction 7 was purified by HPLC (MeOH/H_2_O, 60:40, 1.5 mL·min^−1^), yielding **5** (7.0 mg, 49.4 min), **11** (4.3 mg, 56.2 min), **12** (52.7 mg, 36.4 min).

**Gemmacolide AS (1)**: white amorphous powder; ${\left[\alpha \right]}_{\text{D}}^{\text{24}}$ = −31 (*c* 0.13, CHCl_3_); UV (MeOH) 204 nm; CD (CH_3_CN, *c* 3.0 × 10^−4^) λ_max_ (Δε) positive below 195 nm, 197 (−15.41) nm; IR (film) ν_max_ 3478, 1778, 1739 cm^−1^; ^1^H NMR spectroscopic data, see Table 2; ^13^C NMR spectroscopic data, see Table 1; ESI-MS *m/z* 729 [M + Na]^+^; HRESI-MS *m/z* 729.3094 [M + Na]^+^ (calcd for C_36_H_50_O_14_Na, 729.3098).

**Gemmacolide AT (2)**: white amorphous powder; ${\left[\alpha \right]}_{\text{D}}^{\text{24}}$ = −20 (*c* 0.075, CHCl_3_); UV (MeOH) 207 nm; CD (CH_3_CN, *c* 1.3 × 10^−3^) λ_max_ (Δε) positive below 190 nm, 201 (−14.26) nm, 217 (−13.70) nm; IR (film) ν_max_ 3468, 1774, 1743 cm^−1^; ^1^H NMR spectroscopic data, see Table 2; ^13^C NMR spectroscopic data, see Table 1; ESI-MS *m/z* 703 [M + Na]^+^; HRESI-MS *m/z* 703.2576 [M + Na]^+^ (calcd for C_33_H_44_O_15_Na, 703.2578).

**Gemmacolide AU (3)**: white amorphous powder; ${\left[\alpha \right]}_{\text{D}}^{\text{24}}$ = −0 (*c* 0.04, CHCl_3_); UV (MeOH) 204 nm; CD (CH_3_CN, *c* 3.6 × 10^−4^) λ_max_ (Δε) positive below 190 nm, 217.5 (−10.22) nm; IR (film) ν_max_ 3467, 1777, 1740 cm^−1^; ^1^H NMR spectroscopic data, see Table 2; ^13^C NMR spectroscopic data, see Table 1; ESI-MS *m/z* 661 [M + Na]^+^; HRESI-MS *m/z* 661.2470 [M + Na]^+^ (calcd for C_33_H_44_O_16_Na, 661.2472).

**Gemmacolide AV (4)**: white amorphous powder; ${\left[\alpha \right]}_{\text{D}}^{\text{24}}$ = −28 (*c* 0.15, CHCl_3_); UV (MeOH) 205, 273 nm; CD (CH_3_CN, *c* 1.3 × 10^−3^) λ_max_ (Δε) positive below 190 nm, 199.5 (−28.02) nm; IR (film) ν_max_ 3406, 1778, 1745 cm^−1^; ^1^H NMR spectroscopic data, see Table 2; ^13^C NMR spectroscopic data, see Table 1; ESI-MS *m/z* 737 [M + Na]^+^; HRESI-MS *m/z* 737.2195 [M + Na]^+^ (calcd for C_33_H_43_O_15_ClNa, 737.2188).

**Gemmacolide AW (5)**: white amorphous powder; ${\left[\alpha \right]}_{\text{D}}^{\text{24}}$ = −104 (*c* 0.5, CHCl_3_); UV (MeOH) 203 nm; CD (CH_3_CN, *c* 2.6 × 10^−4^) λ_max_ (Δε) positive below 197 nm, 194 (−31.04) nm, 219 (−21.22) nm; IR (film) ν_max_ 3550, 1782, 1744 cm^−1^; ^1^H NMR spectroscopic data, see Table 3; ^13^C NMR spectroscopic data, see Table 1; ESI-MS *m/z* 821 [M + Na]^+^; HRESI-MS *m/z* 821.2759 [M + Na]^+^ (calcd for C_38_H_51_O_16_ClNa, 821.2763).

**Gemmacolide AX (6)**: white amorphous powder; ${\left[\alpha \right]}_{\text{D}}^{\text{24}}$ = 0 (*c* 0.02, CHCl_3_); UV (MeOH) 202 nm; CD (CH_3_CN, *c* 1.4 × 10^−4^) λ_max _(Δε) positive below 191 nm, 208.5 (−9.64) nm; IR (film) ν_max_ 3467, 1787, 1743 cm^−1^; ^1^H NMR spectroscopic data, see Table 3; ^13^C NMR spectroscopic data, see Table 1; ESI-MS *m/z* 579 [M + Na]^+^; HRESI-MS *m/z* 579.1613 [M + Na]^+^ (calcd for C_26_H_33_O_11_ClNa, 579.1609).

**Gemmacolide AY (7)**: white amorphous powder; ${\left[\alpha \right]}_{\text{D}}^{\text{24}}$ = 0 (*c* 0.03, CHCl_3_); UV (MeOH) 204 nm; CD (CH_3_CN, *c* 1.4 × 10^−4^) λ_max _(Δε) negative below 191 nm, 209 (15.06) nm; IR (film) ν_max_ 3488, 1788, 1742 cm^−1^; ^1^H NMR spectroscopic data, see Table 3; ^13^C NMR spectroscopic data, see Table 1; ESI-MS *m/z* 681 [M + Na]^+^; HRESI-MS *m/z* 681.2293 [M + Na]^+^ (calcd for C_31_H_43_O_13_ClNa, 681.2290).

### 3.4. Cytotoxicity Assay

Compounds **1**–**7** were evaluated for cytotoxicity against human lung adenocarcinoma (A549) and human osteosarcoma cell (MG63), using a modification of the 3-(4,5-dimethylthiazol-2-yl)-2,5-diphenyltetrazolium bromide (MTT) colorimetric method [15]. Adriamycin was used as positive control.

## 4. Conclusions

This paper reported the isolation and structure elucidation of seven new briarane diterpenoids, gemmacolides AS–AY (**1**–**7**), from the gorgonian *D. gemmacea*. These compounds were tested for growth inhibition activity against A549 and MG63 cell lines. Compounds **4** and **7** showed a moderate growth inhibitory effect against A549 while compound **5** displayed a potential growth inhibitory activity toward MG63, being similar to that of positive control adriamycin. This research gives an additional example of briarane diterpenoids for their tumor growth inhibitory activity, and would continuously encourage further investigations on the chemistry and antitumor activity of this cluster of metabolites.

## Supplementary Files

Supplementary File 1
