# Supplementary material for: Briarane Diterpenoids from the Gorgonian Dichotella gemmacea
Source: Mar Drugs. 2014 Dec 18;12(12):6178–89. doi: 10.3390/md12126178 (PMC4278224; doi:10.3390/md12126178)
Supplement: Supplementary File 1 [file marinedrugs-12-06178-s001.pdf]

Supplementary Information

Index

1	Spectra of the new compound **1**.	Figures S1–S8
	HR-ESIMS spectrum of the new compound **1**	Figure S1
	^{1}H NMR spectrum of the new compound **1**	Figure S2
	^{13}C NMR spectrum of the new compound **1**	Figure S3
	DEPT spectrum of the new compound **1**	Figure S4
	HSQC spectrum of the new compound **1**	Figure S5
	^{1}H-^{1}H COSY spectrum of the new compound **1**	Figure S6
	HMBC spectrum of the new compound **1**	Figure S7
	NOESY spectrum of the new compound **1**	Figure S8
2	Spectra of the new compound **2**	Figures S9–S15
	HR-ESIMS spectrum of the new compound **2**	Figure S9
	^{1}H NMR spectrum of the new compound **2**	Figure S10
	^{13}C NMR spectrum of the new compound **2**	Figure S11
	DEPT spectrum of the new compound **2**	Figure S12
	HSQC spectrum of the new compound **2**	Figure S13
	HMBC spectrum of the new compound **2**	Figure S14
	NOESY spectrum of the new compound **2**	Figure S15
3	Spectra of the new compound **3**	Figures S16–S23
	HR-ESIMS spectrum of the new compound **3**	Figure S16
	^{1}H NMR spectrum of the new compound **3**	Figure S17
	^{13}C NMR spectrum of the new compound **3**	Figure S18
	DEPT spectrum of the new compound **3**	Figure S19
	HSQC spectrum of the new compound **3**	Figure S20
	^{1}H-^{1}H COSY spectrum of the new compound **3**	Figure S21
	HMBC spectrum of the new compound **3**	Figure S22
	NOESY spectrum of the new compound **3**	Figure S23
4	Spectra of the new compound **4**	Figures S24–S31
	HR-ESIMS spectrum of the new compound **4**	Figure S24
	^{1}H NMR spectrum of the new compound **4**	Figure S25
	^{13}C NMR spectrum of the new compound **4**	Figure S26
	DEPT spectrum of the new compound **4**	Figure S27
	HSQC spectrum of the new compound **4**	Figure S28
	^{1}H-^{1}H COSY spectrum of the new compound **4**	Figure S29
	HMBC spectrum of the new compound **4**	Figure S30
	NOESY spectrum of the new compound **4**	Figure S31
5	Spectra of the new compound **5**	Figures S32–S39
	HR-ESIMS spectrum of the new compound **5**	Figure S32
	^{1}H NMR spectrum of the new compound **5**	Figure S33
	^{13}C NMR spectrum of the new compound **5**	Figure S34
	DEPT spectrum of the new compound **5**	Figure S35
	HSQC spectrum of the new compound **5**	Figure S36
	^{1}H-^{1}H COSY spectrum of the new compound **5**	Figure S37
	HMBC spectrum of the new compound **5**	Figure S38
	NOESY spectrum of the new compound **5**	Figure S39

6	Spectra of the new compound **6**	Figures S40–S47
	HR-ESIMS spectrum of the new compound **6**	Figure S40
	^{1}H NMR spectrum of the new compound **6**	Figure S41
	^{13}C NMR spectrum of the new compound **6**	Figure S42
	DEPT spectrum of the new compound **6**	Figure S43
	HSQC spectrum of the new compound **6**	Figure S44
	^{1}H-^{1}H COSY spectrum of the new compound **6**	Figure S45
	HMBC spectrum of the new compound **6**	Figure S46
	NOESY spectrum of the new compound **6**	Figure S47
7	Spectra of the new compound **7**	Figures S48–S53
	HR-ESIMS spectrum of the new compound **7**	Figure S48
	^{1}H NMR spectrum of the new compound **7**	Figure S49
	^{13}C NMR spectrum of the new compound **7**	Figure S50
	DEPT spectrum of the new compound **7**	Figure S51
	HSQC spectrum of the new compound **7**	Figure S52
	^{1}H-^{1}H COSY spectrum of the new compound **6**	Figure S53
	HMBC spectrum of the new compound **6**	Figure S54
	NOESY spectrum of the new compound **7**	Figure S55

Figure S1. HR-ESIMS spectrum of the new compound **1**.

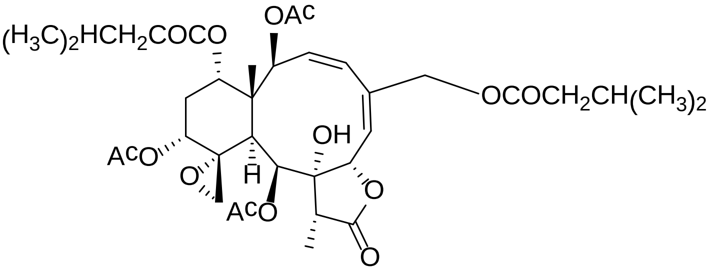

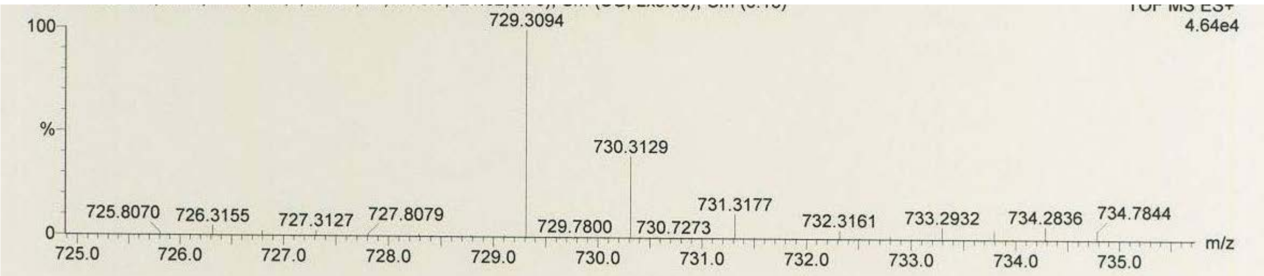

Minimum:	40.00				-1.5		
Maximum:	100.00		5.0	10.0	50.0		
Mass	RA	Calc. Mass	mDa	PPM	DBE	i-FIT	Formula
729.3094	100.00	729.3098	-0.4	-0.5	11.5	29.5	C36 H50 O14 Na

Figure S2. ^{1}H NMR spectrum of the new compound **1**.

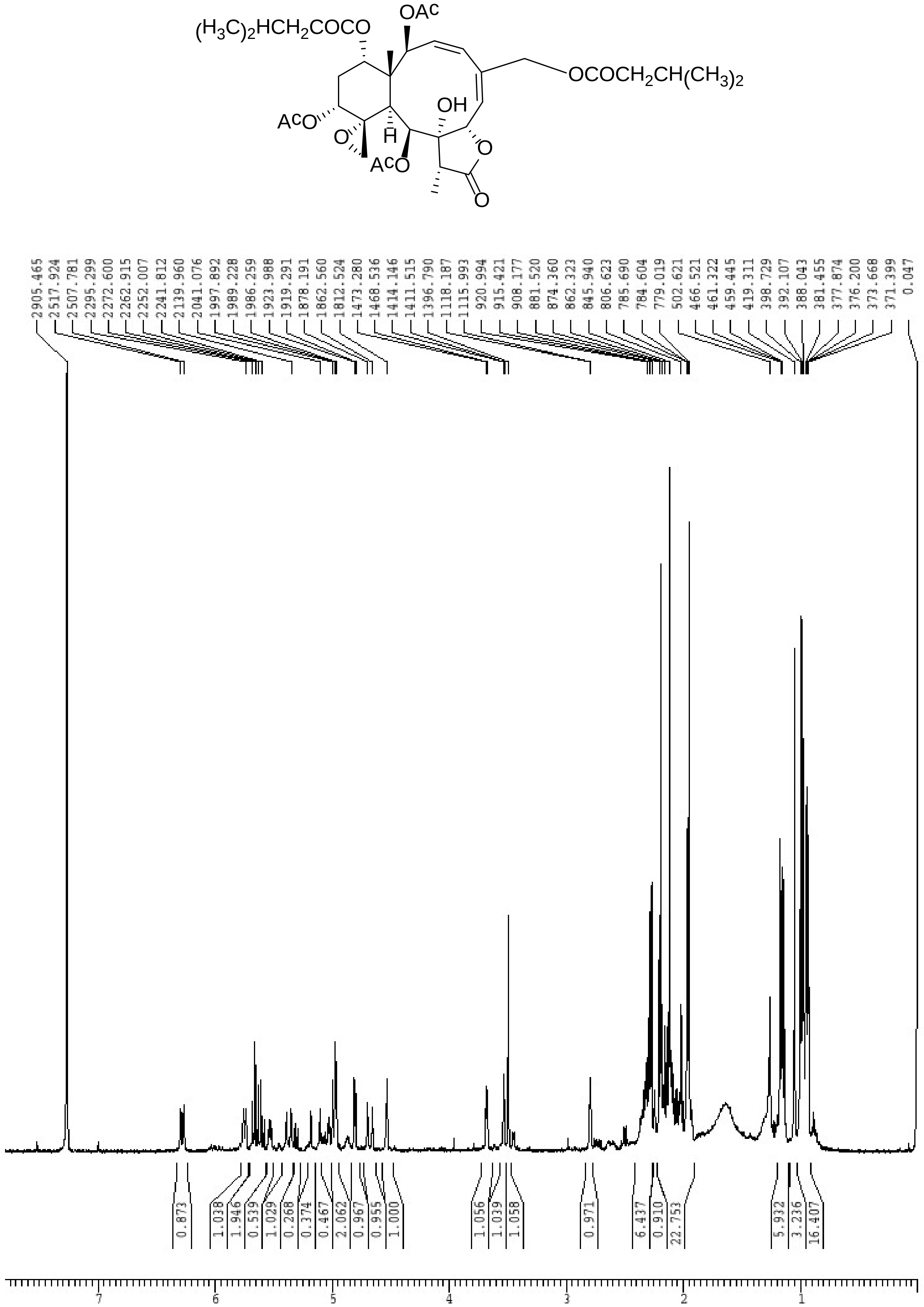

Figure S3. ^{13}C NMR spectrum of the new compound **1**.

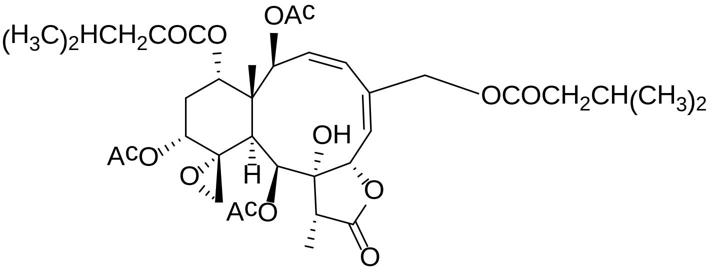

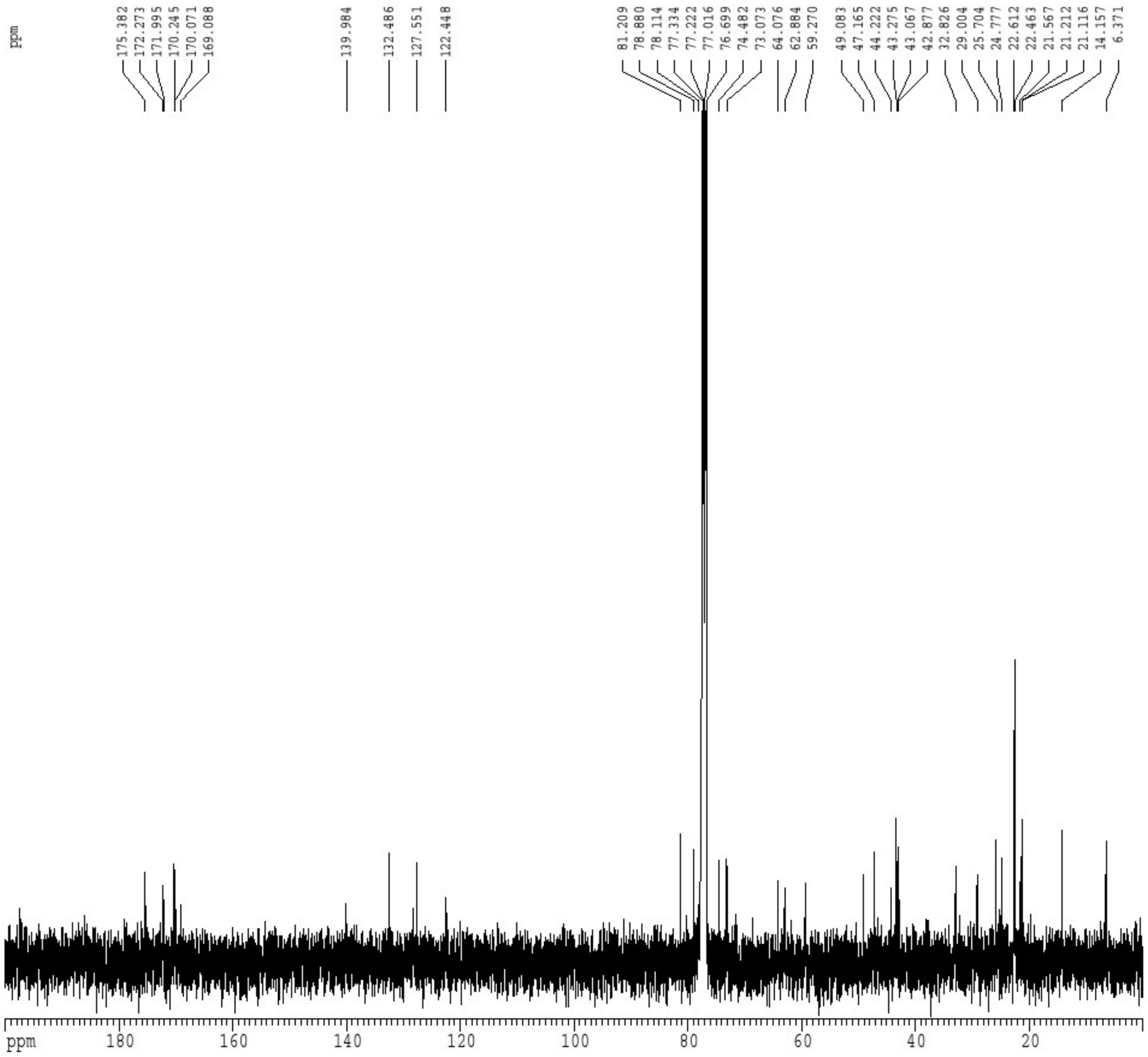

Figure S4. DEPT spectrum of the new compound **1**.

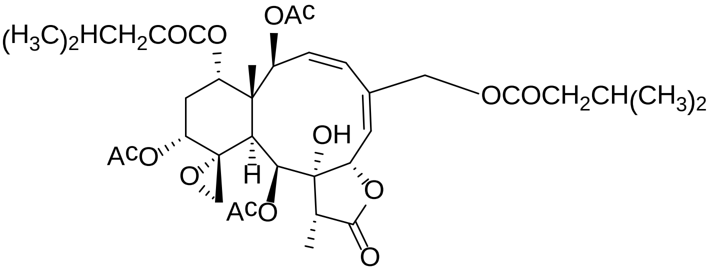

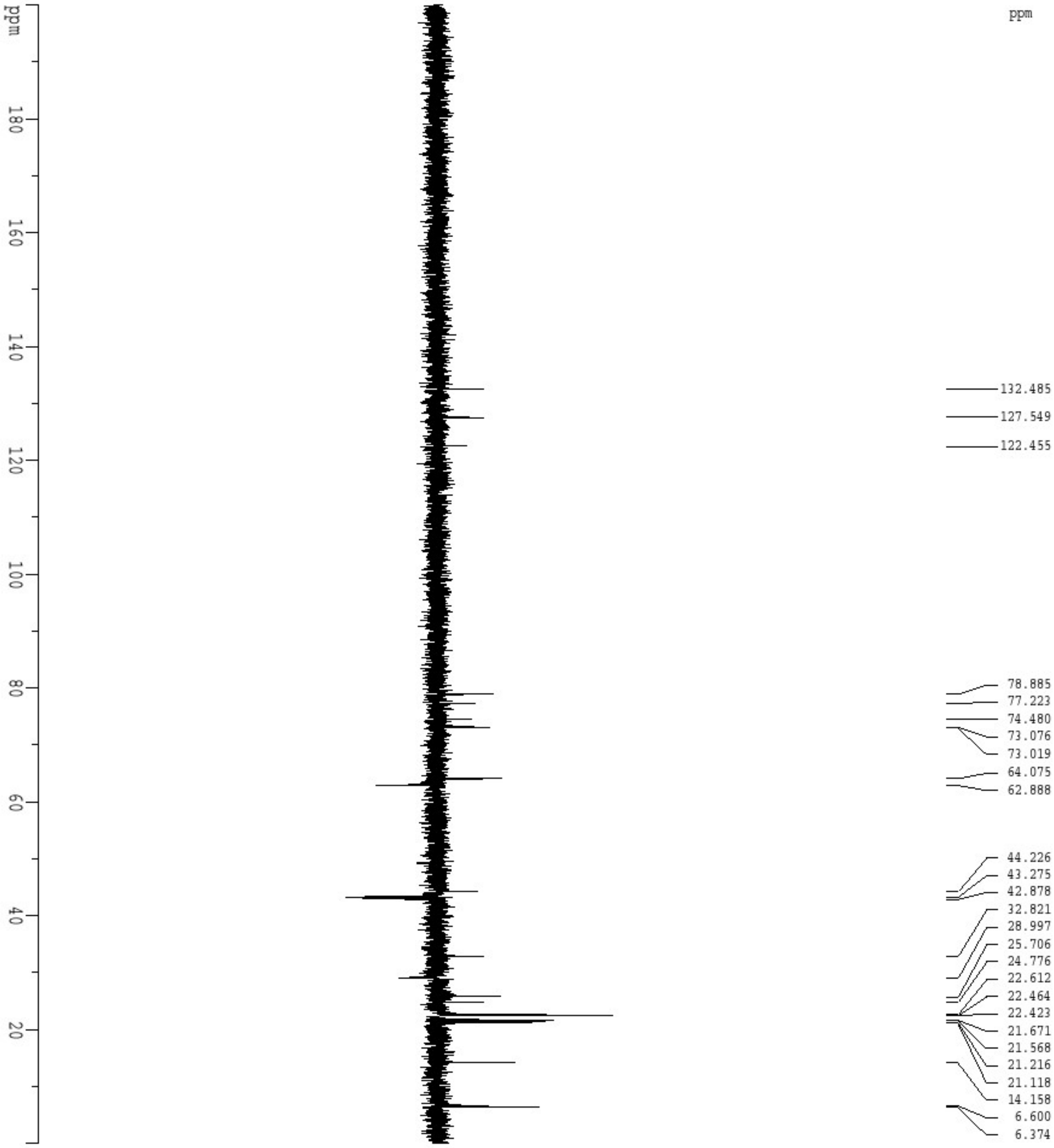

Figure S5. HSQC spectrum of the new compound **1**.

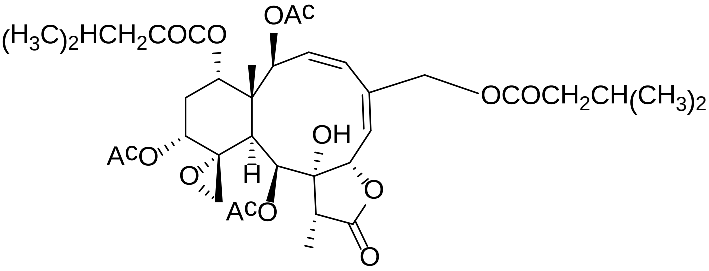

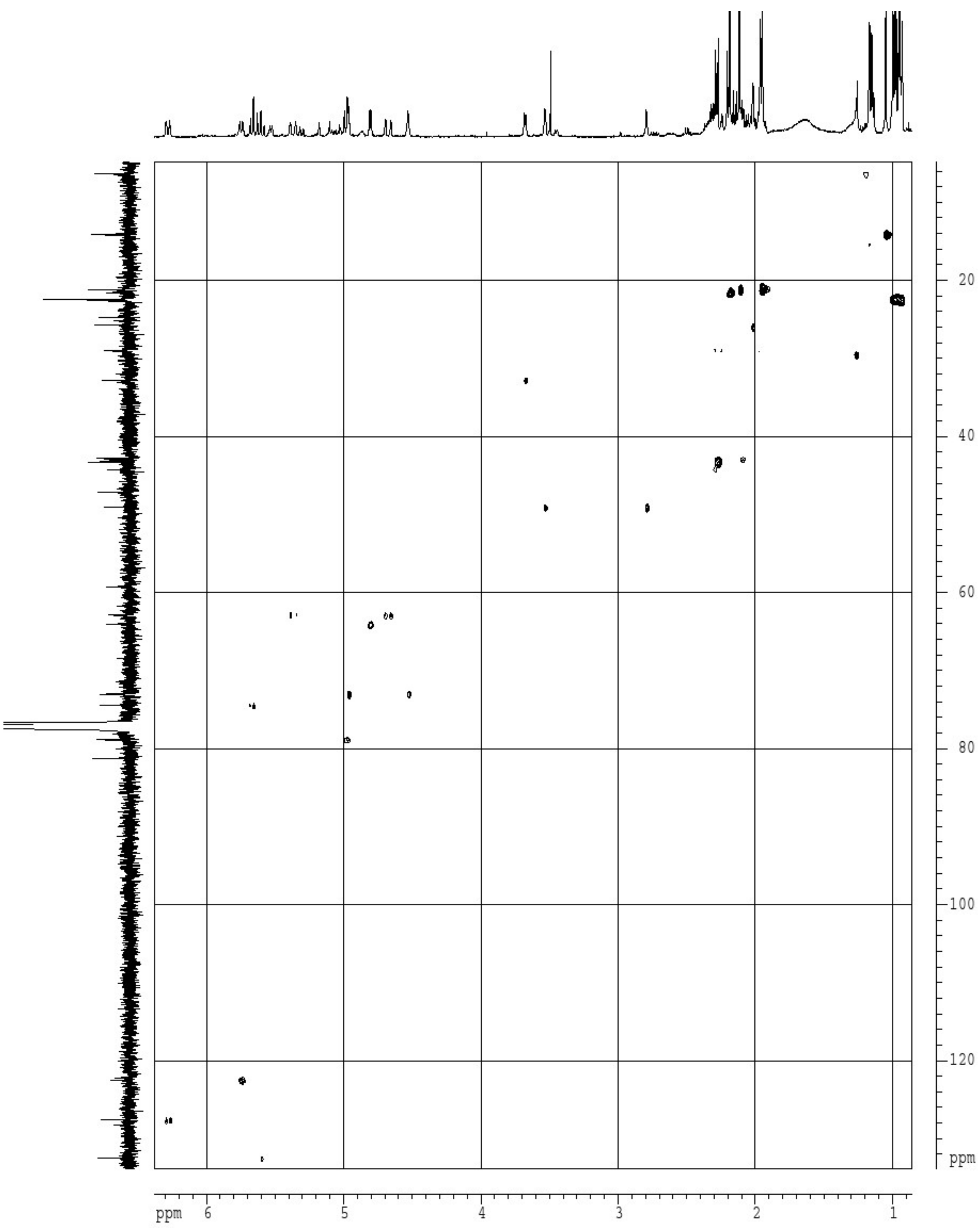

Figure S6. ^1H-^1H COSY spectrum of the new compound **1**.

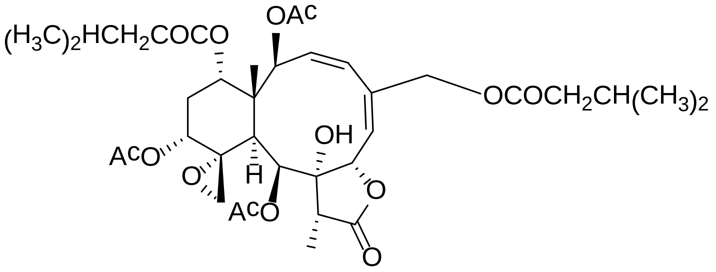

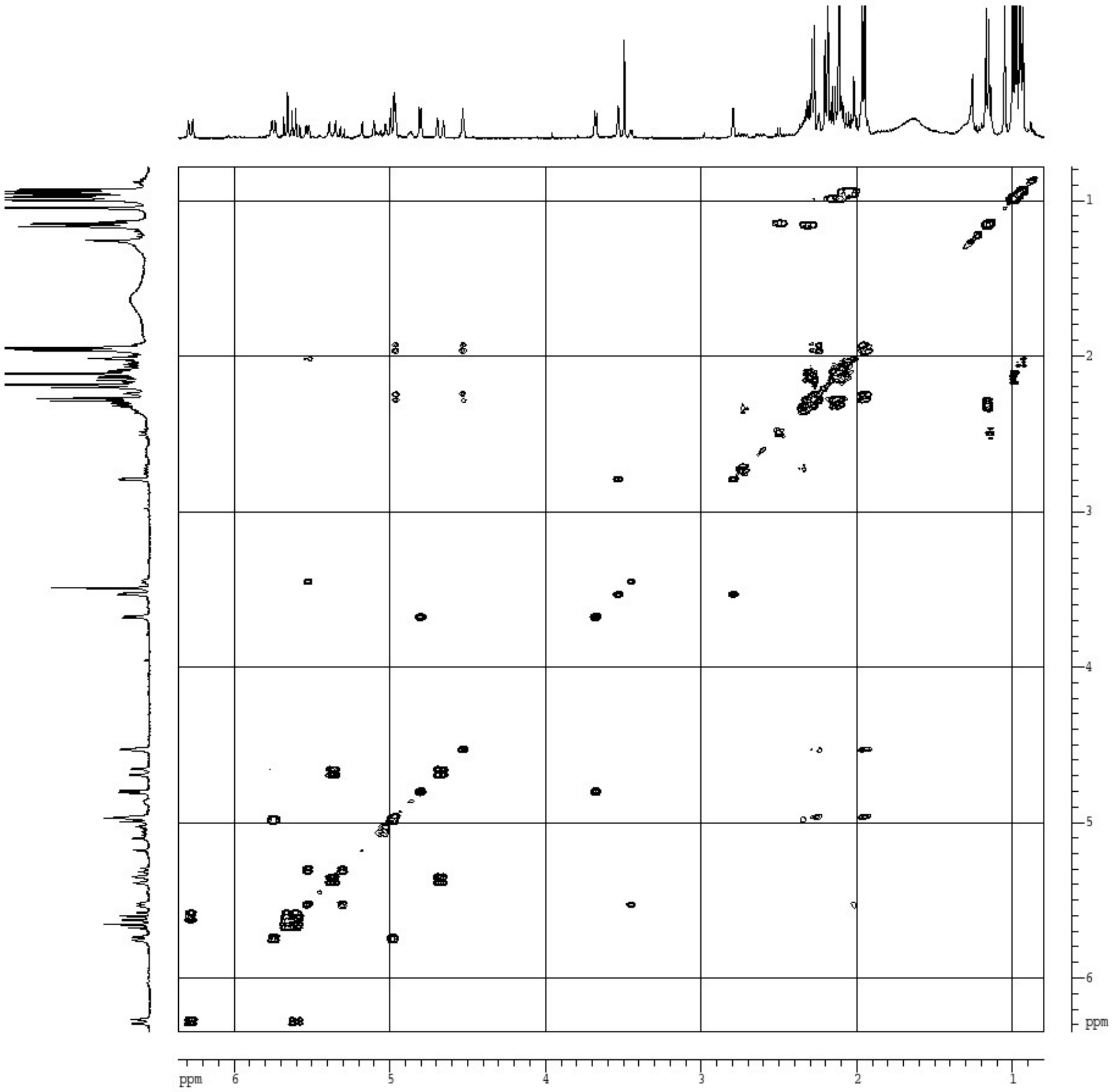

Figure S7. HMBC spectrum of the new compound **1**.

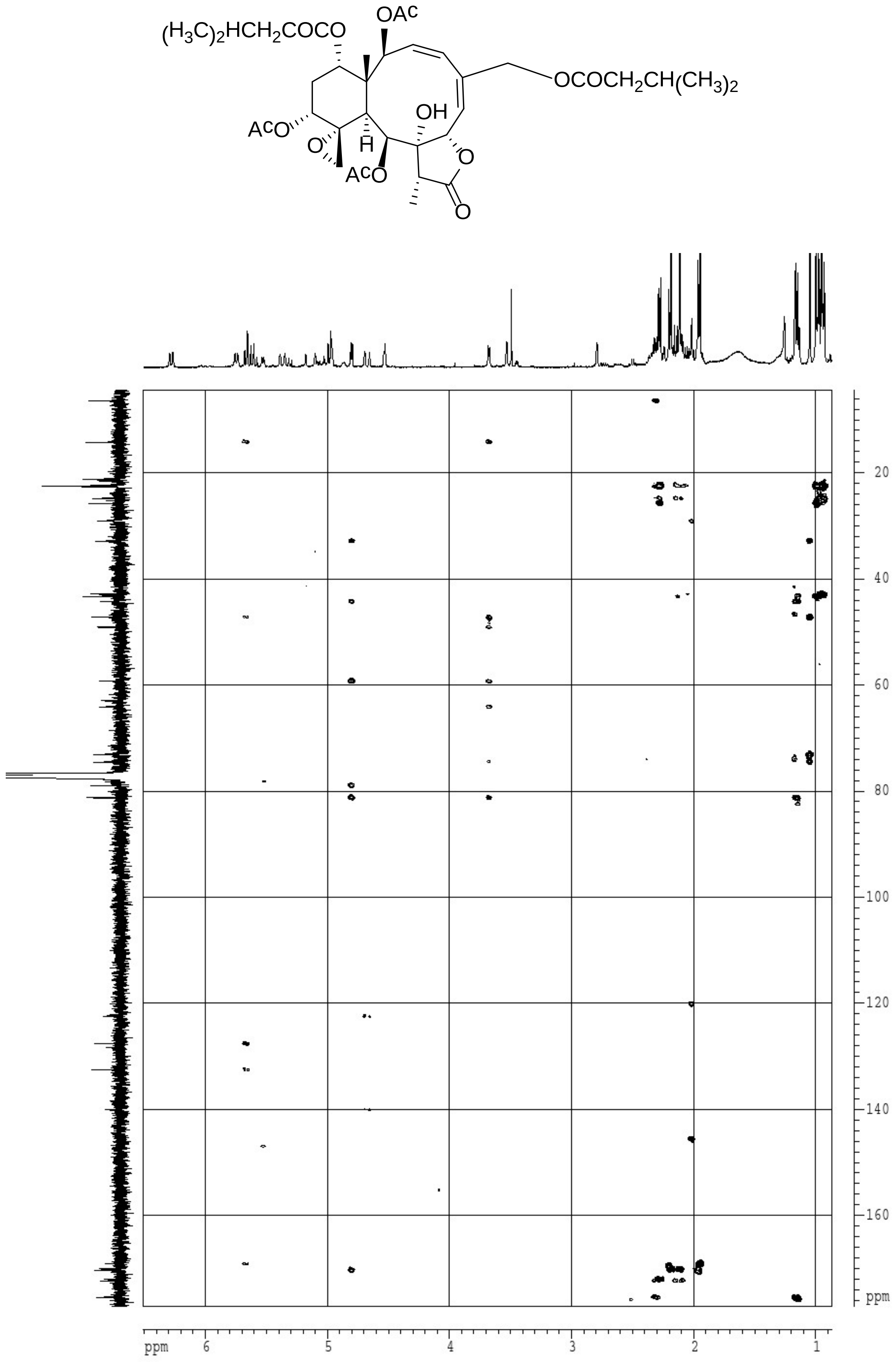

Figure S8. NOESY spectrum of the new compound **1**.

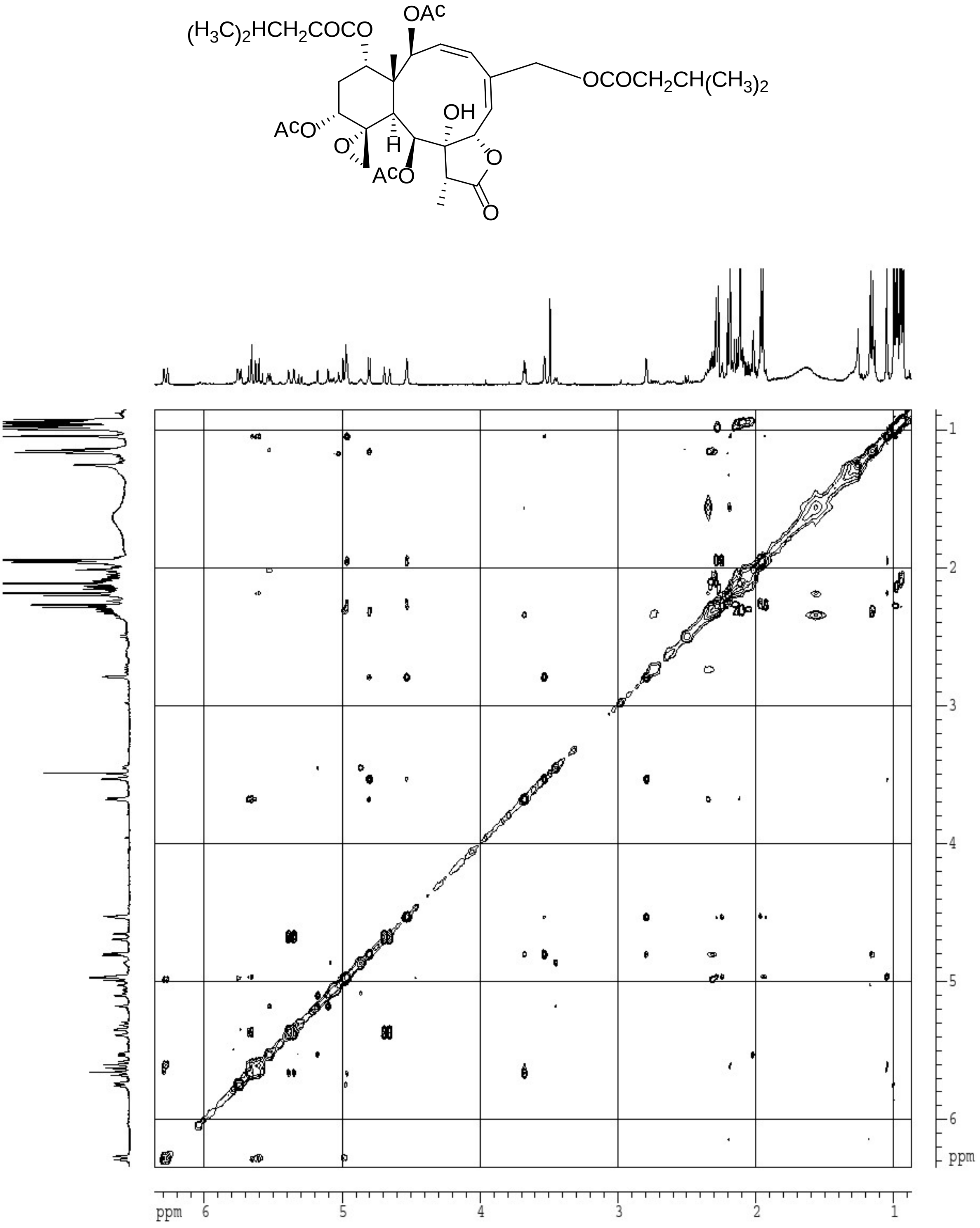

Figure S9. HR-ESIMS spectrum of the new compound **2**.

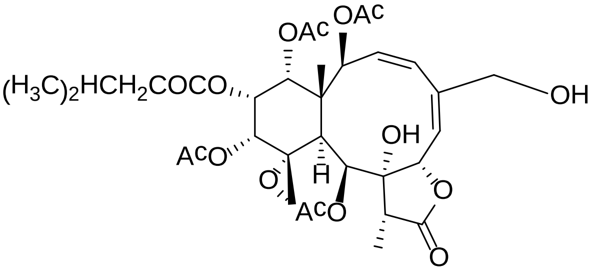

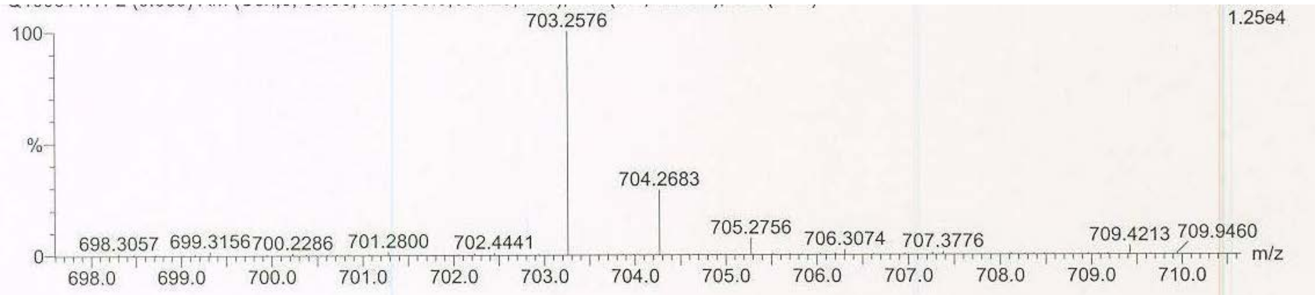

Minimum:	35.00				-1.5		
Maximum:	100.00		5.0	10.0	50.0		
Mass	RA	Calc. Mass	mDa	PPM	DBE	i-FIT	Formula
703.2576	100.00	703.2578	-0.2	-0.3	11.5	150.9	C33 H44 O15 Na

Figure S10. ^{1}H NMR spectrum of the new compound **2**.

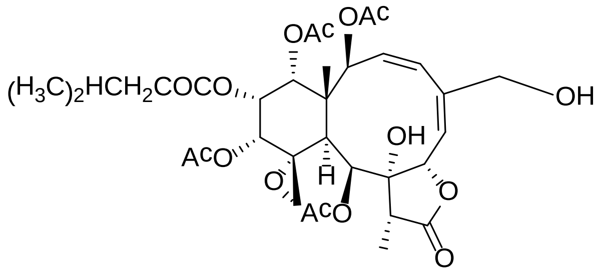

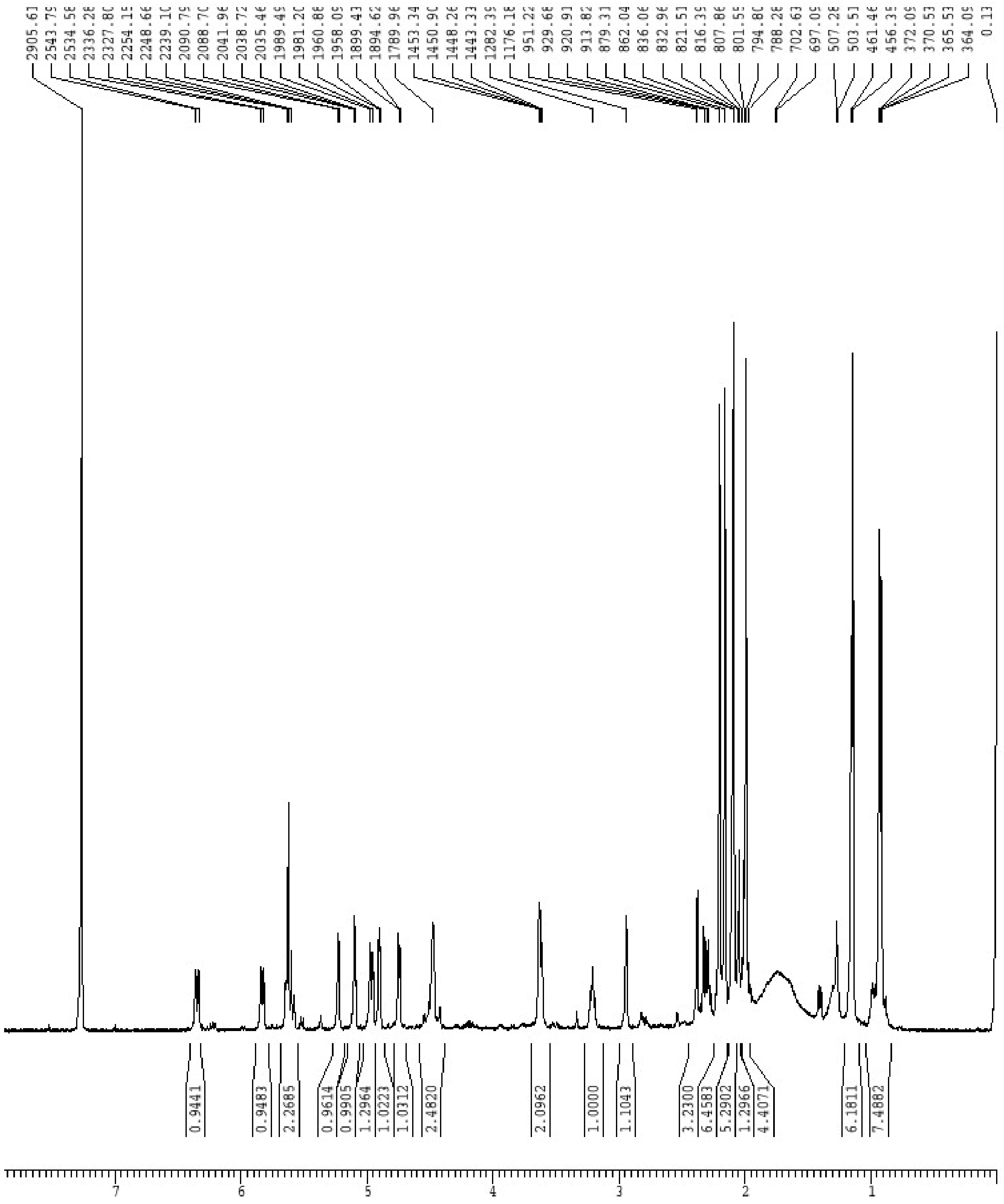

Figure S11. ^{13}C NMR spectrum of the new compound **2**.

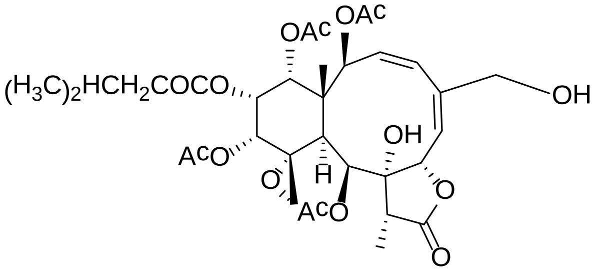

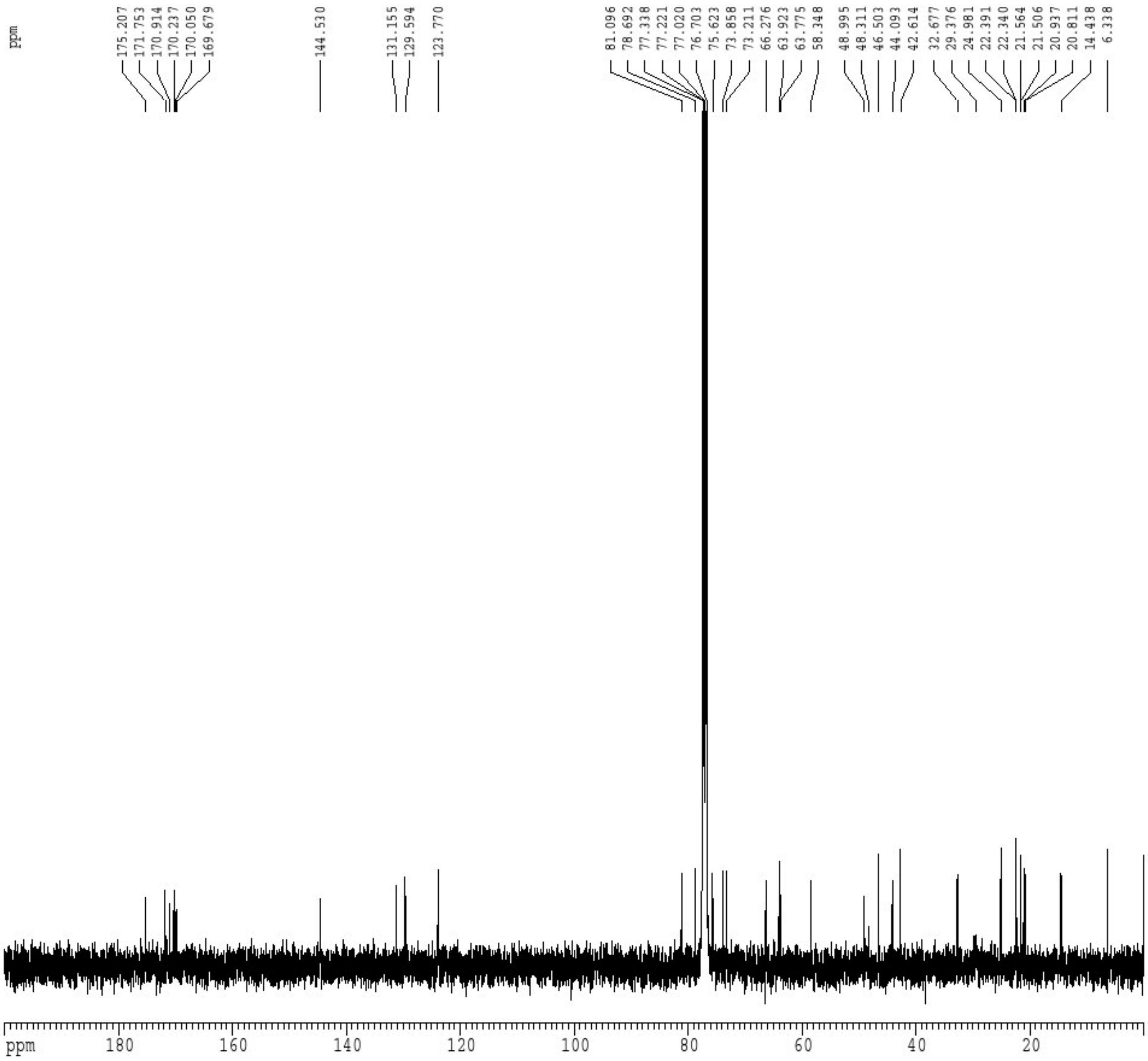

Figure S12. DEPT spectrum of the new compound **2**.

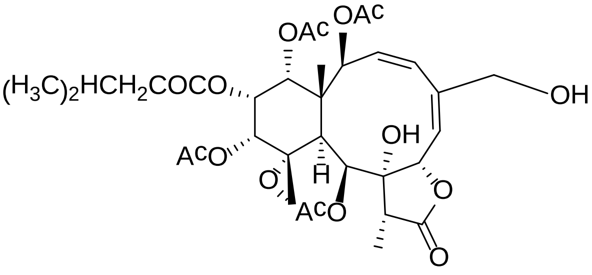

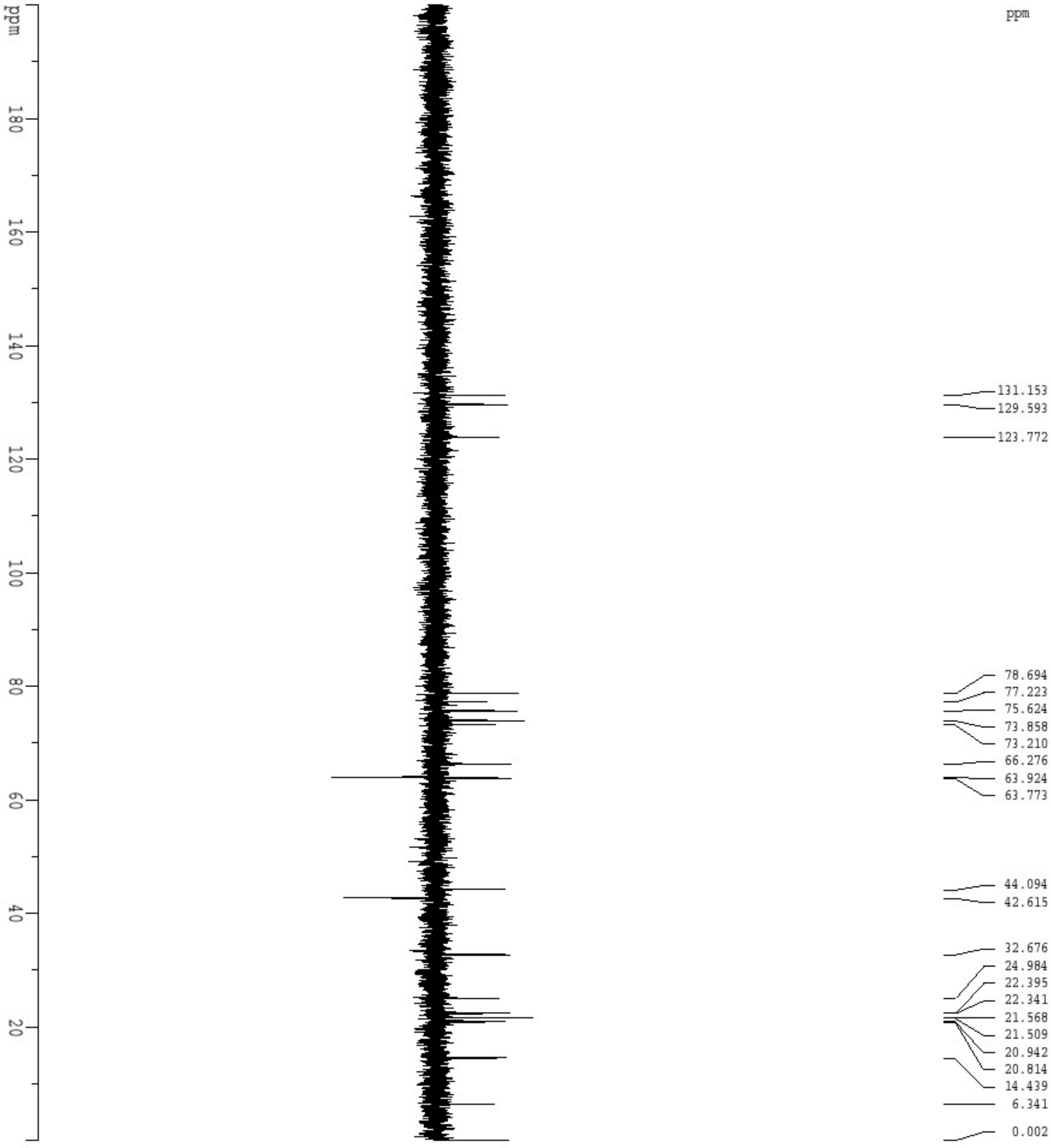

Figure S13. HSQC spectrum of the new compound **2**.

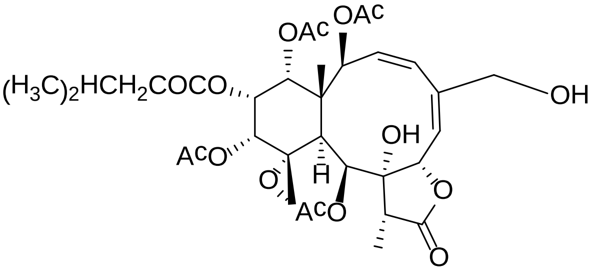

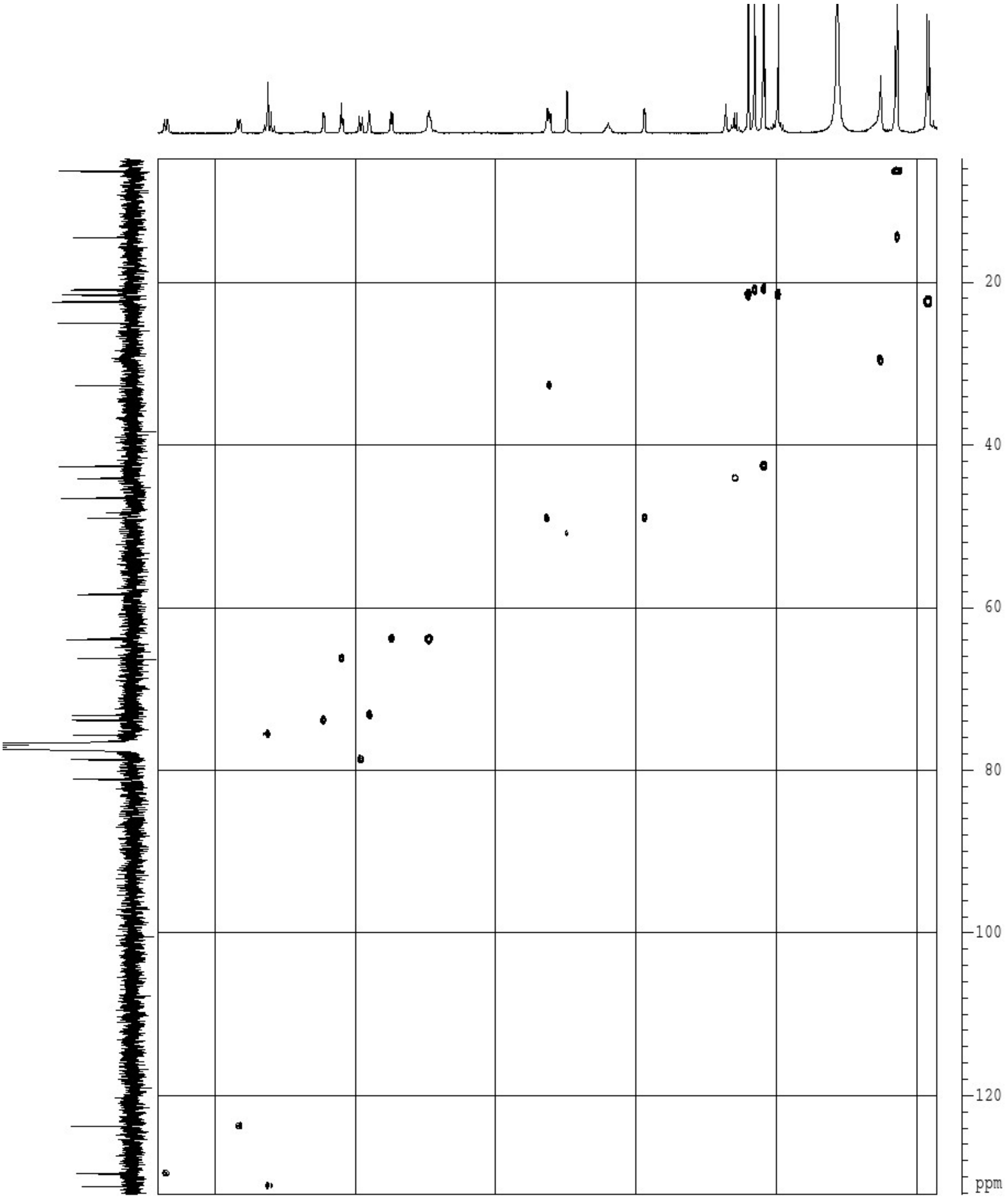

Figure S14. HMBC spectrum of the new compound **2**.

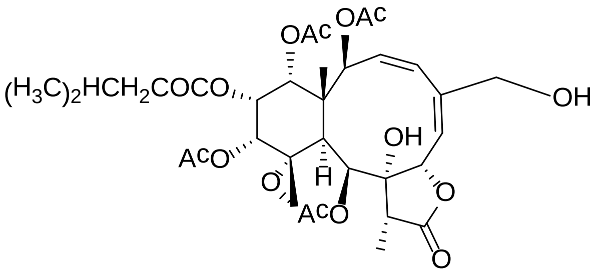

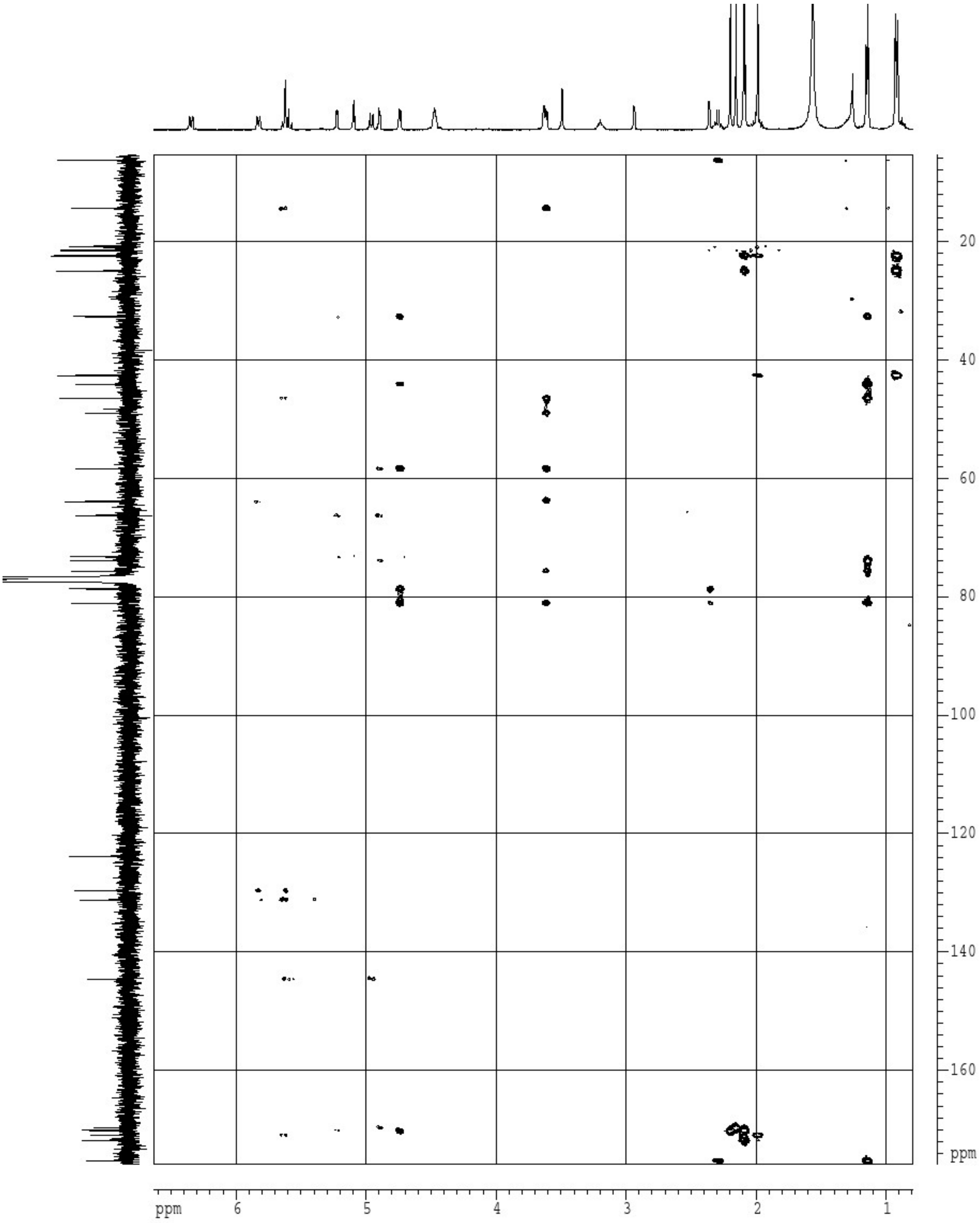

Figure S15. NOESY spectrum of the new compound **2**.

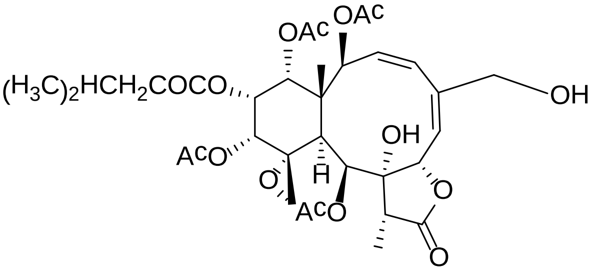

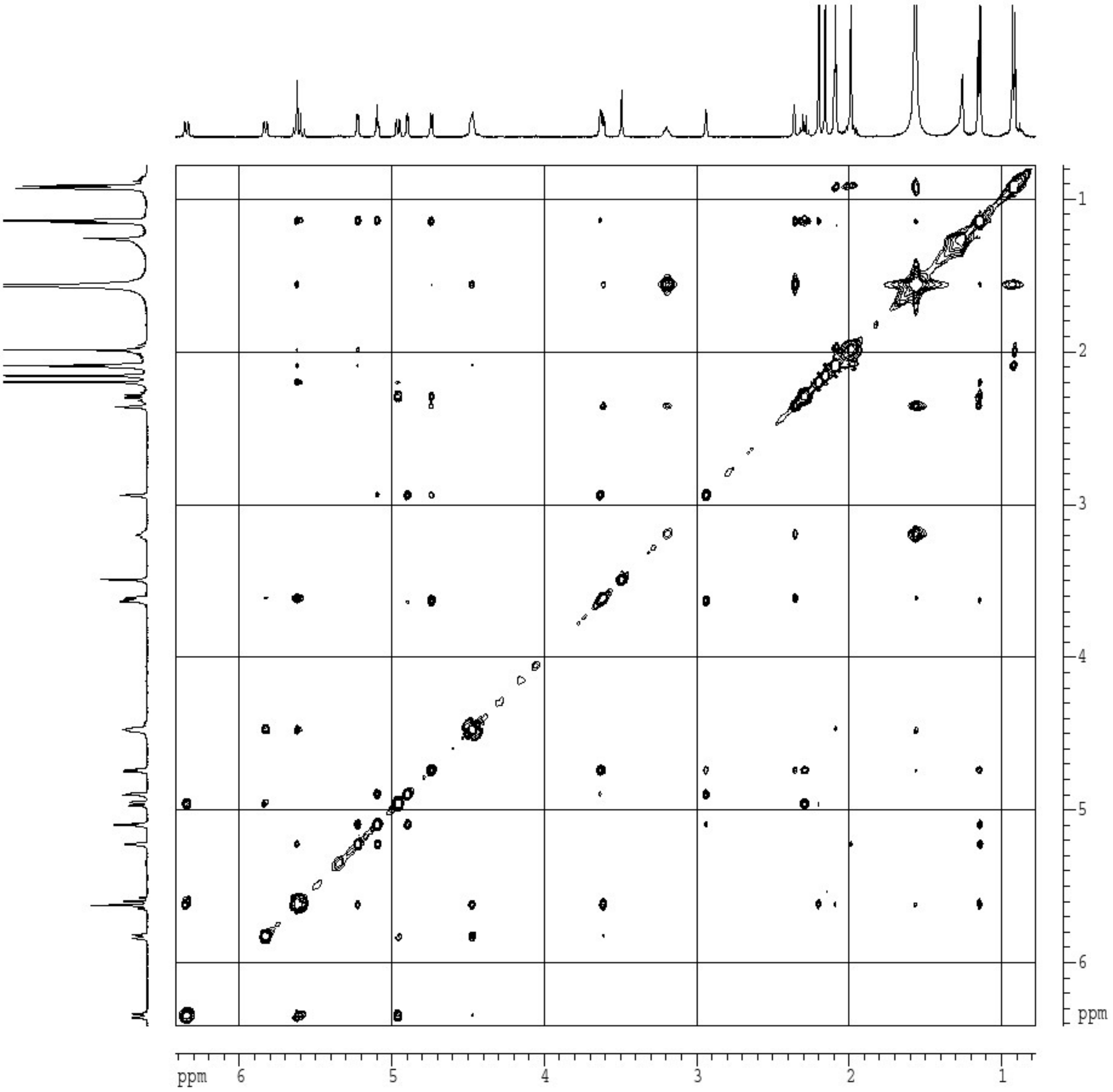

Figure S16. HR-ESIMS spectrum of the new compound **3**.

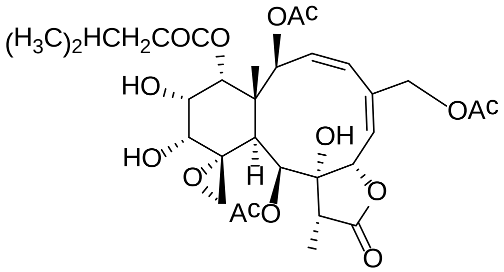

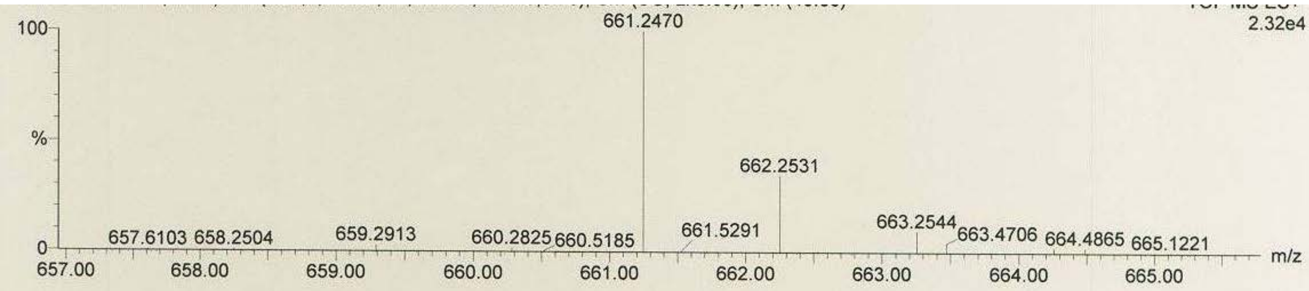

Minimum:	42.00				-1.5		
Maximum:	100.00		5.0	10.0	50.0		
Mass	RA	Calc. Mass	mDa	PPM	DBE	i-FIT	Formula
661.2470	100.00	661.2472	-0.2	-0.3	10.5	5.4	C31 H42 O14 Na

Figure S17. ^{1}H NMR spectrum of the new compound **3**.

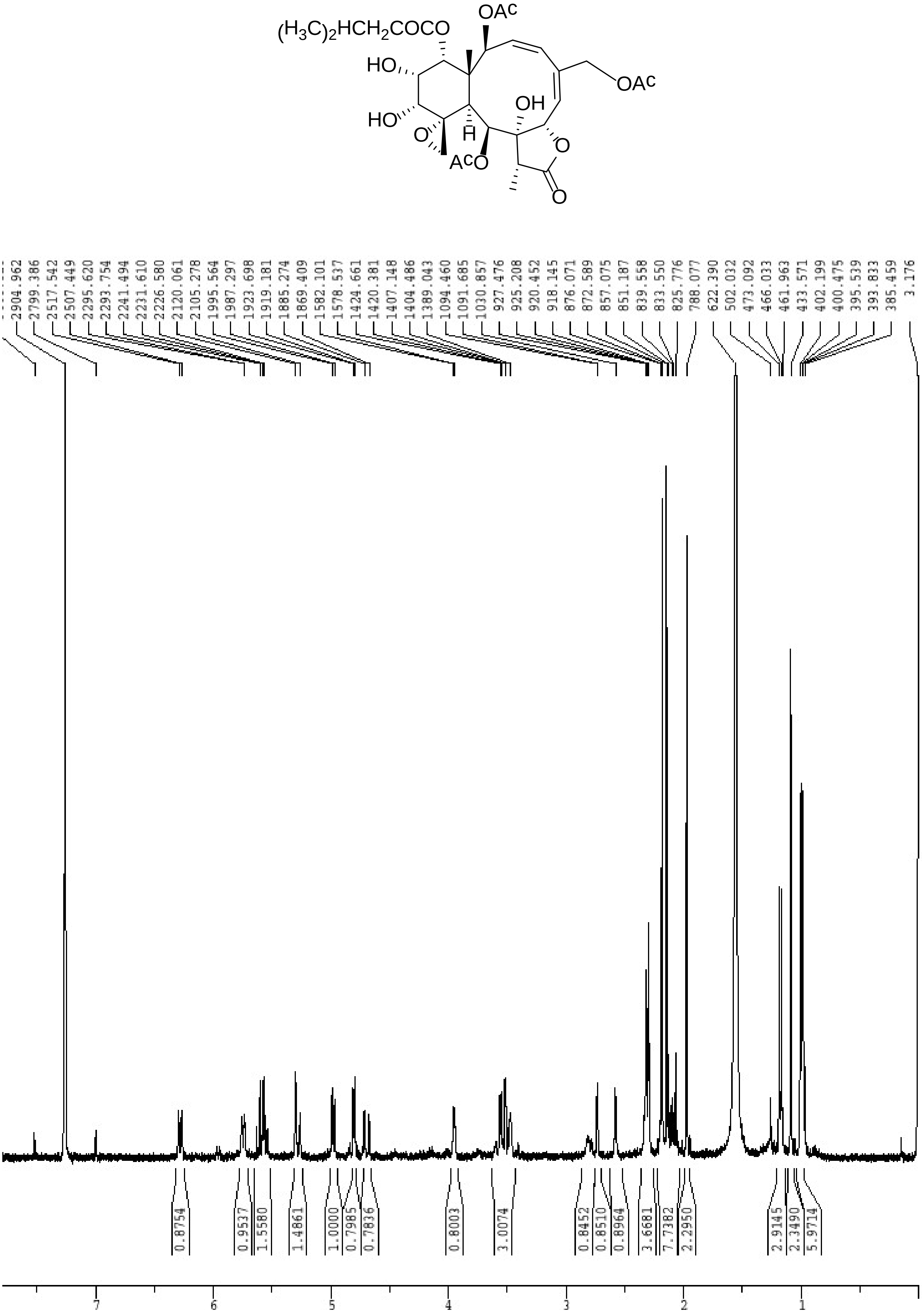

Figure S18. ^{13}C NMR spectrum of the new compound **3**.

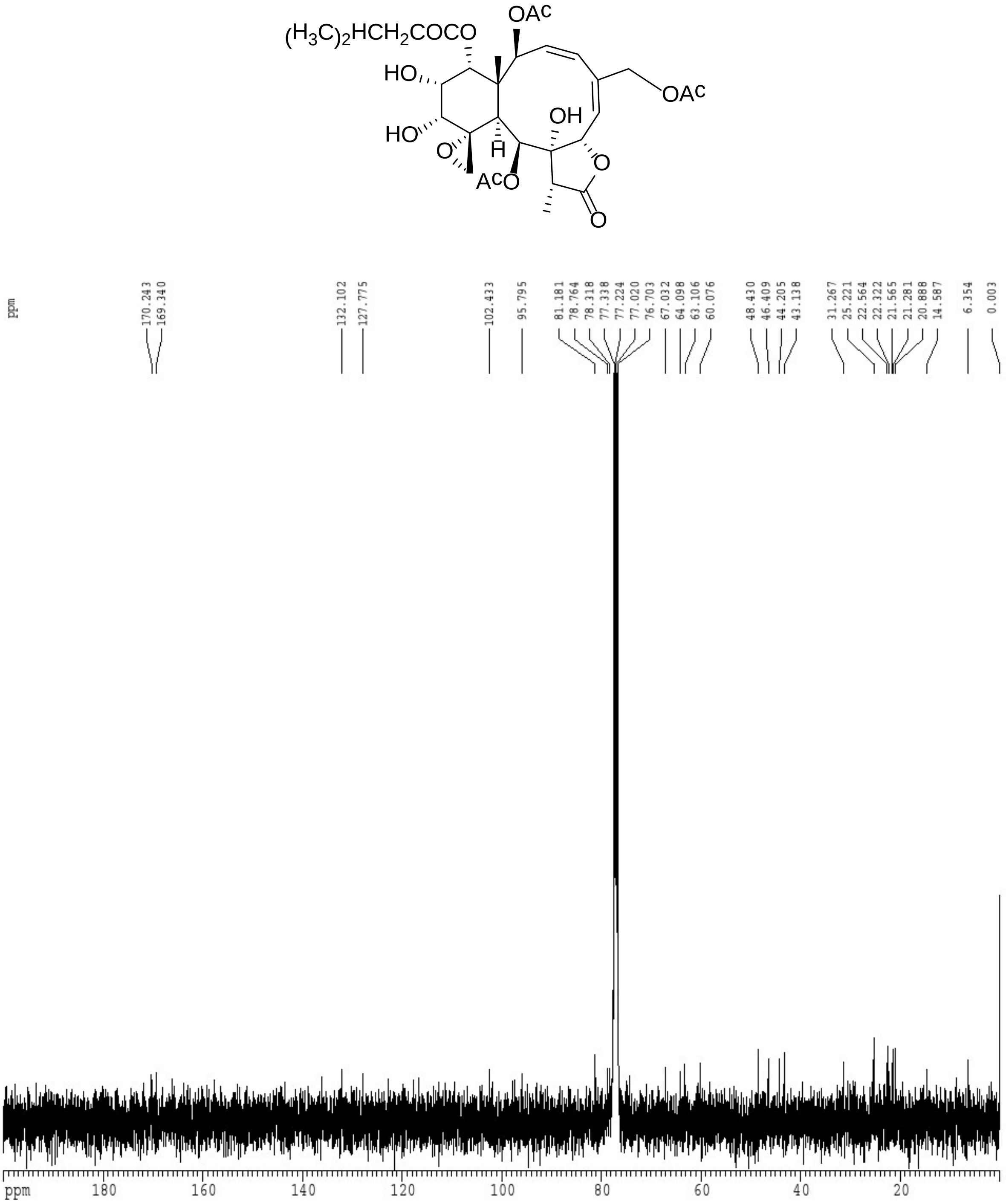

Figure S19. DEPT spectrum of the new compound **3**.

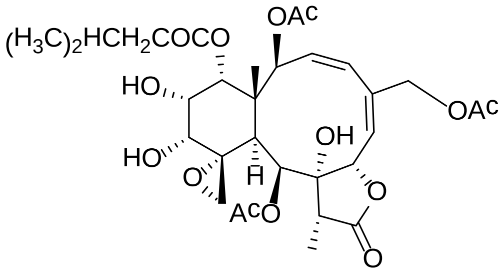

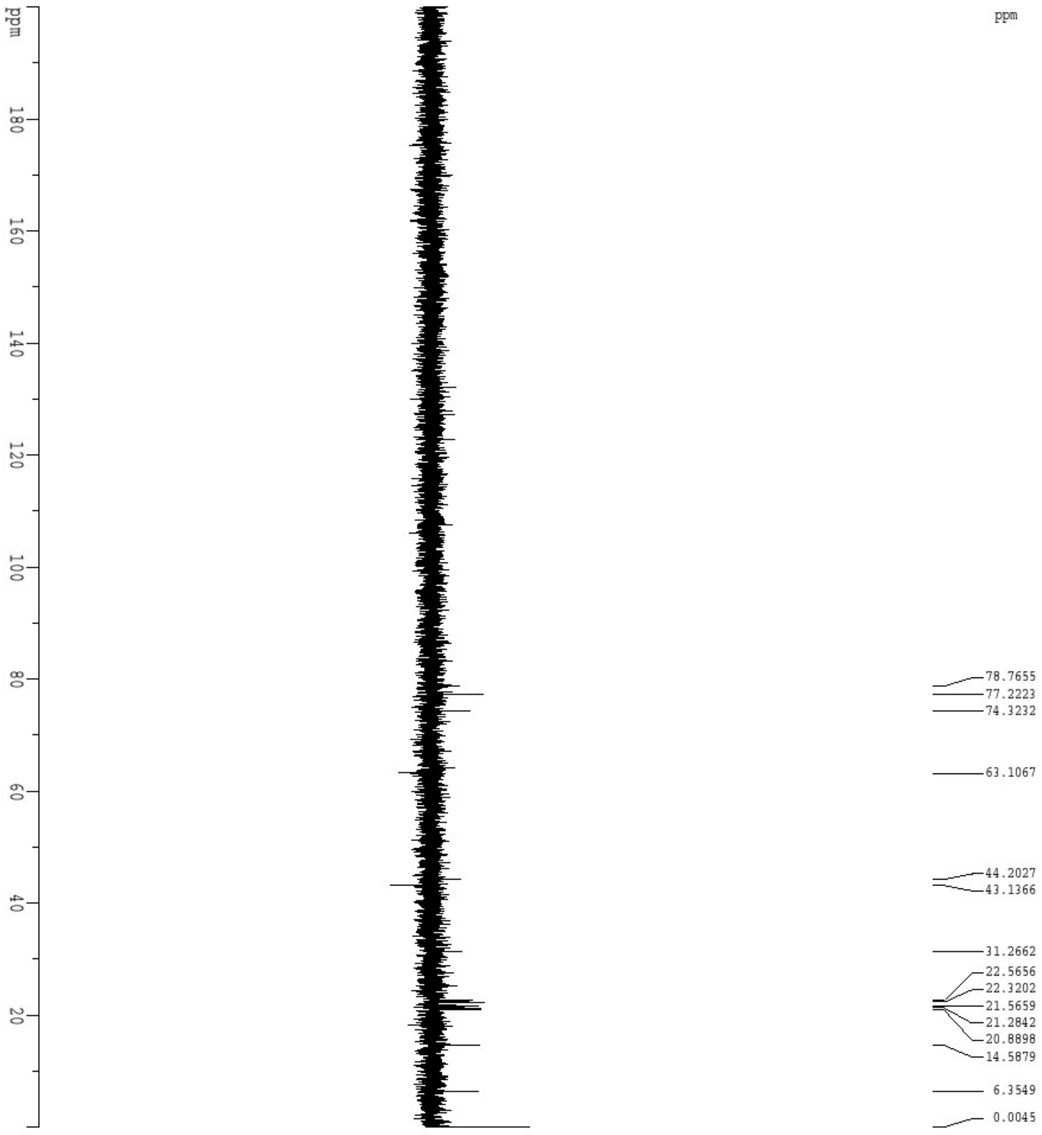

Figure S20. HSQC spectrum of the new compound **3**.

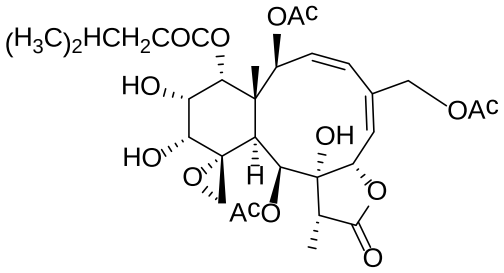

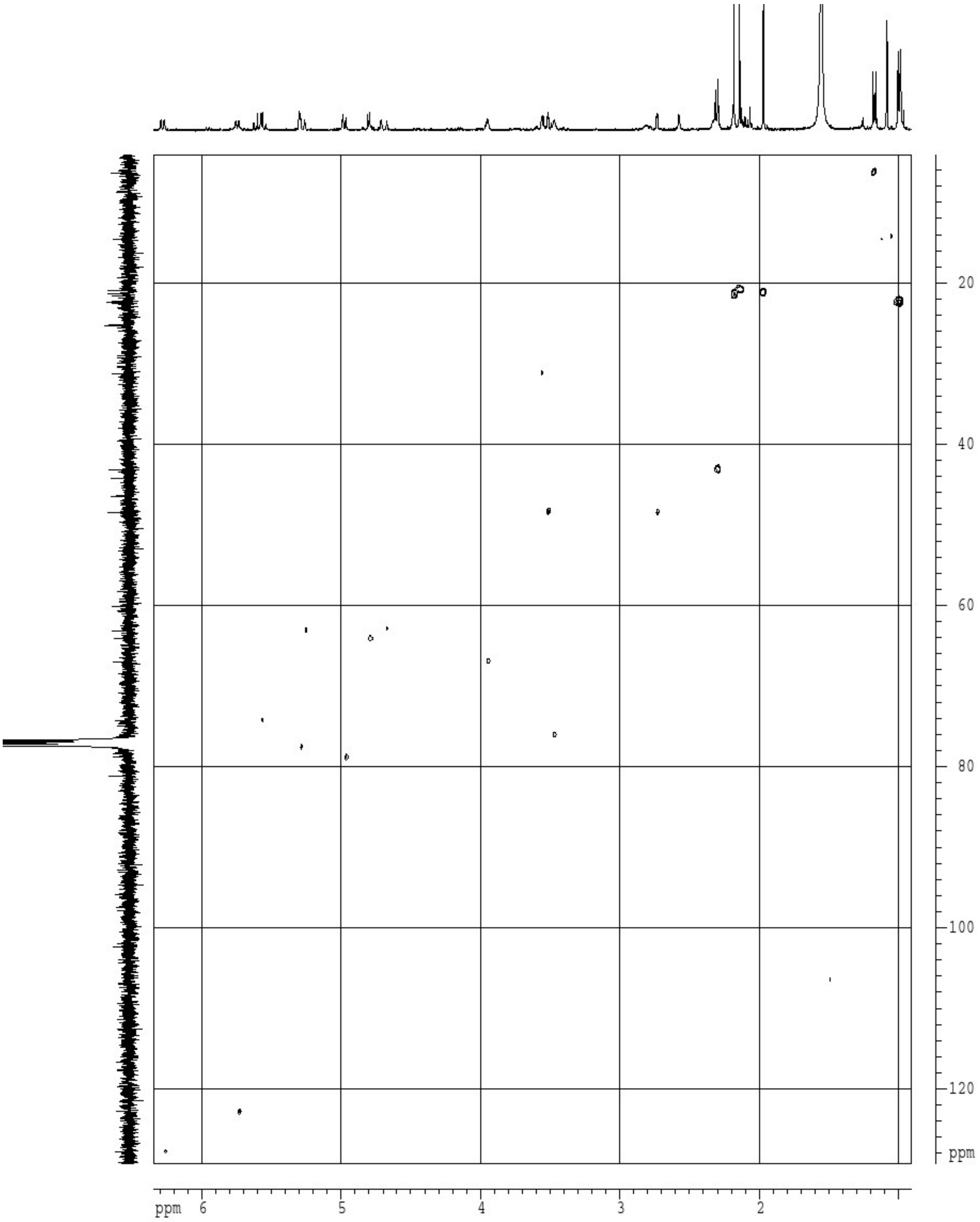

Figure S21. ^{1}H-^{1}H COSY spectrum of the new compound **3**.

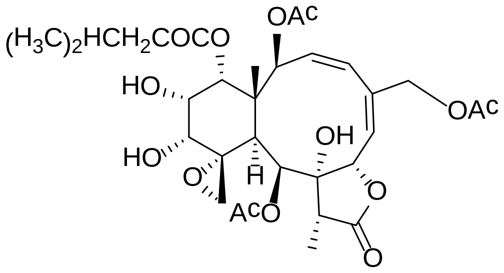

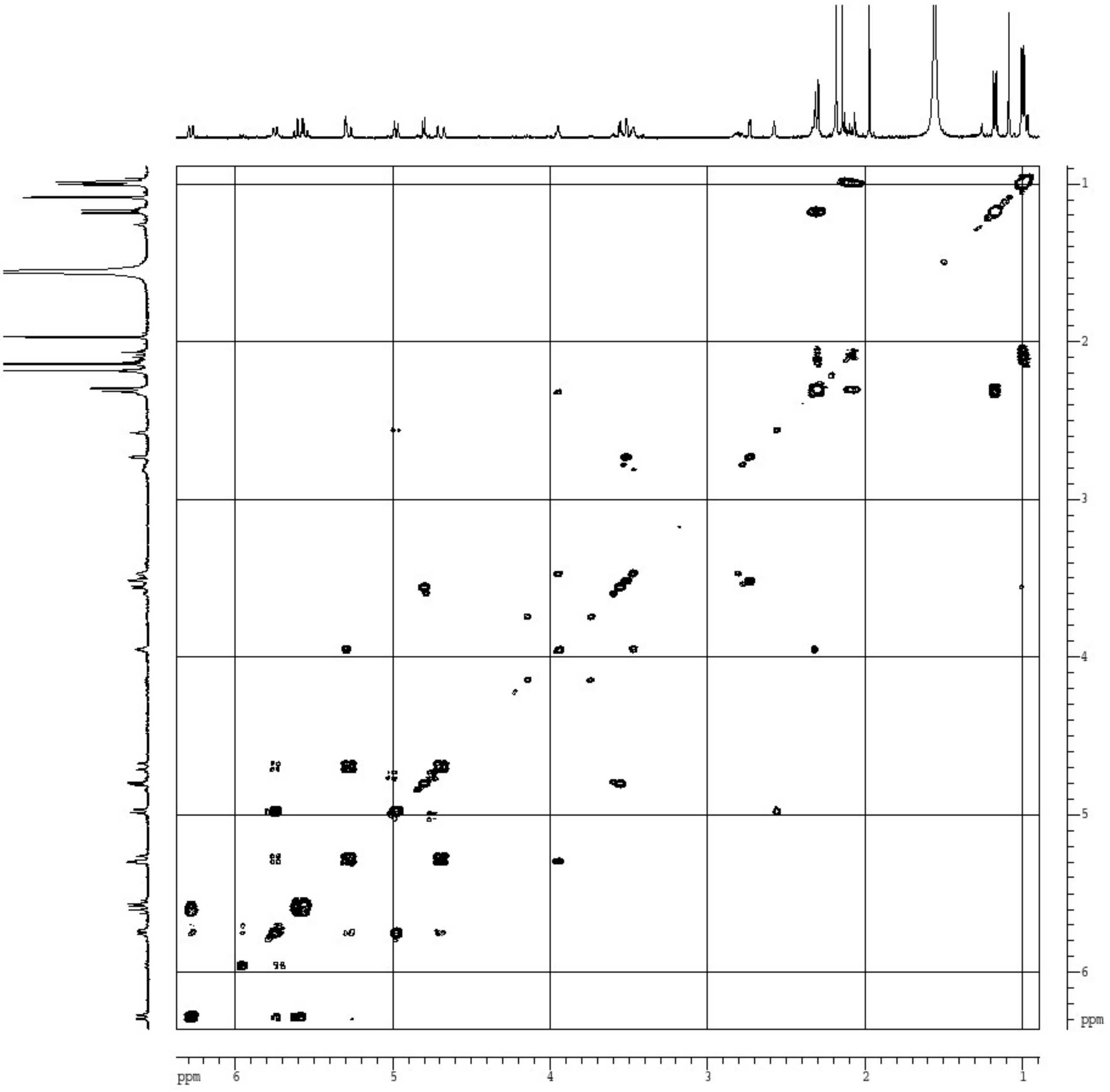

Figure S22. HMBC spectrum of the new compound **3**.

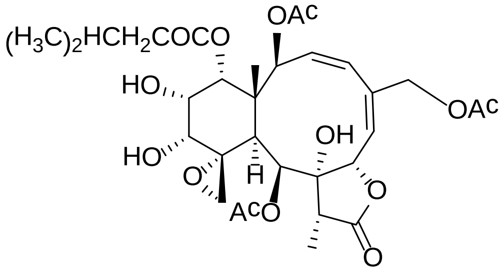

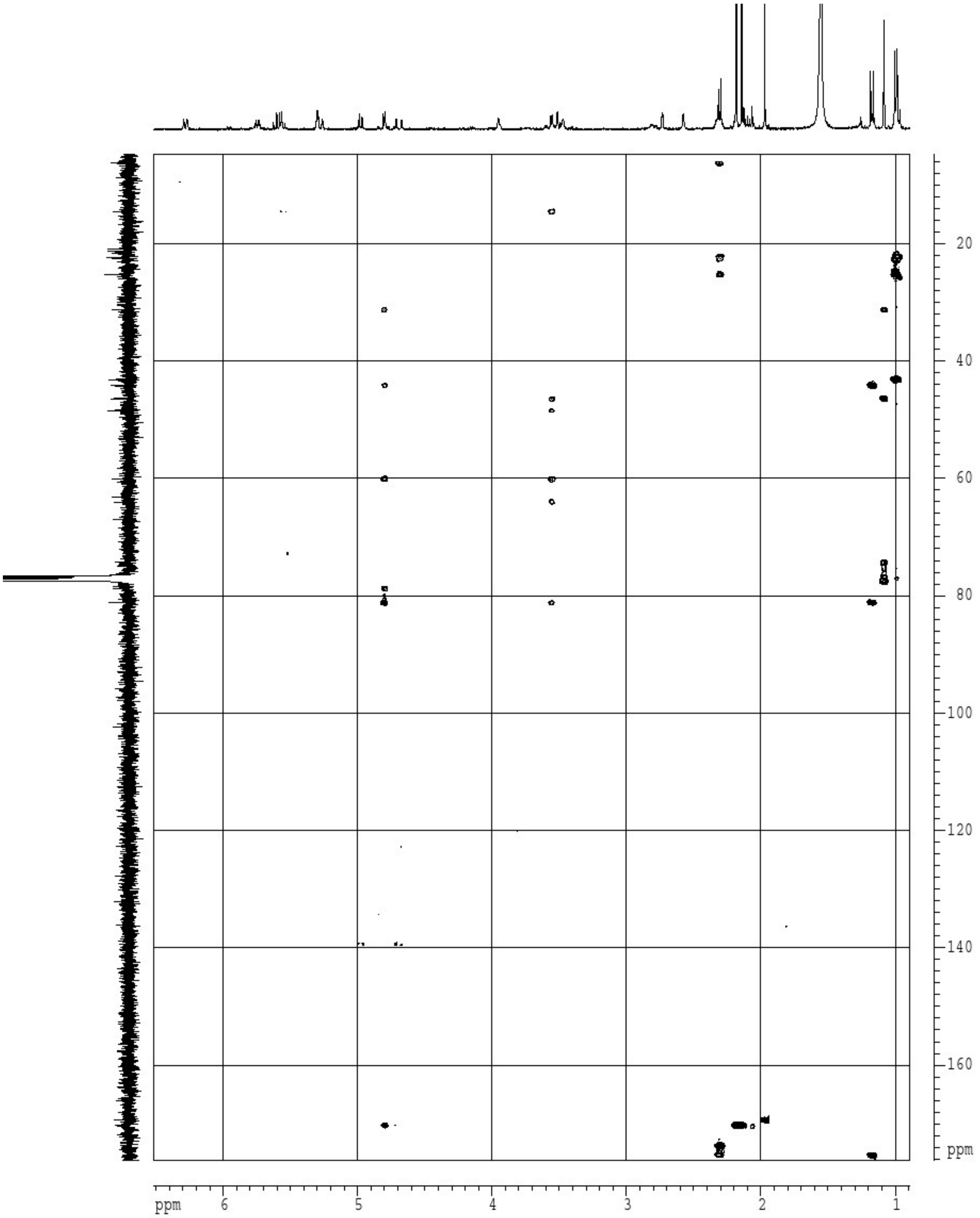

Figure S23. NOESY spectrum of the new compound **3**.

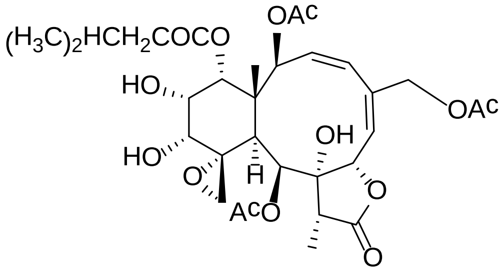

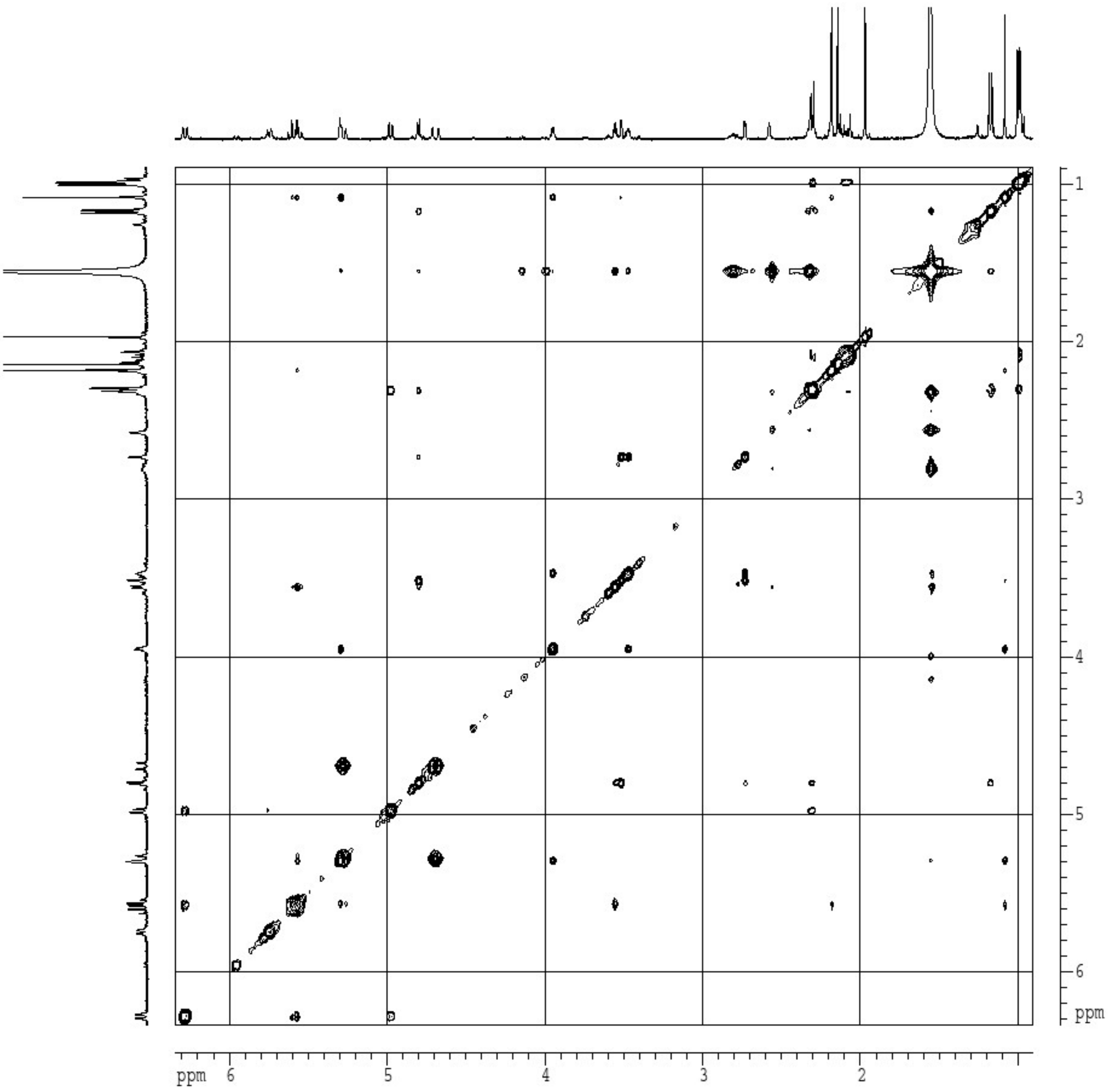

Figure S24. HR-ESIMS spectrum of the new compound **4**.

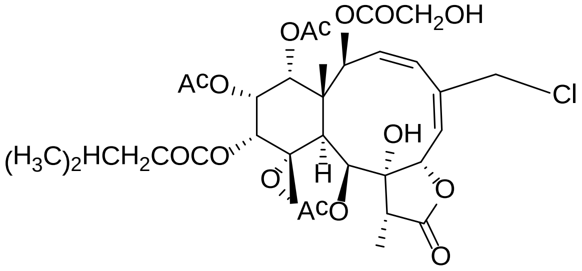

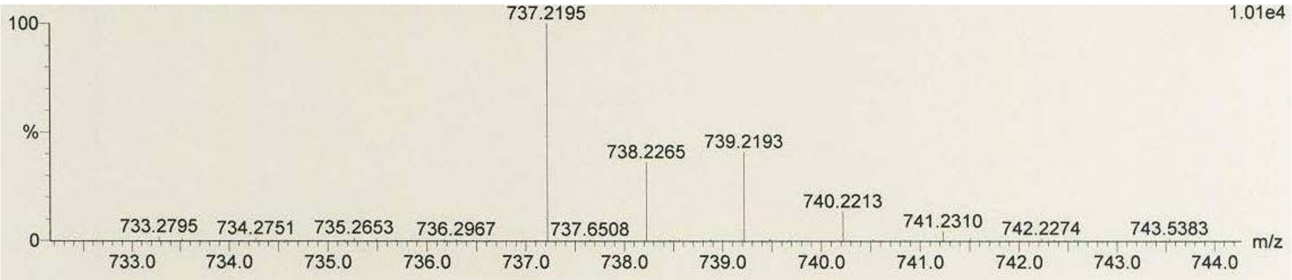

Minimum:	37.00				-1.5		
Maximum:	100.00		5.0	7.0	50.0		
Mass	RA	Calc. Mass	mDa	PPM	DBE	i-FIT	Formula
737.2195	100.00	737.2188	0.7	0.9	11.5	3.8	C33 H43 O15 Na 35Cl
739.2193	40.91	739.2159	3.4	4.6	11.5	9.5	C33 H43 O15 Na 37Cl

Figure S25. ^{1}H NMR spectrum of the new compound **4**.

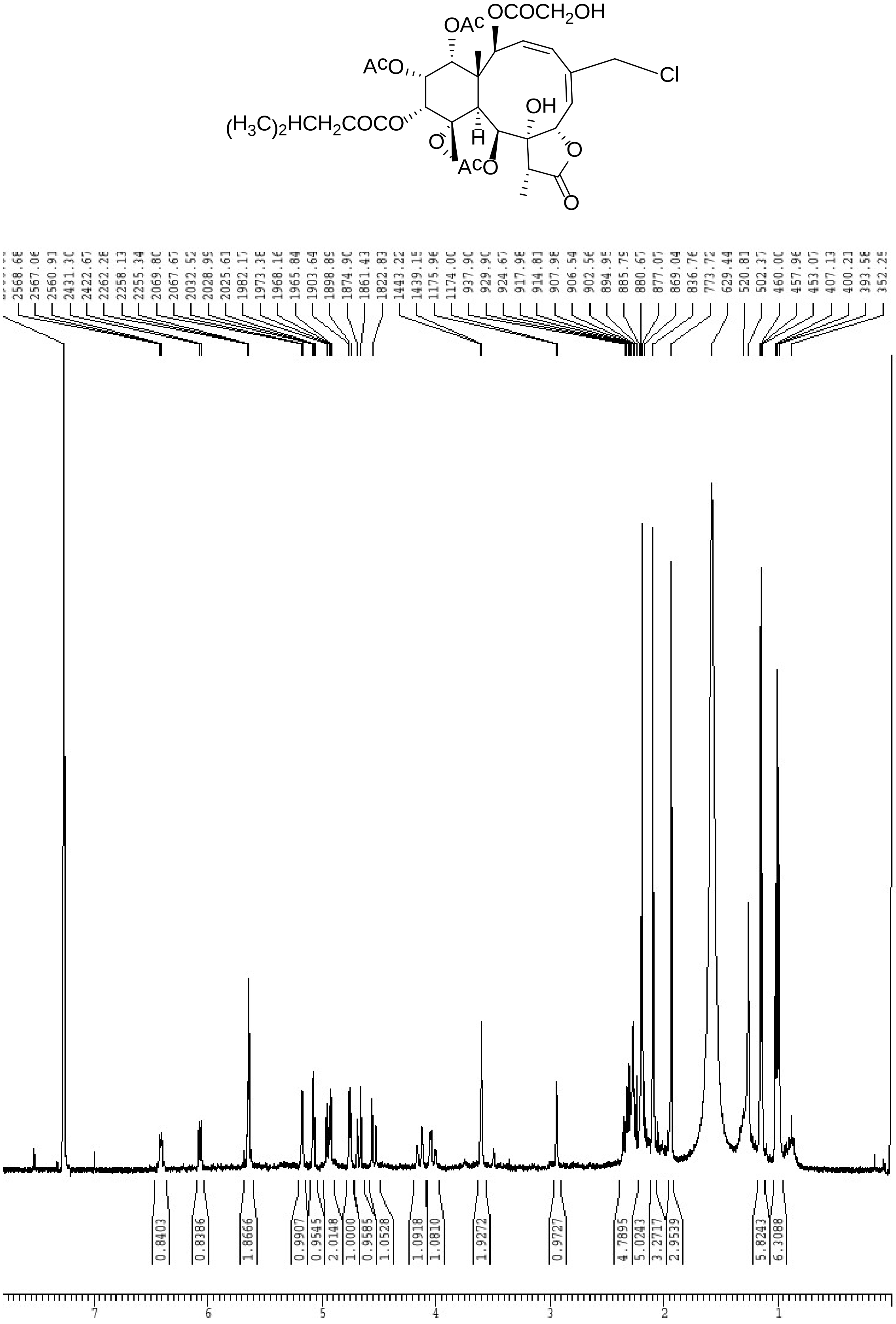

Figure S26. ^{13}C NMR spectrum of the new compound **4**.

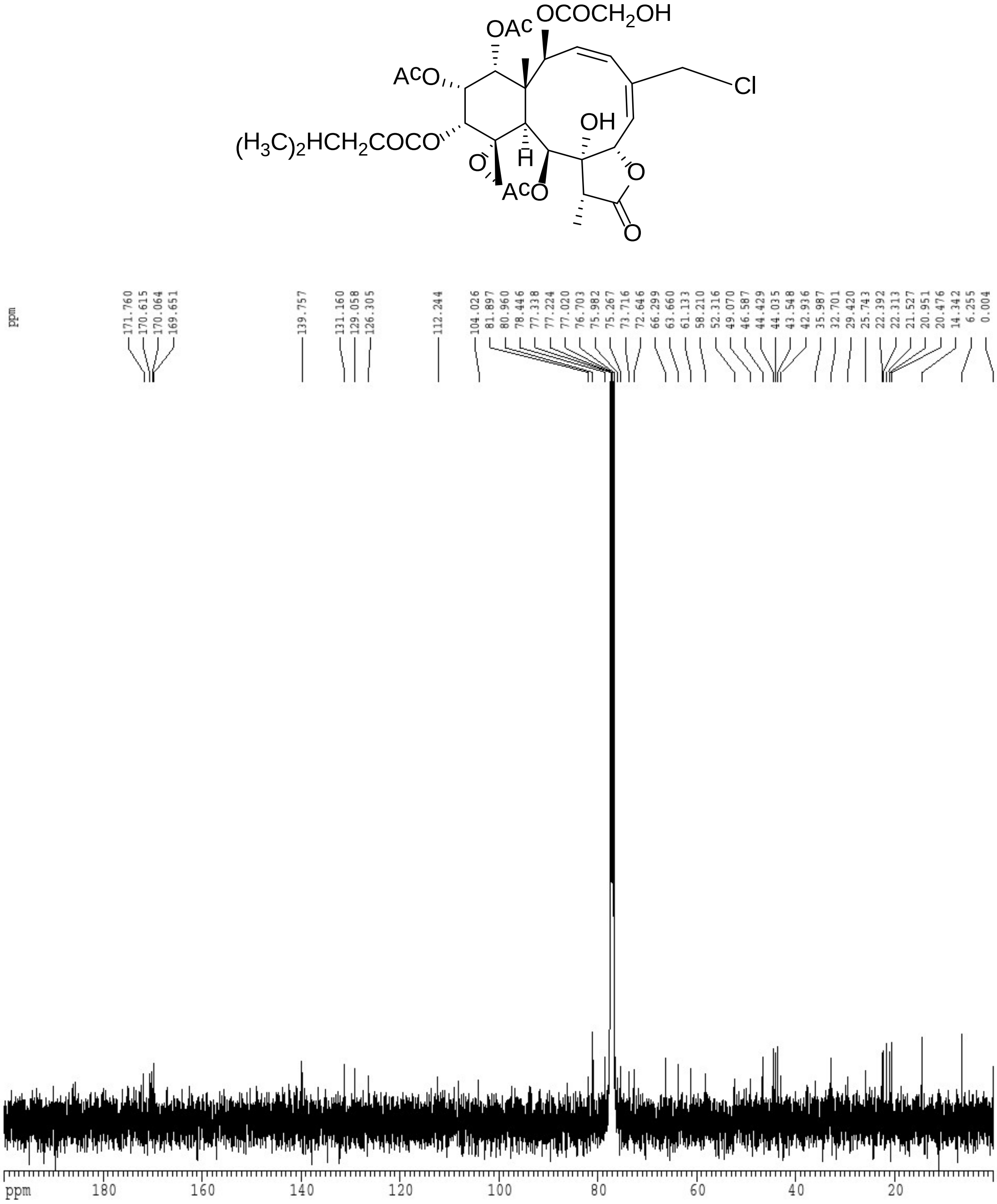

Figure S27. DEPT spectrum of the new compound **4**.

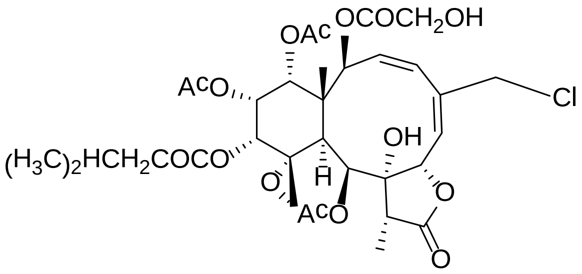

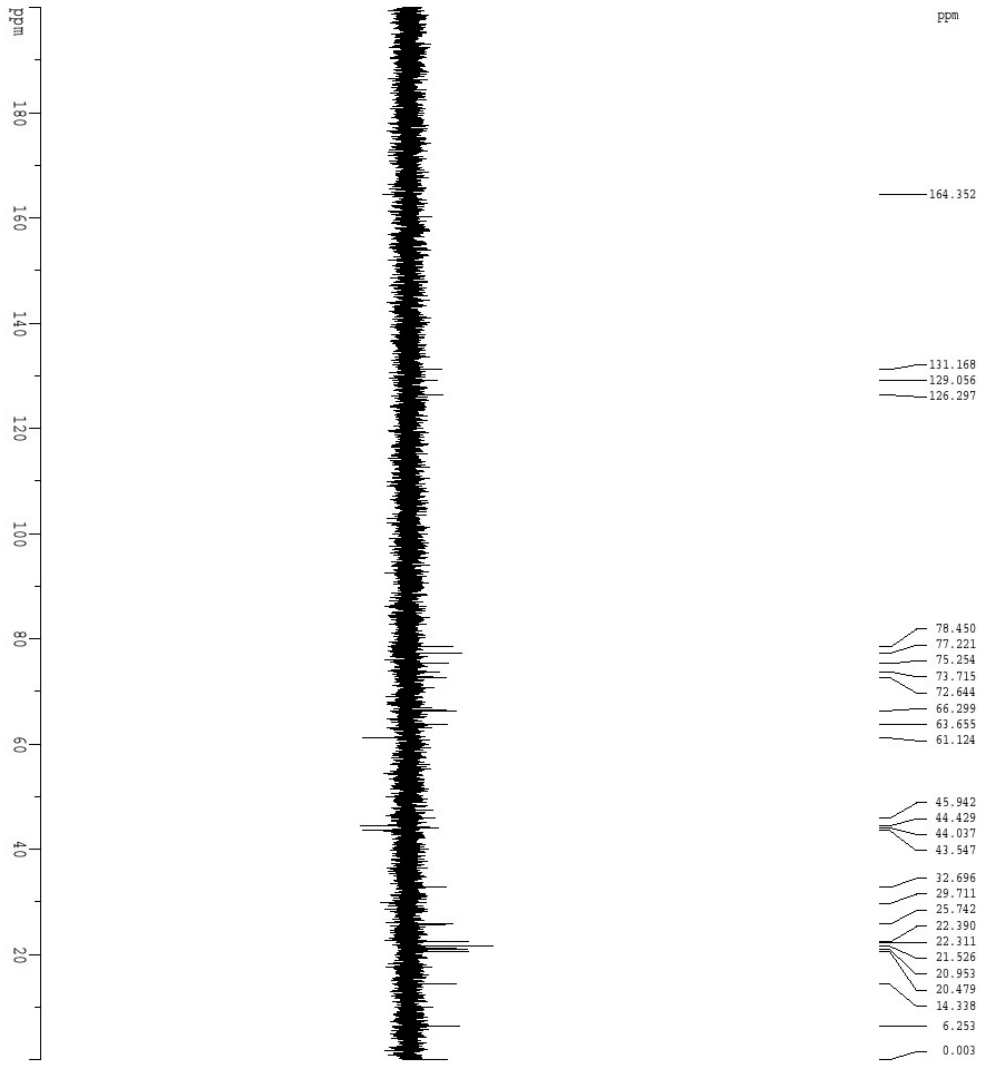

Figure S28. HSQC spectrum of the new compound **4**.

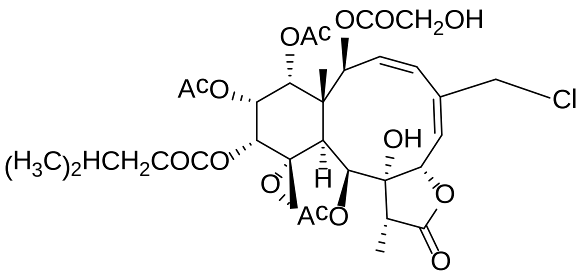

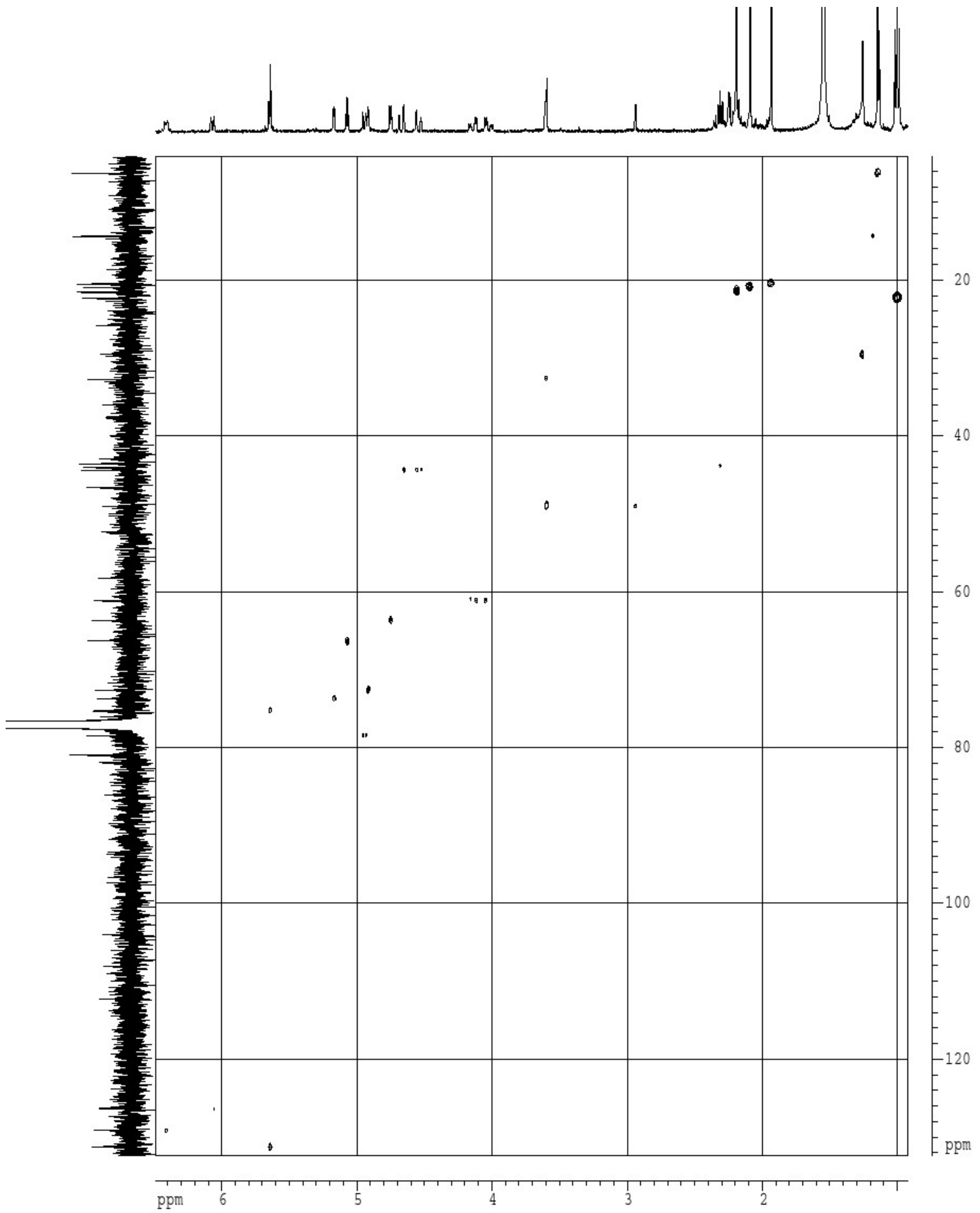

Figure S29. ^{1}H-^{1}H COSY spectrum of the new compound **4**.

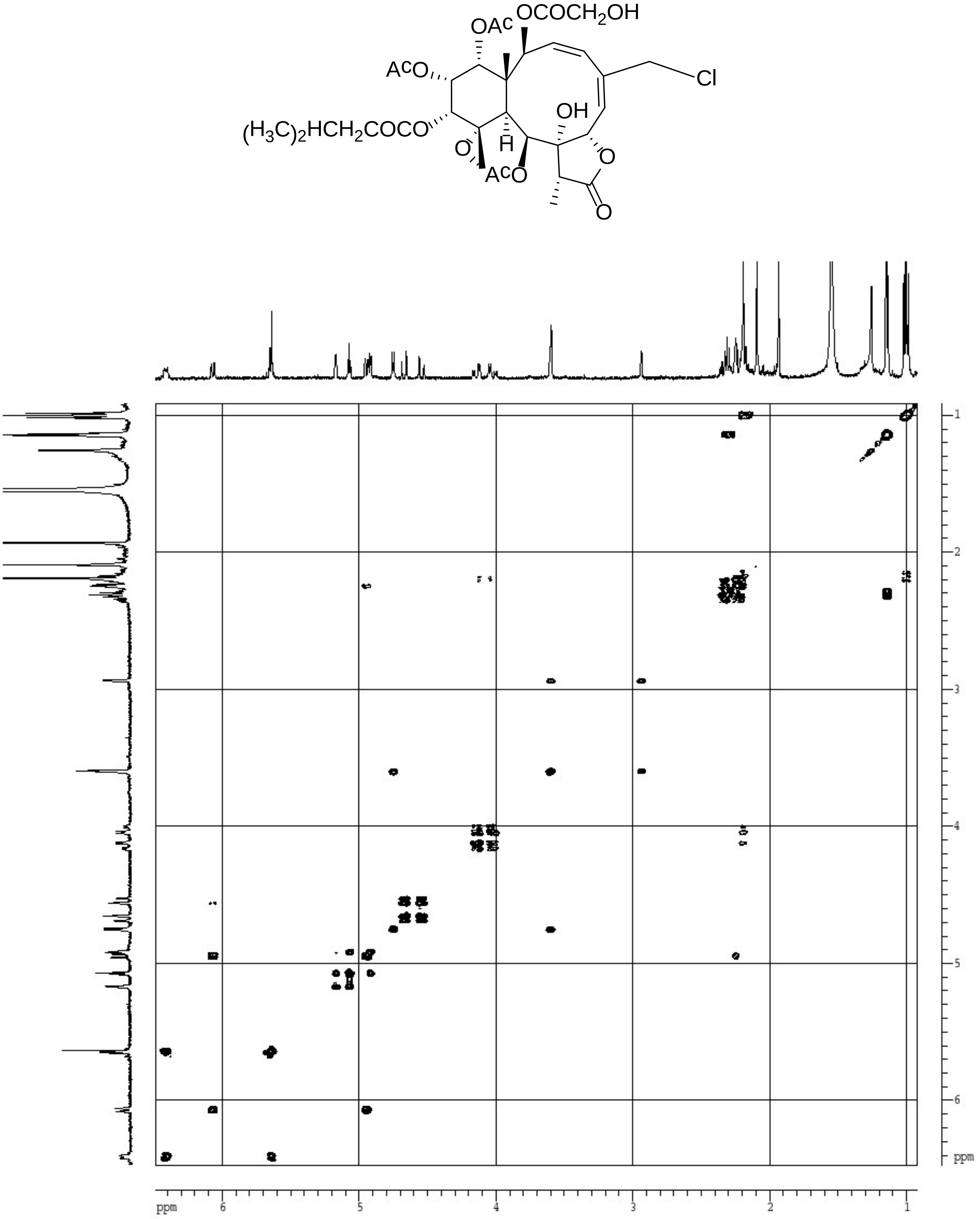

Figure S30. HMBC spectrum of the new compound **4**.

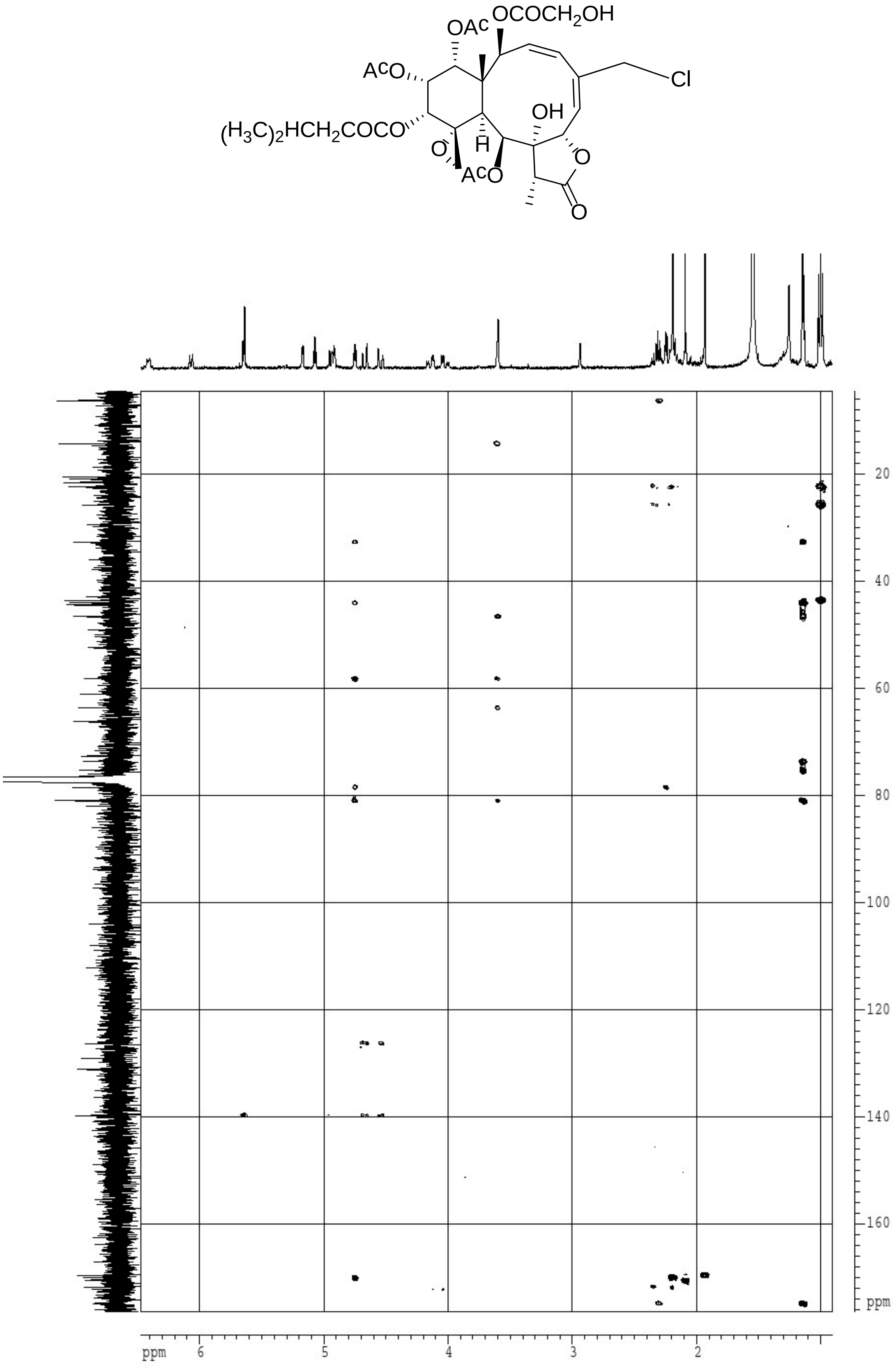

Figure S31. NOESY spectrum of the new compound **4**.

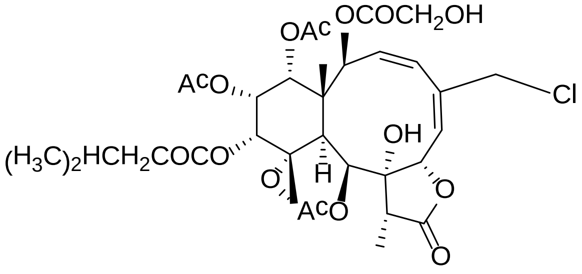

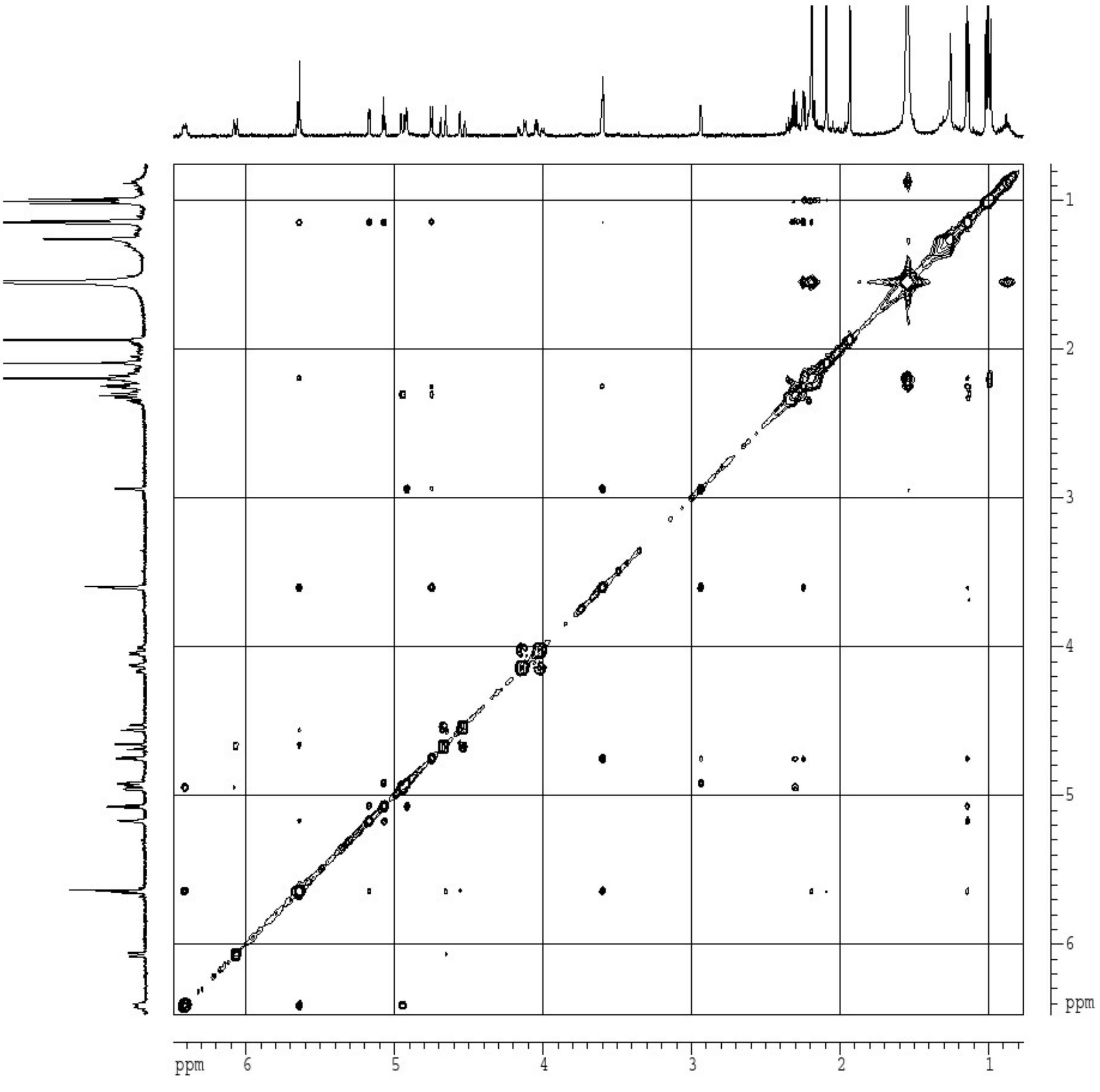

Figure S32. HR-ESIMS spectrum of the new compound **5**.

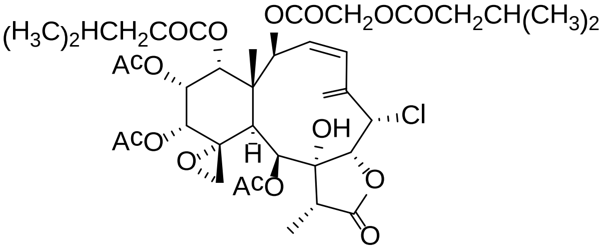

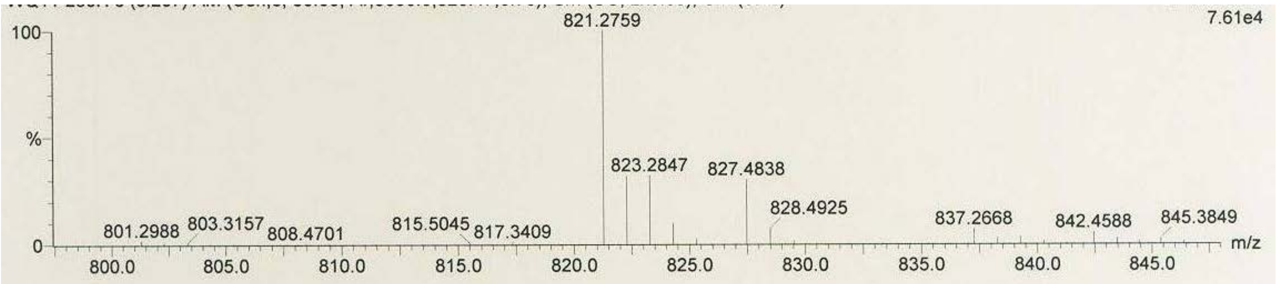

Minimum:	35.00				-1.5		
Maximum:	100.00		5.0	10.0	50.0		
Mass	RA	Calc. Mass	mDa	PPM	DBE	i-FIT	Formula
821.2759	100.00	821.2763	-0.4	-0.5	12.5	1572.7	C38 H51 O16 Na Cl

Figure S33. ^{1}H NMR spectrum of the new compound **5**.

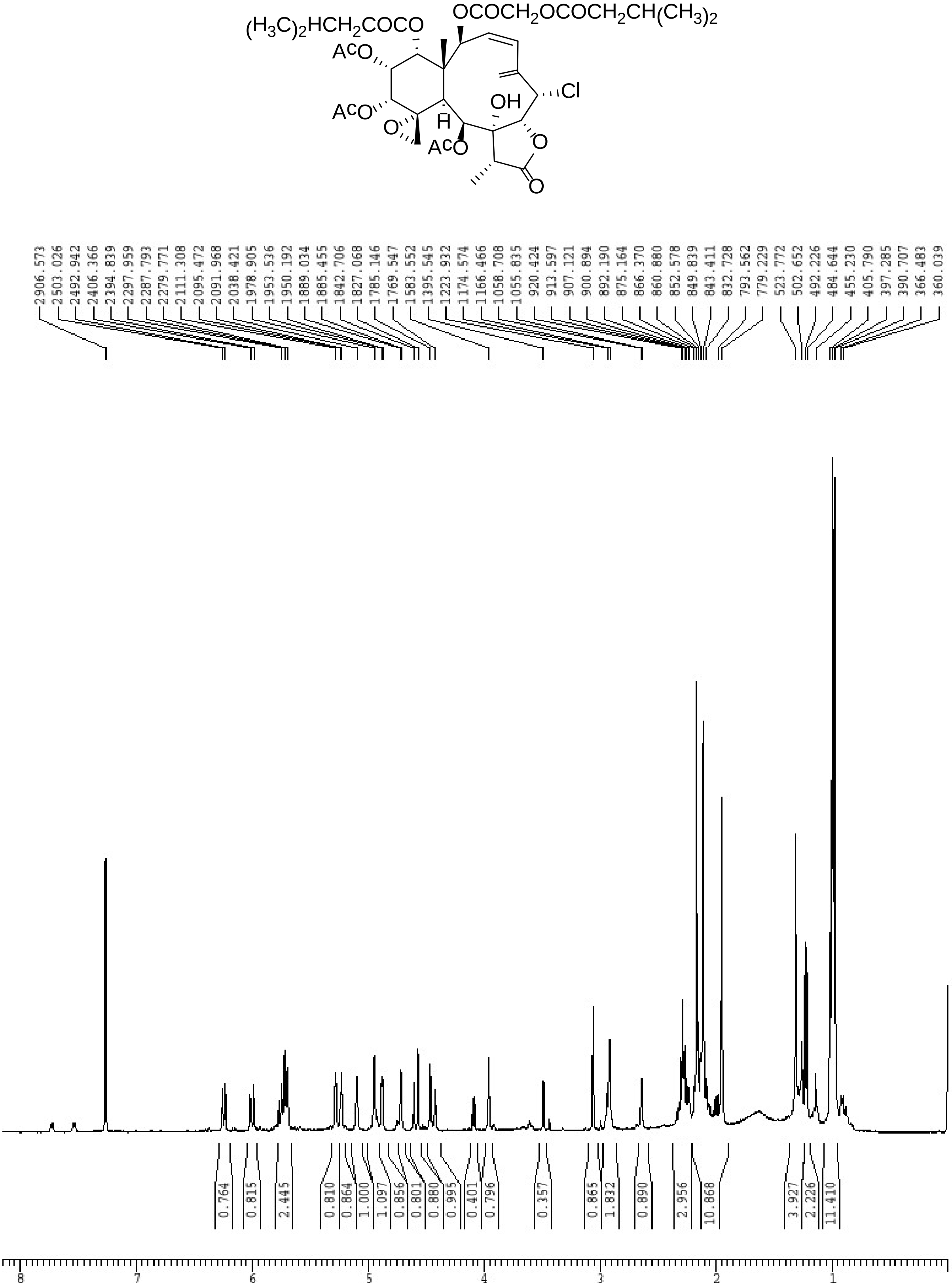

Figure S34. ^{13}C NMR spectrum of the new compound **5**.

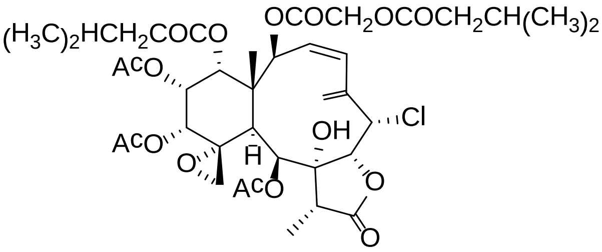

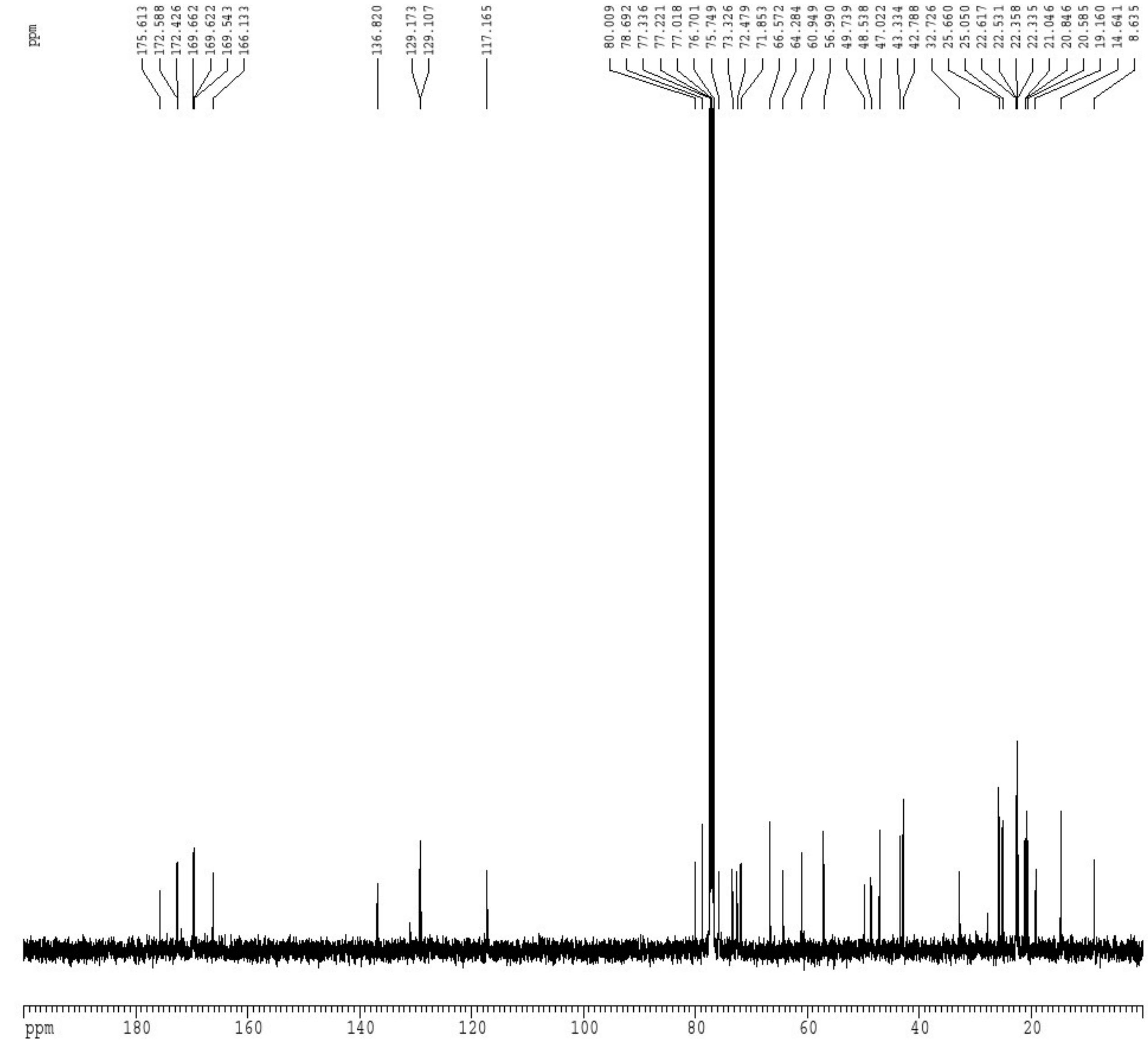

Figure S35. DEPT spectrum of the new compound **5**.

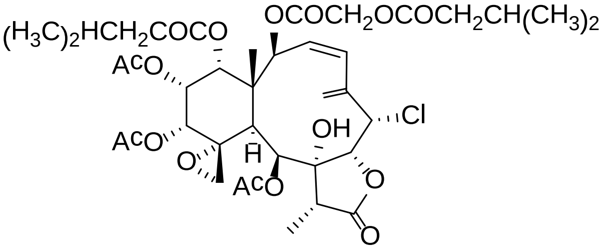

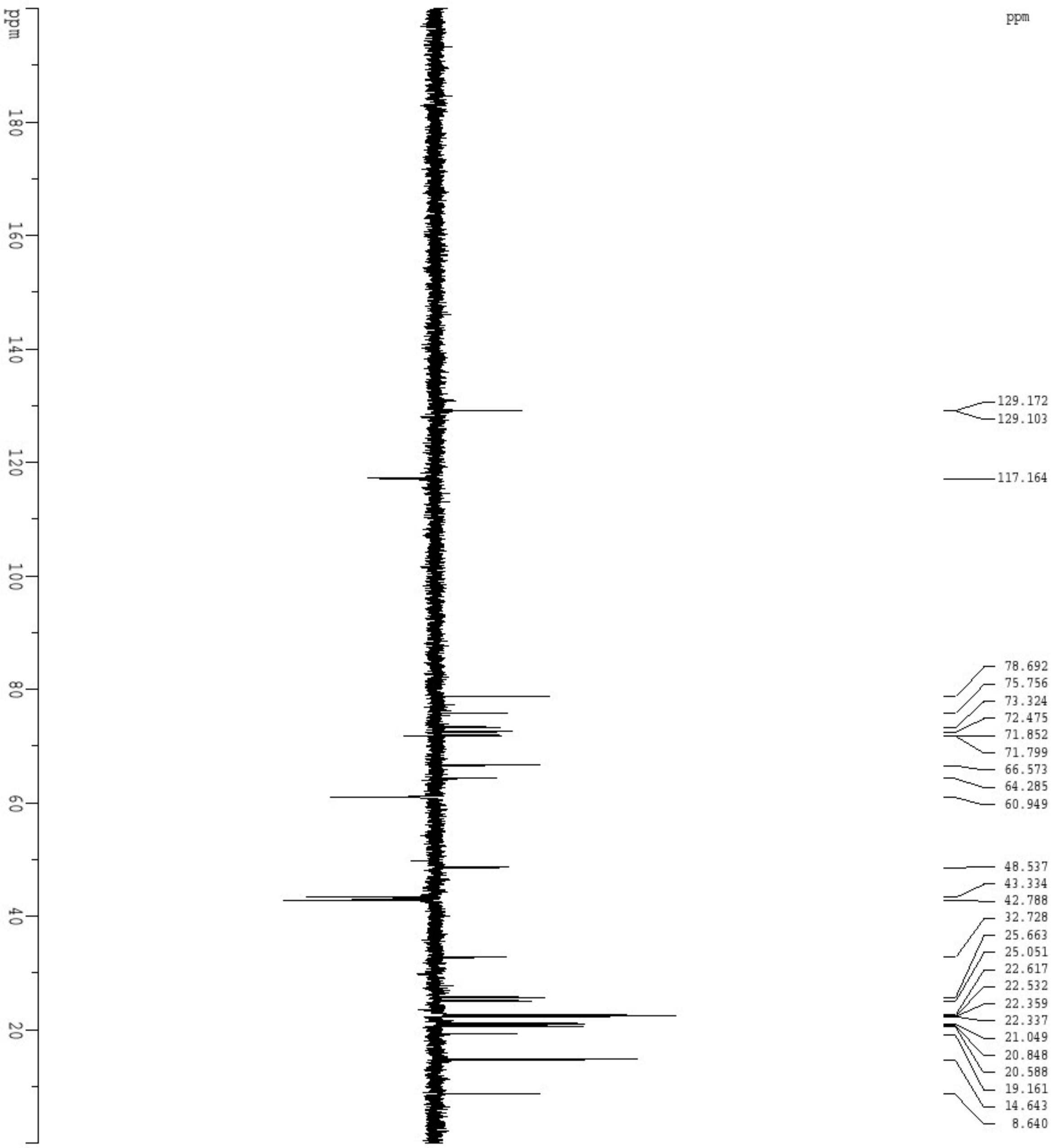

Figure S36. HSQC spectrum of the new compound **5**.

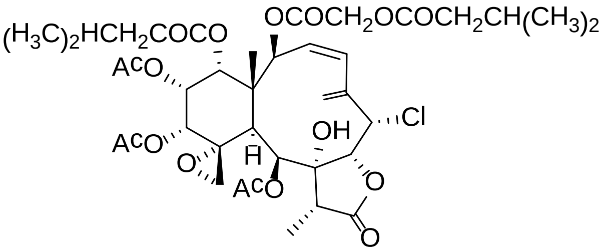

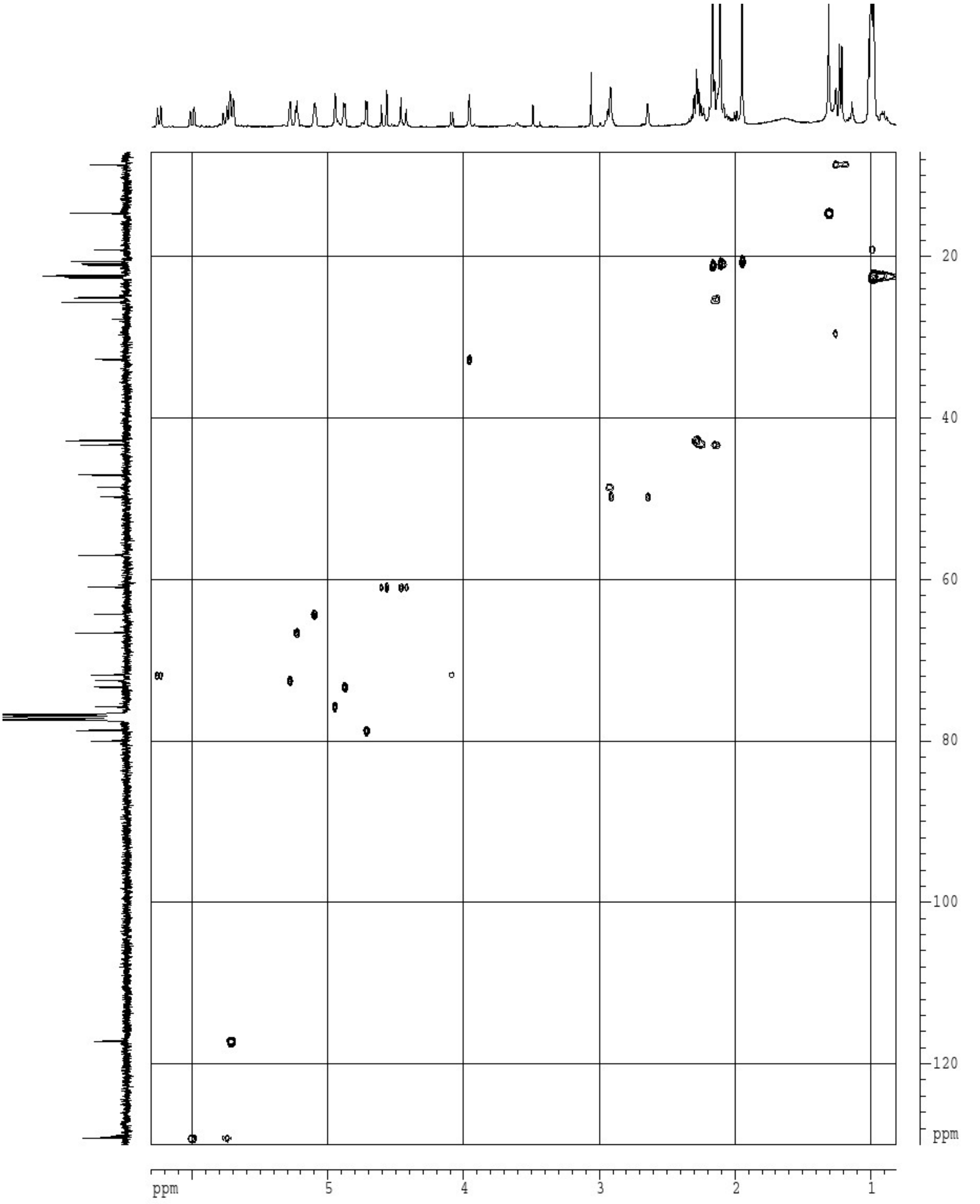

Figure S37. ^1H-^1H COSY spectrum of the new compound **5**.

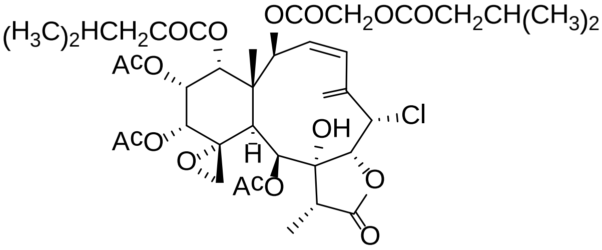

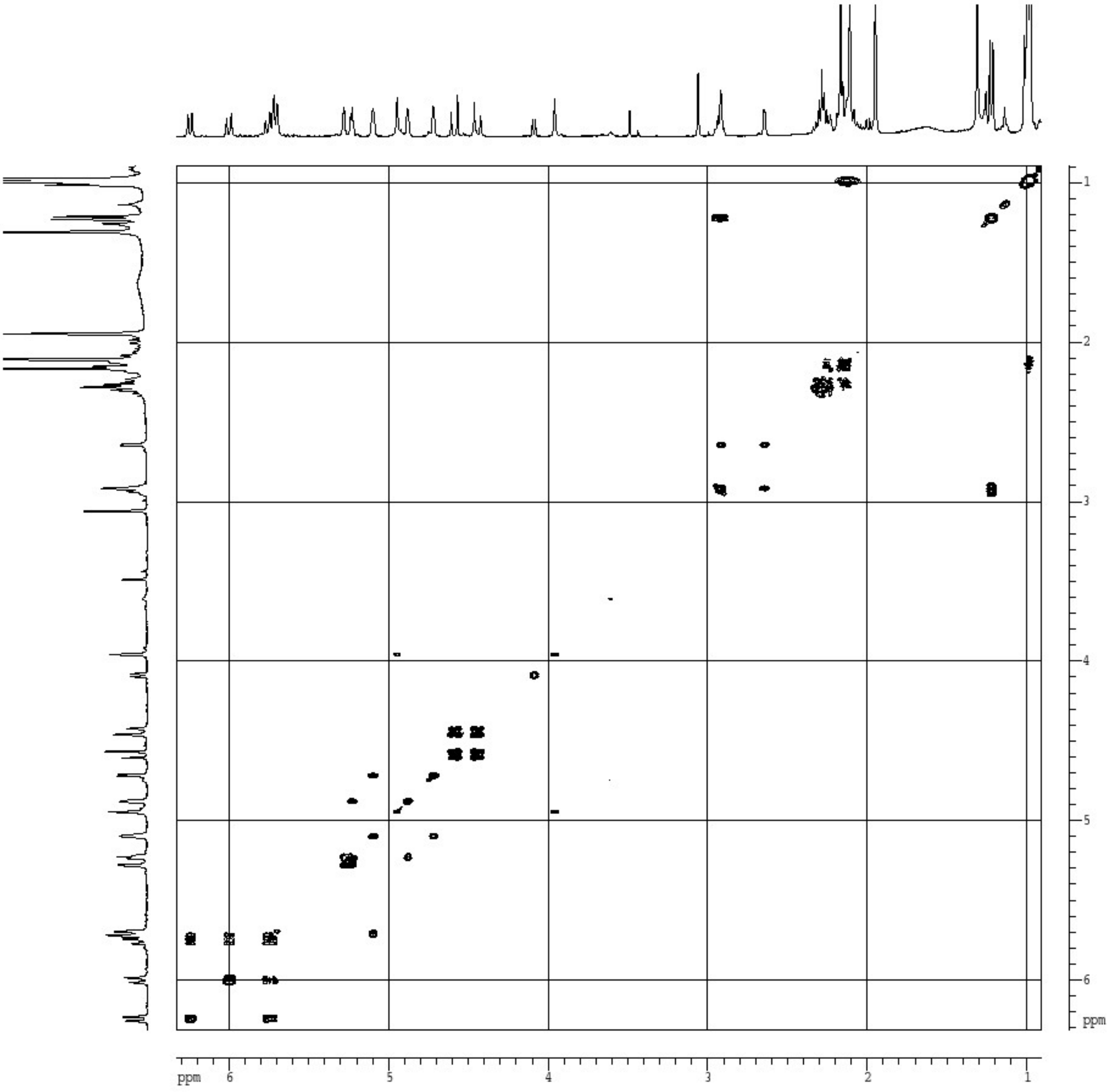

Figure S38. HMBC spectrum of the new compound **5**.

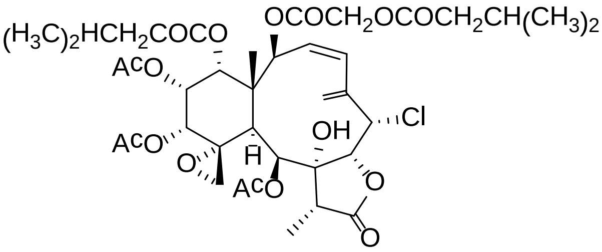

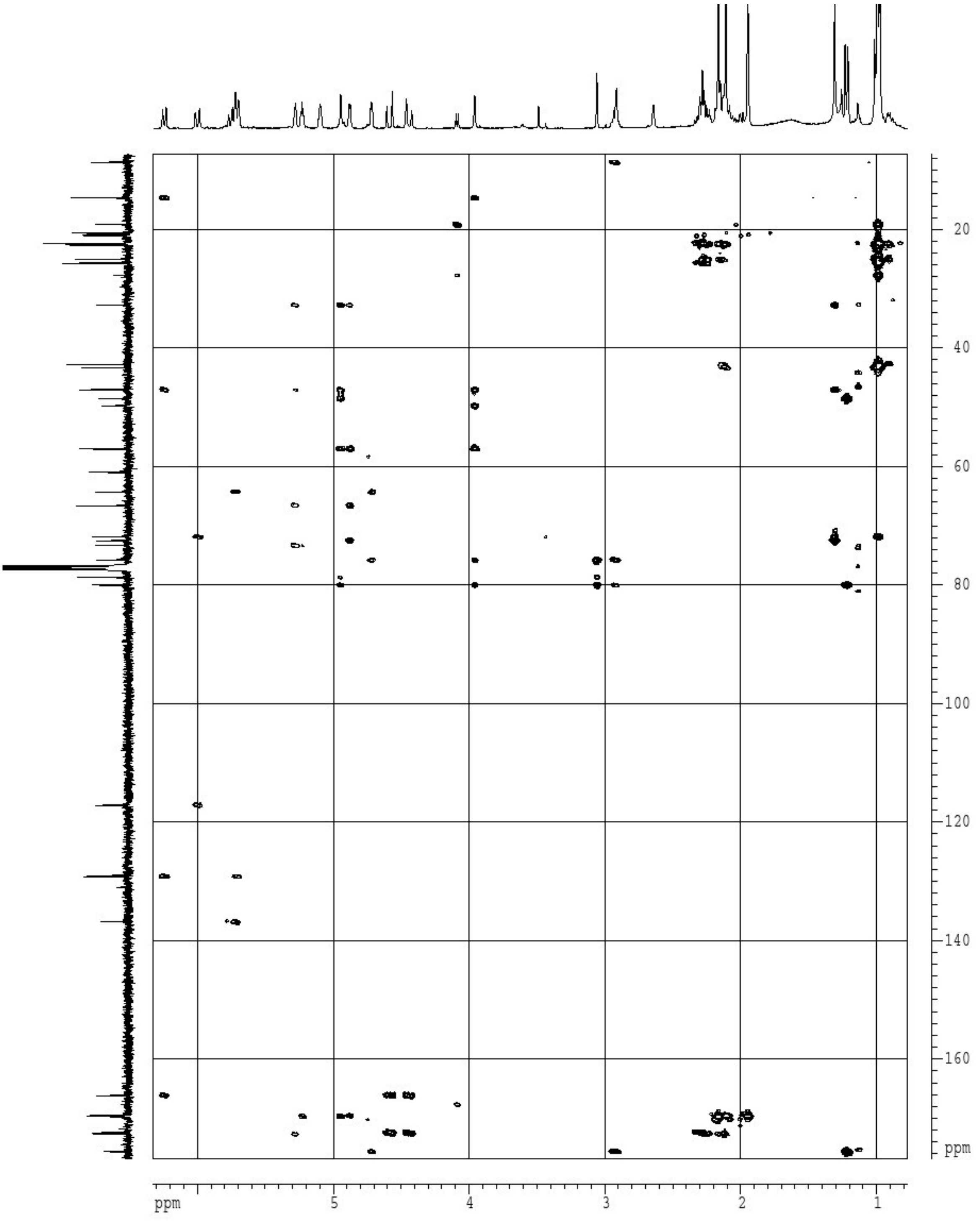

Figure S39. NOESY spectrum of the new compound **5**.

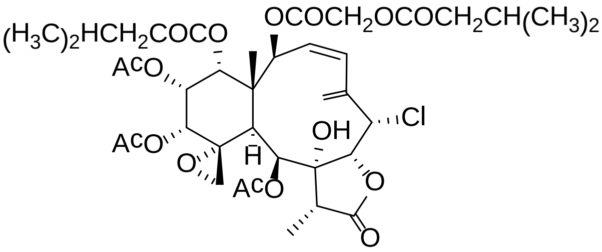

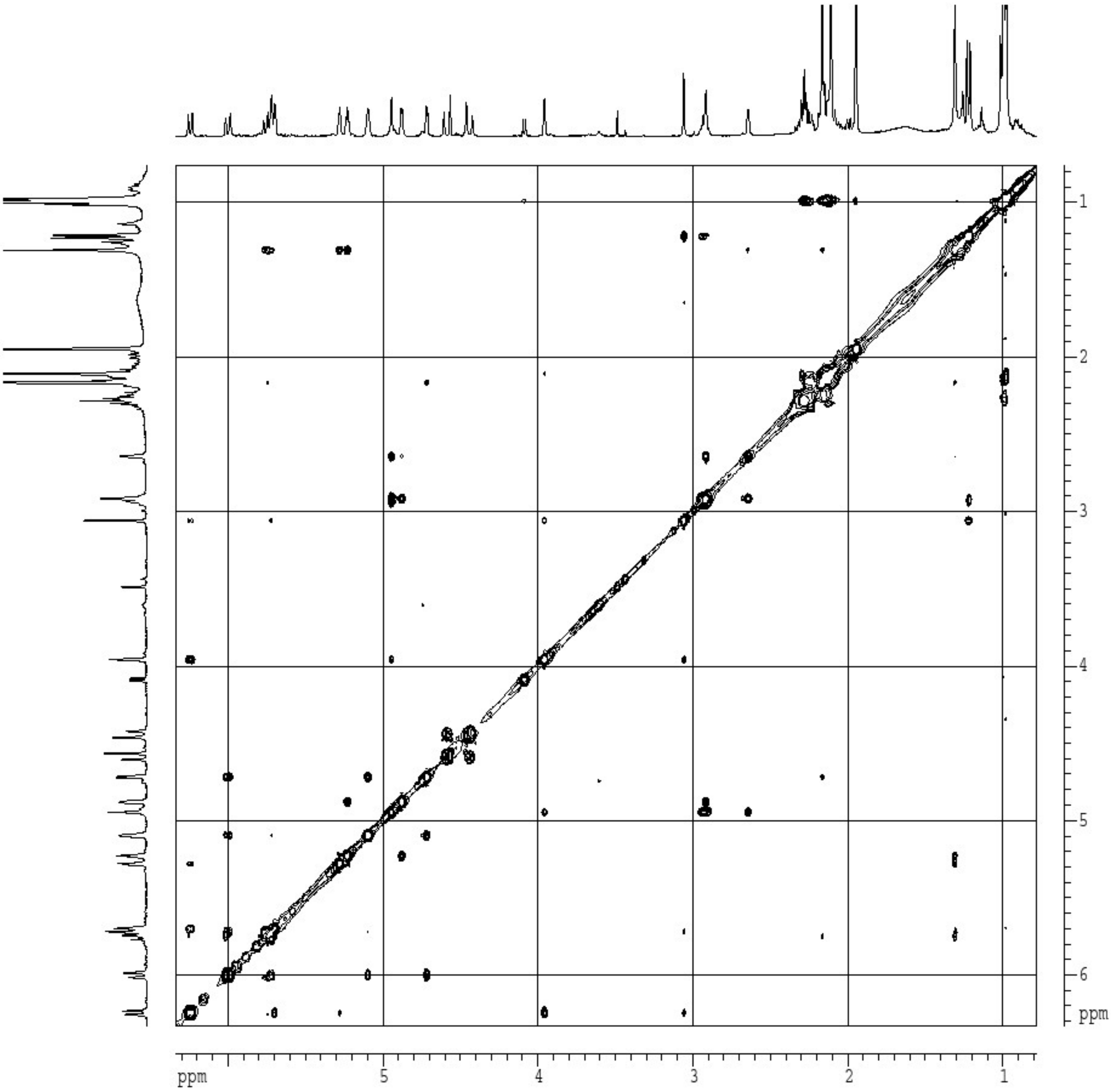

Figure S40. HR-ESIMS spectrum of the new compound **6**.

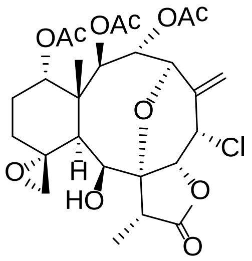

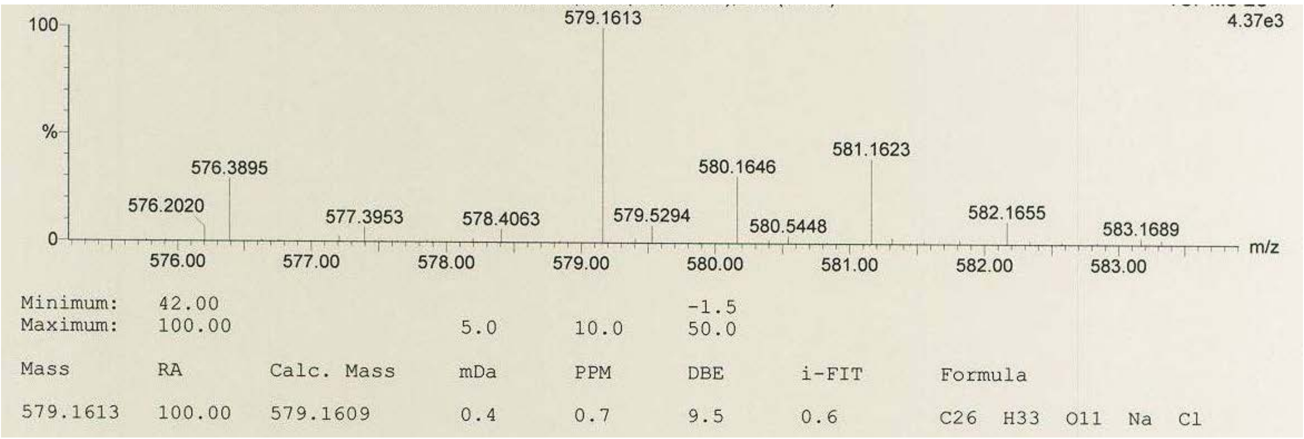

Figure S41. ^{1}H NMR spectrum of the new compound **6**.

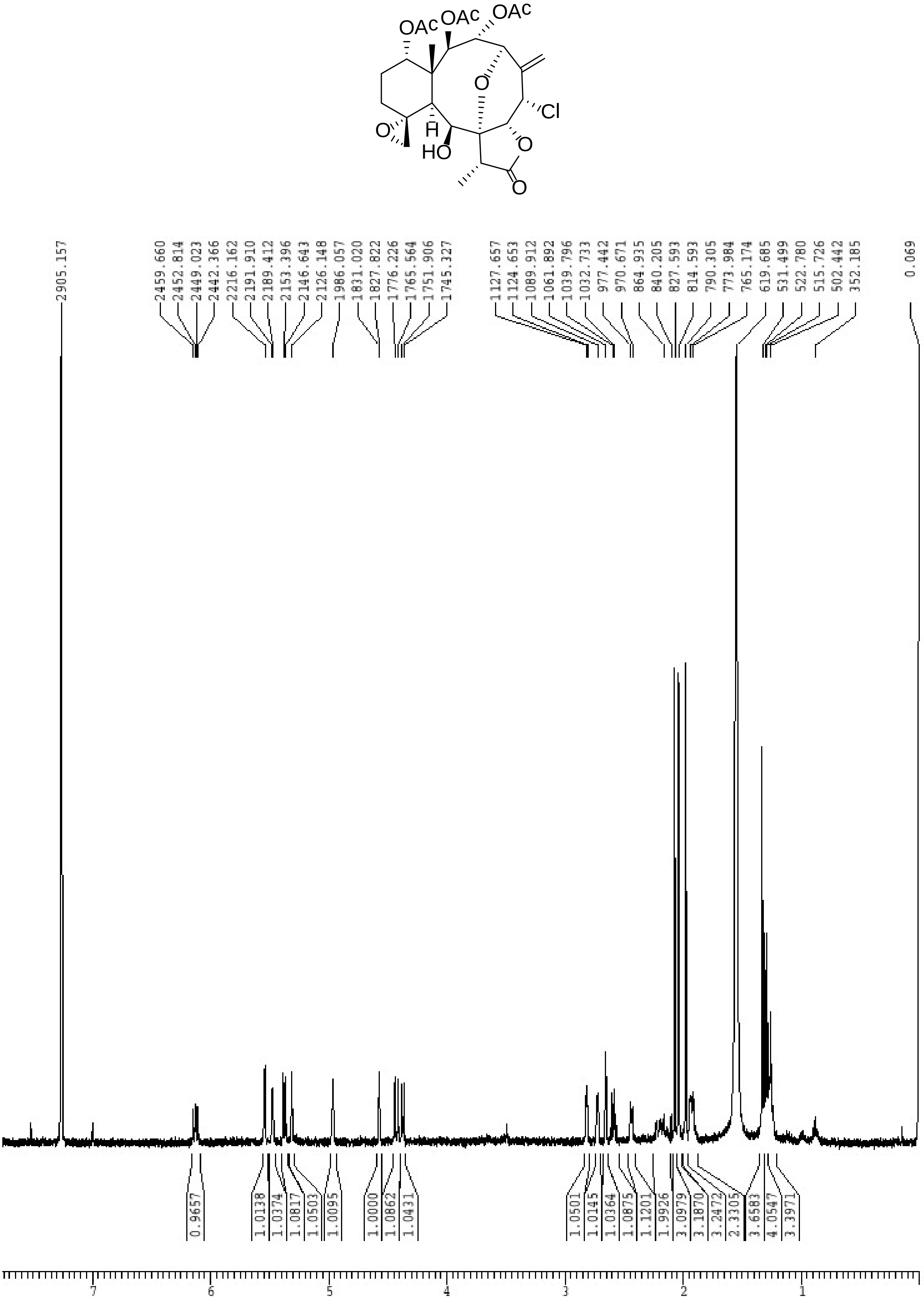

Figure S42. ^{13}C NMR spectrum of the new compound **6**.

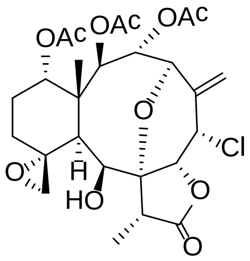

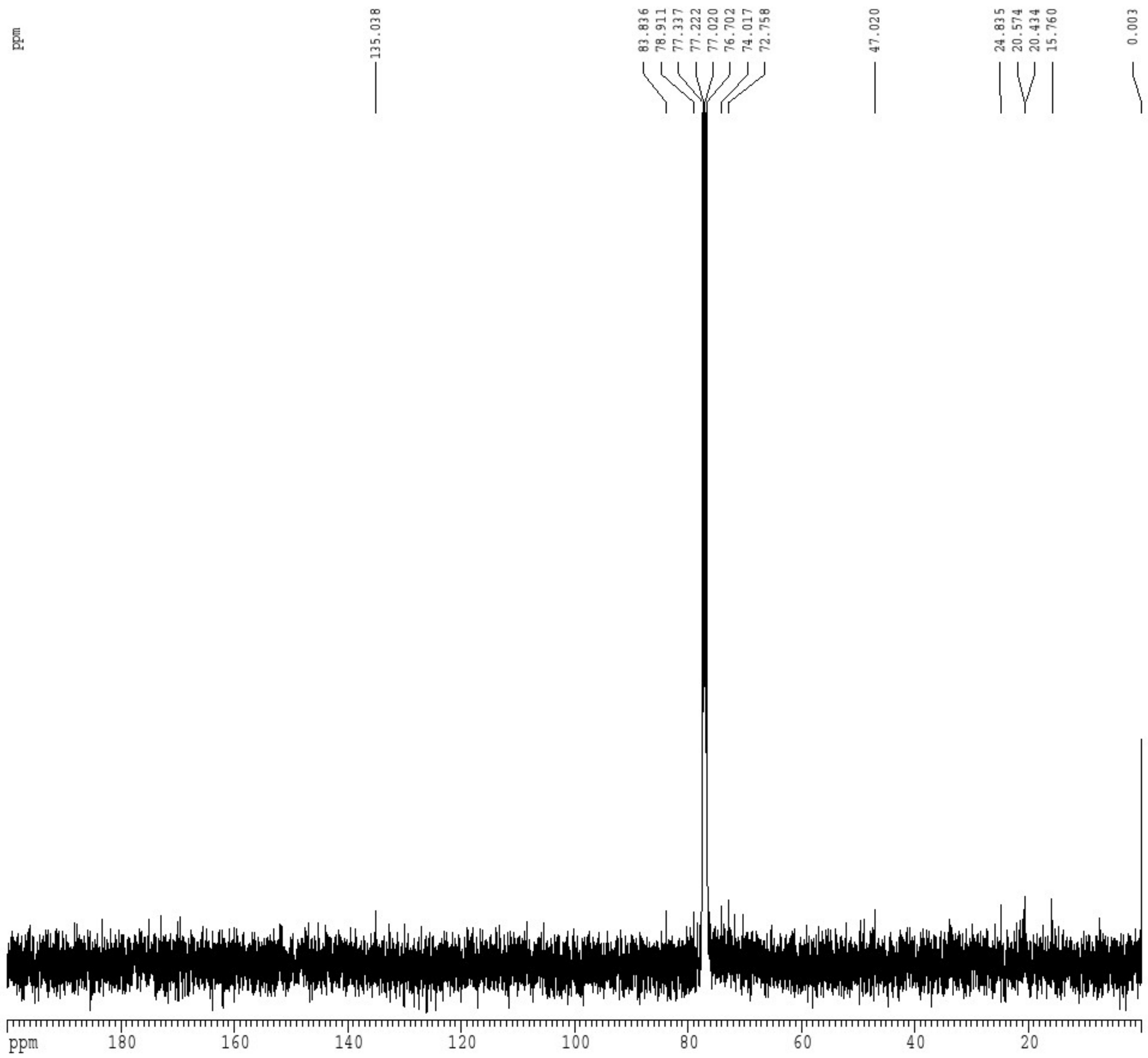

Figure S43. DEPT spectrum of the new compound **6**.

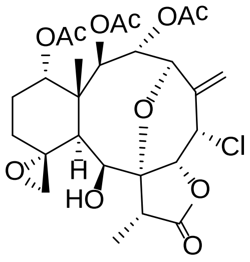

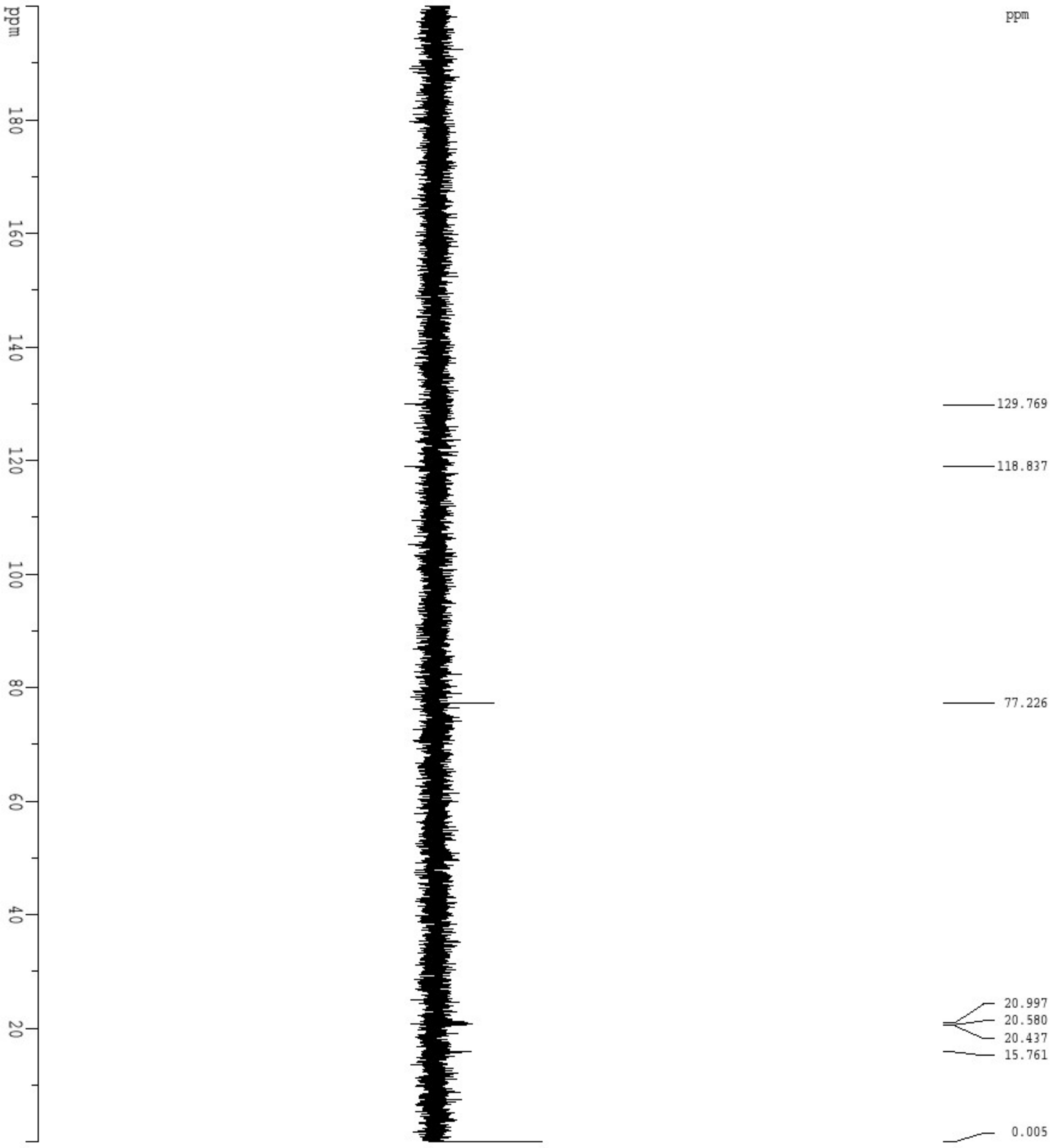

Figure S44. HSQC spectrum of the new compound **6**.

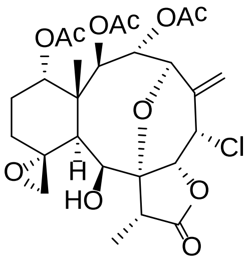

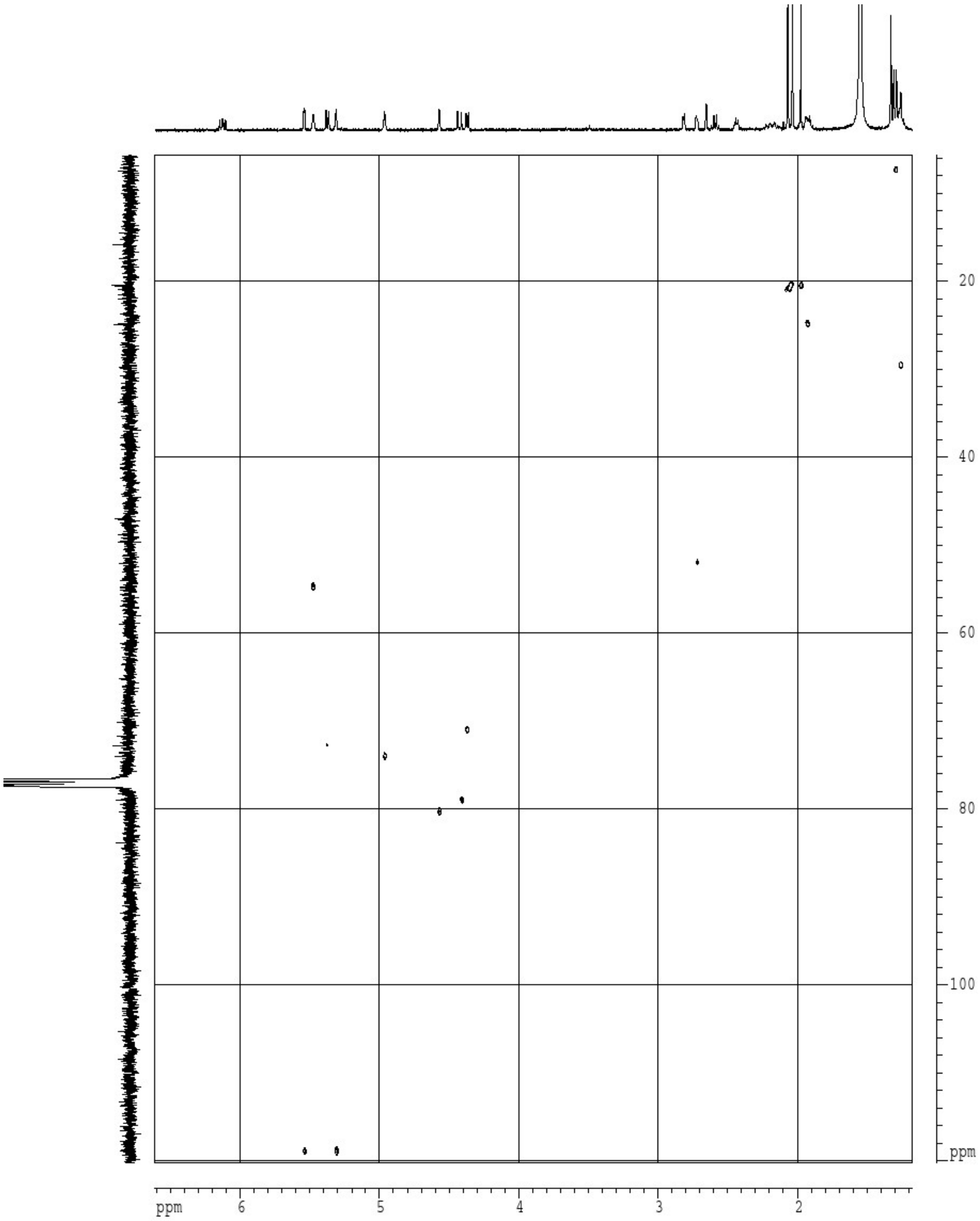

Figure S45. ^{1}H-^{1}H COSY spectrum of the new compound **6**.

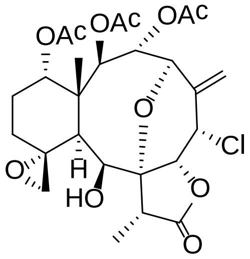

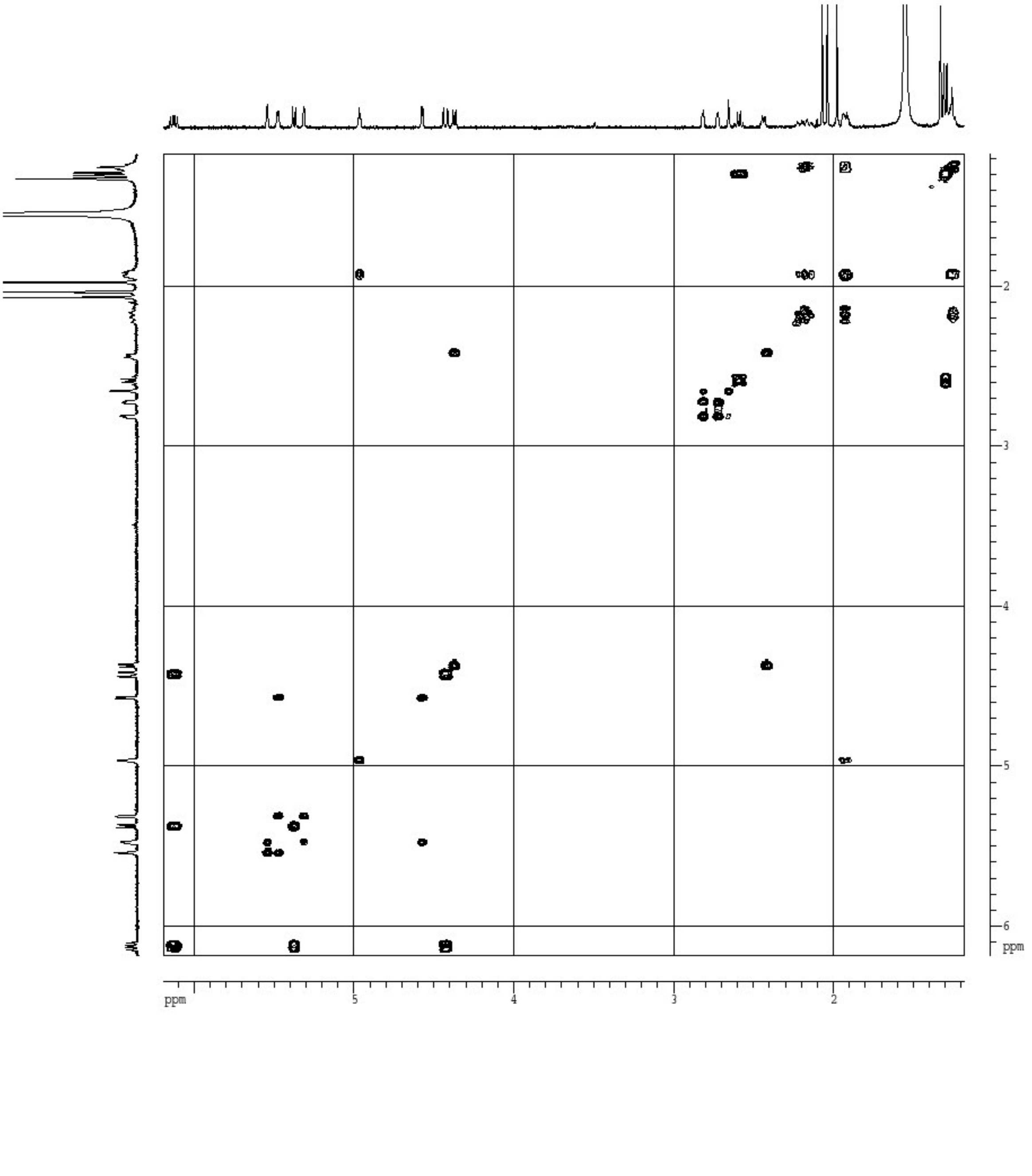

Figure S46. HMBC spectrum of the new compound **6**.

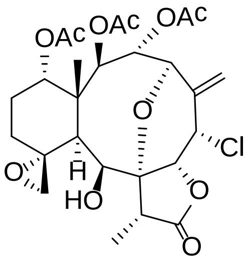

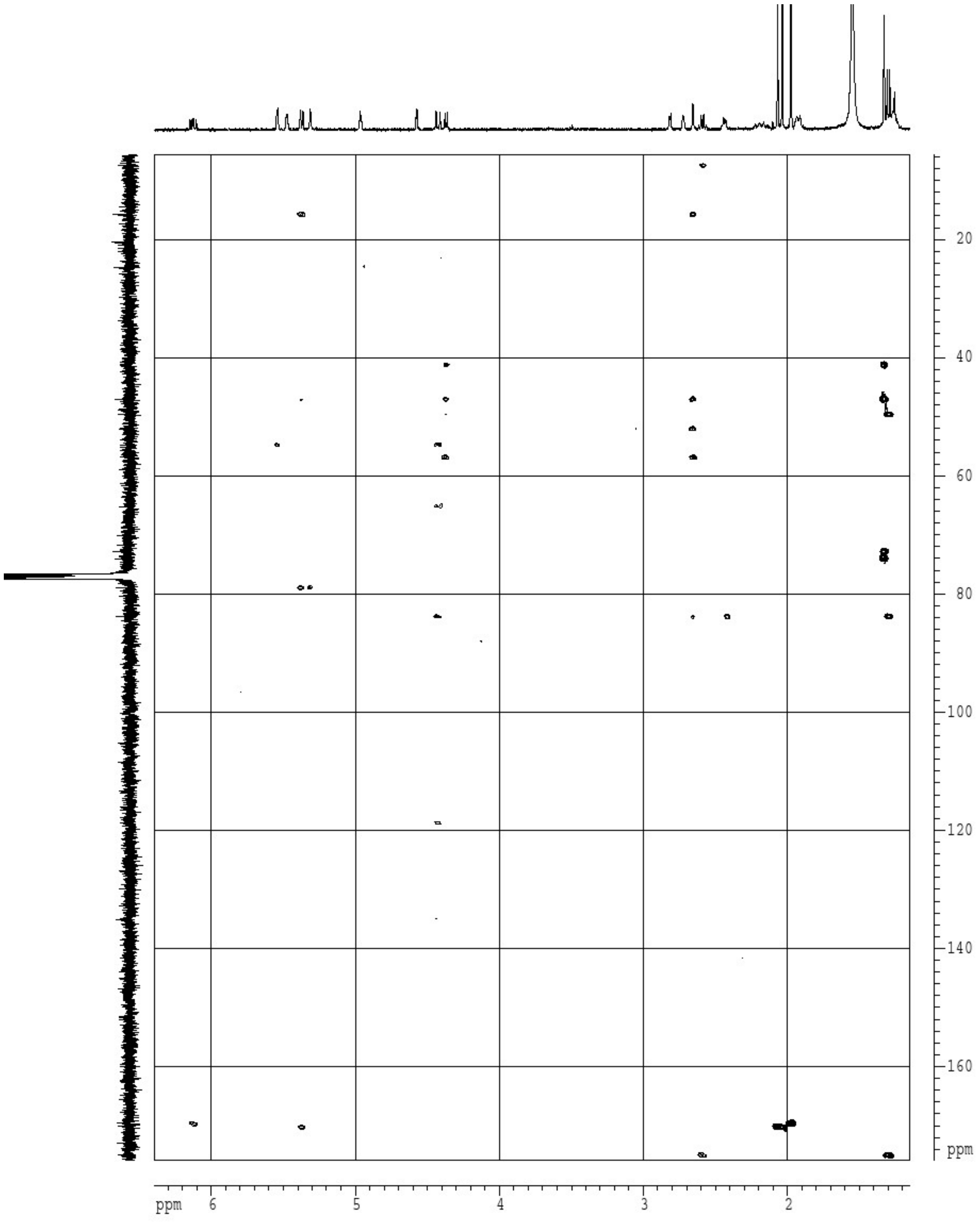

Figure S47. NOESY spectrum of the new compound **6**.

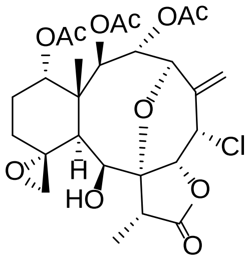

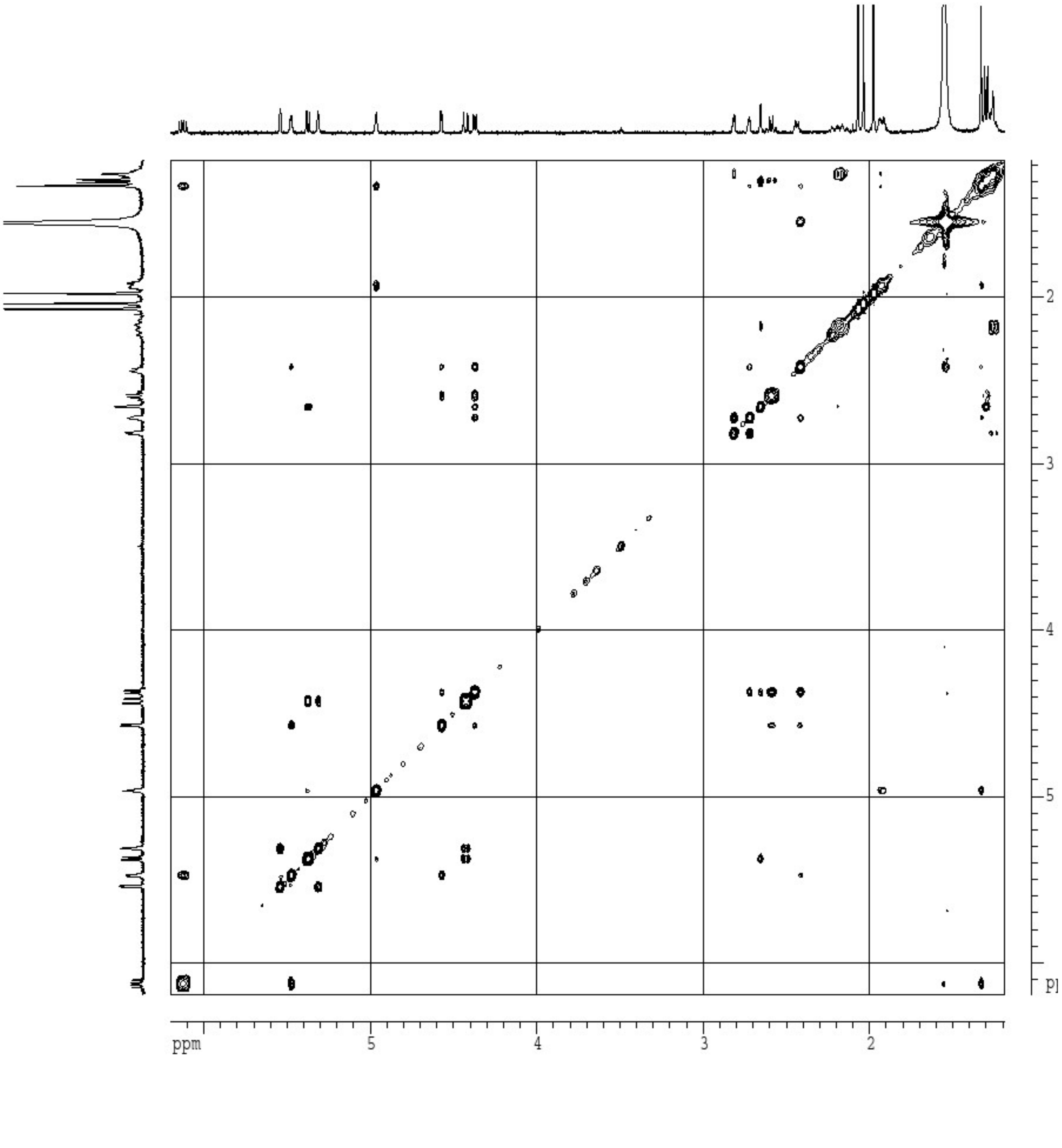

Figure S48. HR-ESIMS spectrum of the new compound **7**.

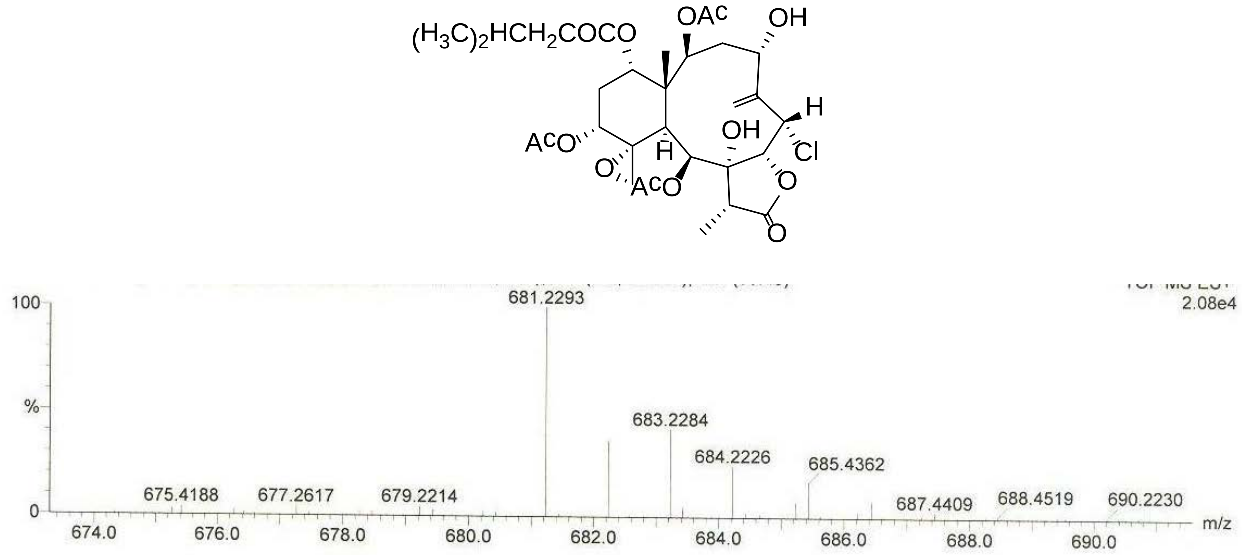

Minimum:	42.00				-1.5		
Maximum:	100.00		5.0	10.0	50.0		
Mass	RA	Calc. Mass	mDa	PPM	DBE	i-FIT	Formula
681.2293	100.00	681.2290	0.3	0.4	9.5	2.1	C31 H43 O13 Na Cl

Figure S49. ^{1}H NMR spectrum of the new compound **7**.

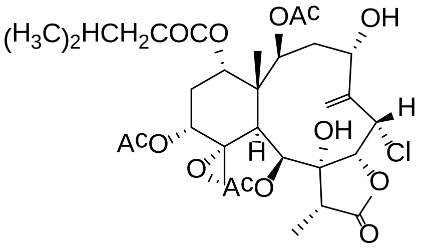

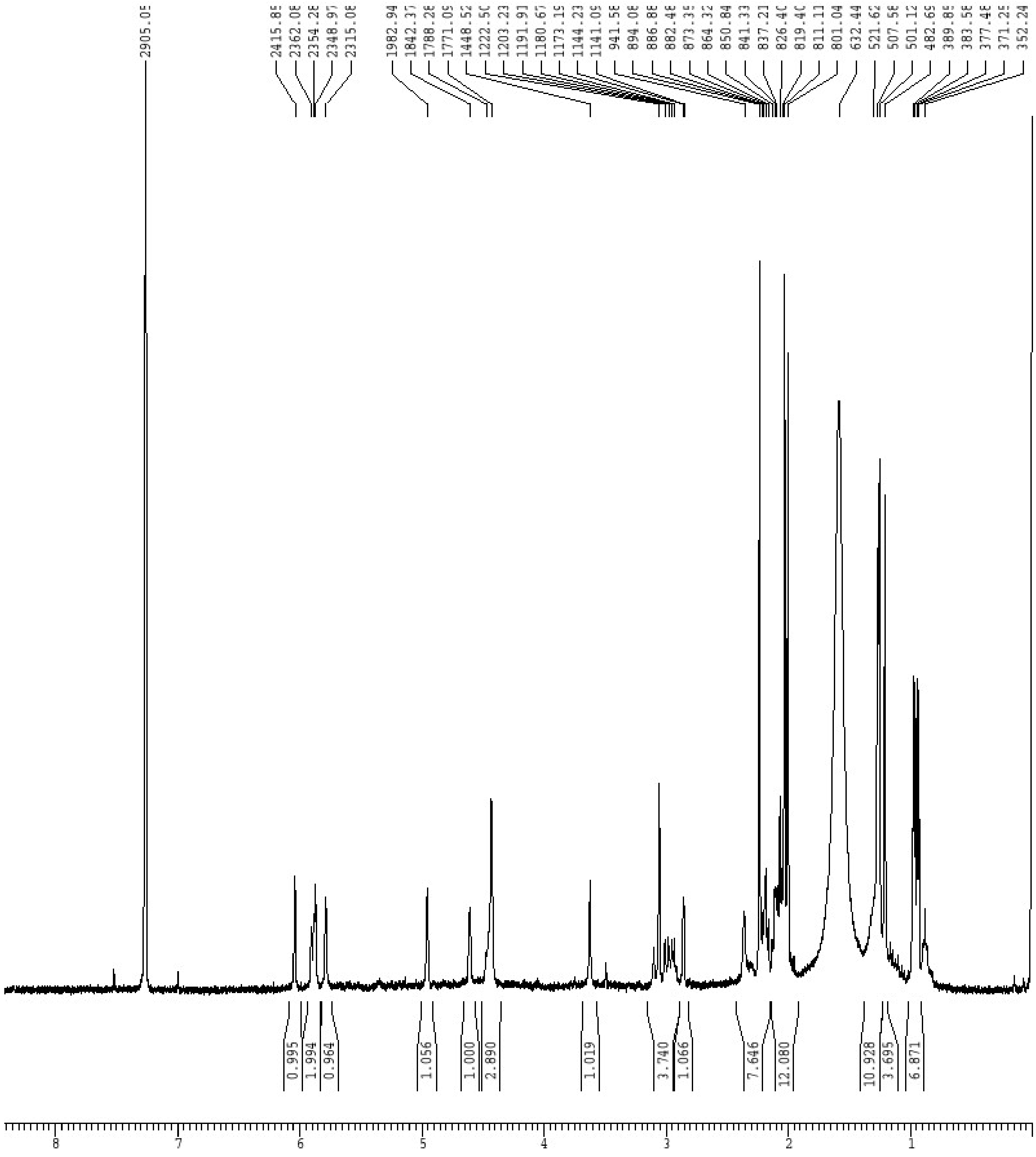

Figure S50. ^{13}C NMR spectrum of the new compound **7**.

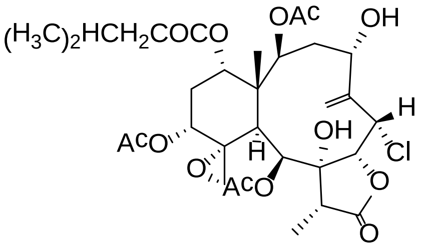

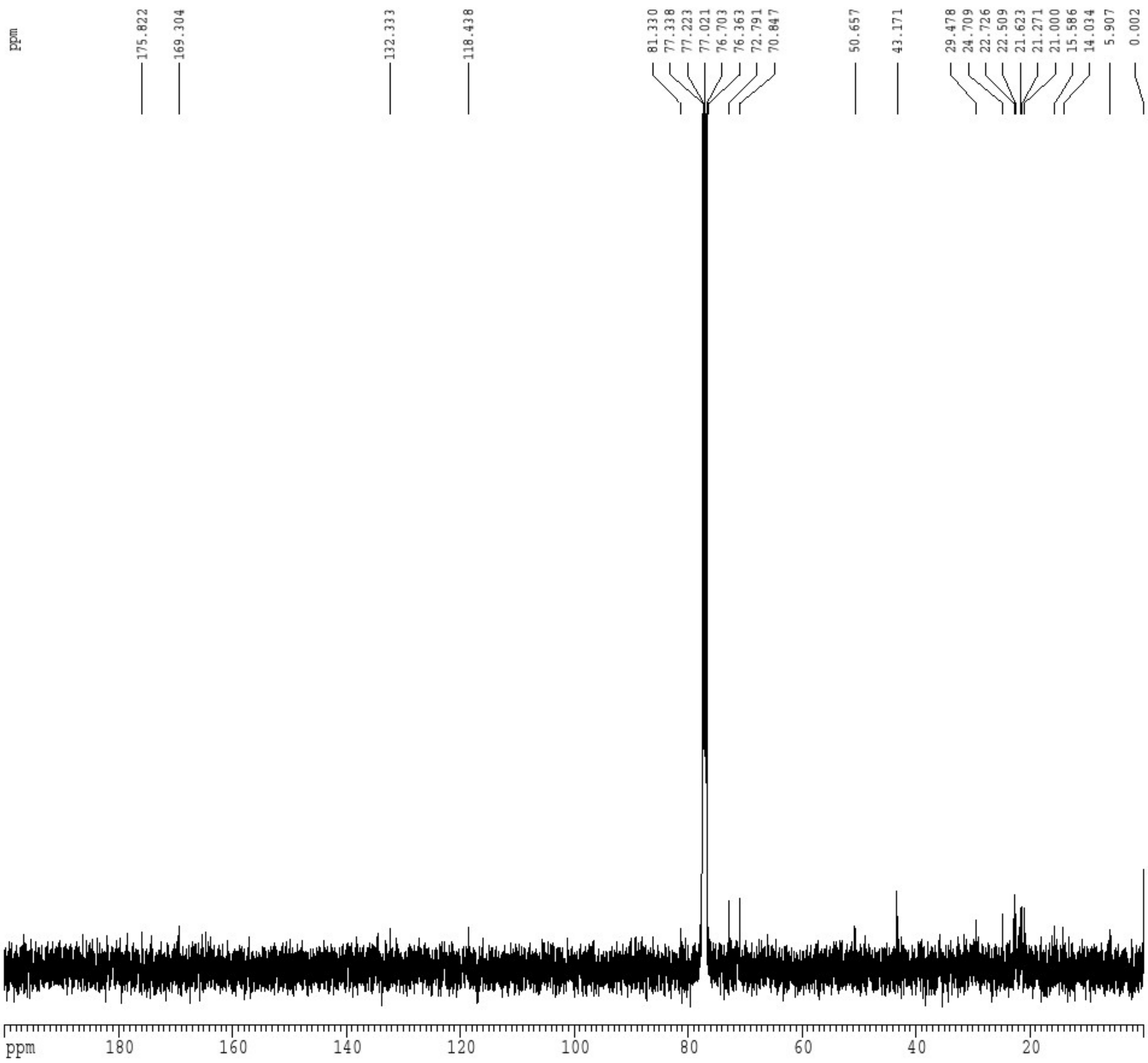

Figure S51. DEPT spectrum of the new compound **7**.

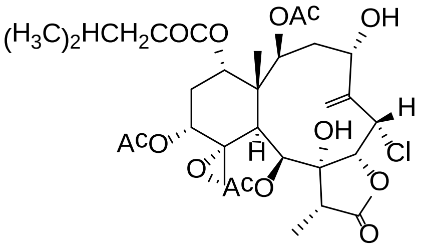

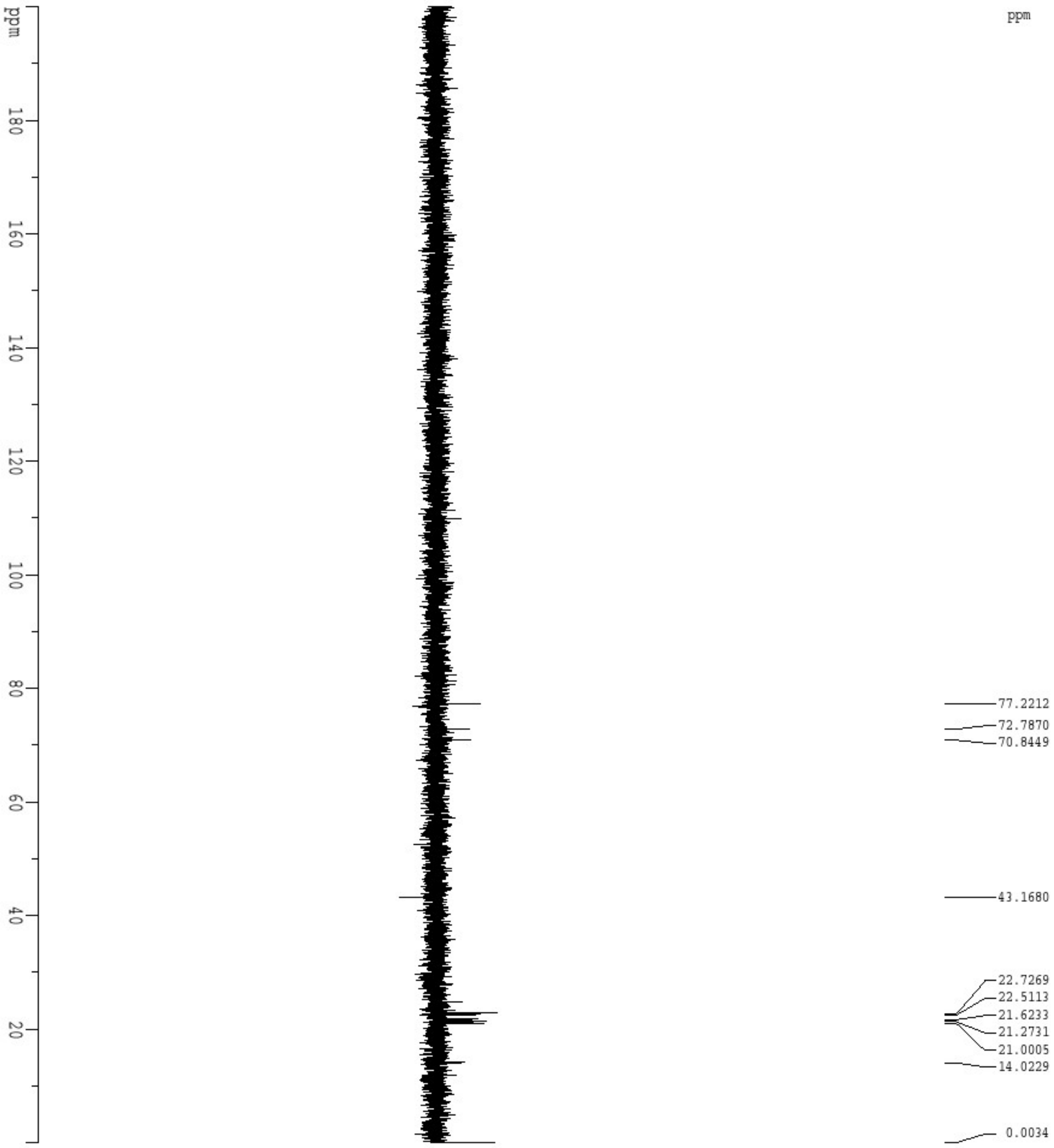

Figure S52. HSQC spectrum of the new compound **7**.

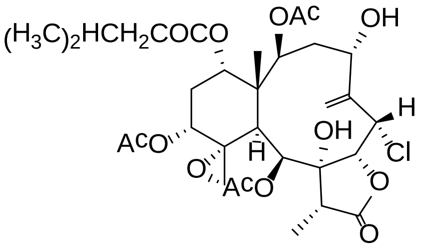

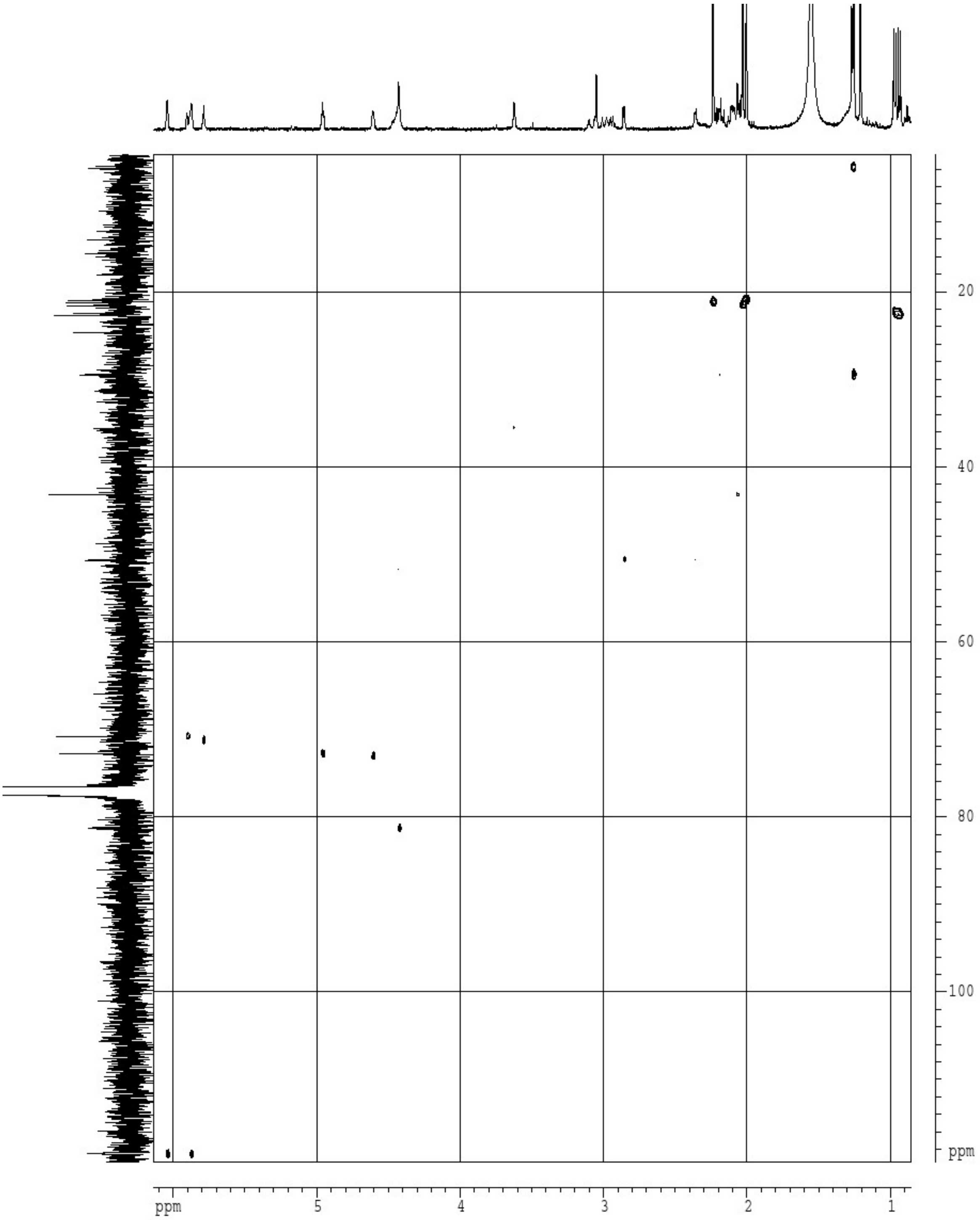

Figure S53. ^{1}H-^{1}H COSY spectrum of the new compound **7**.

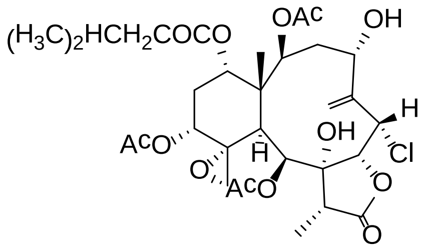

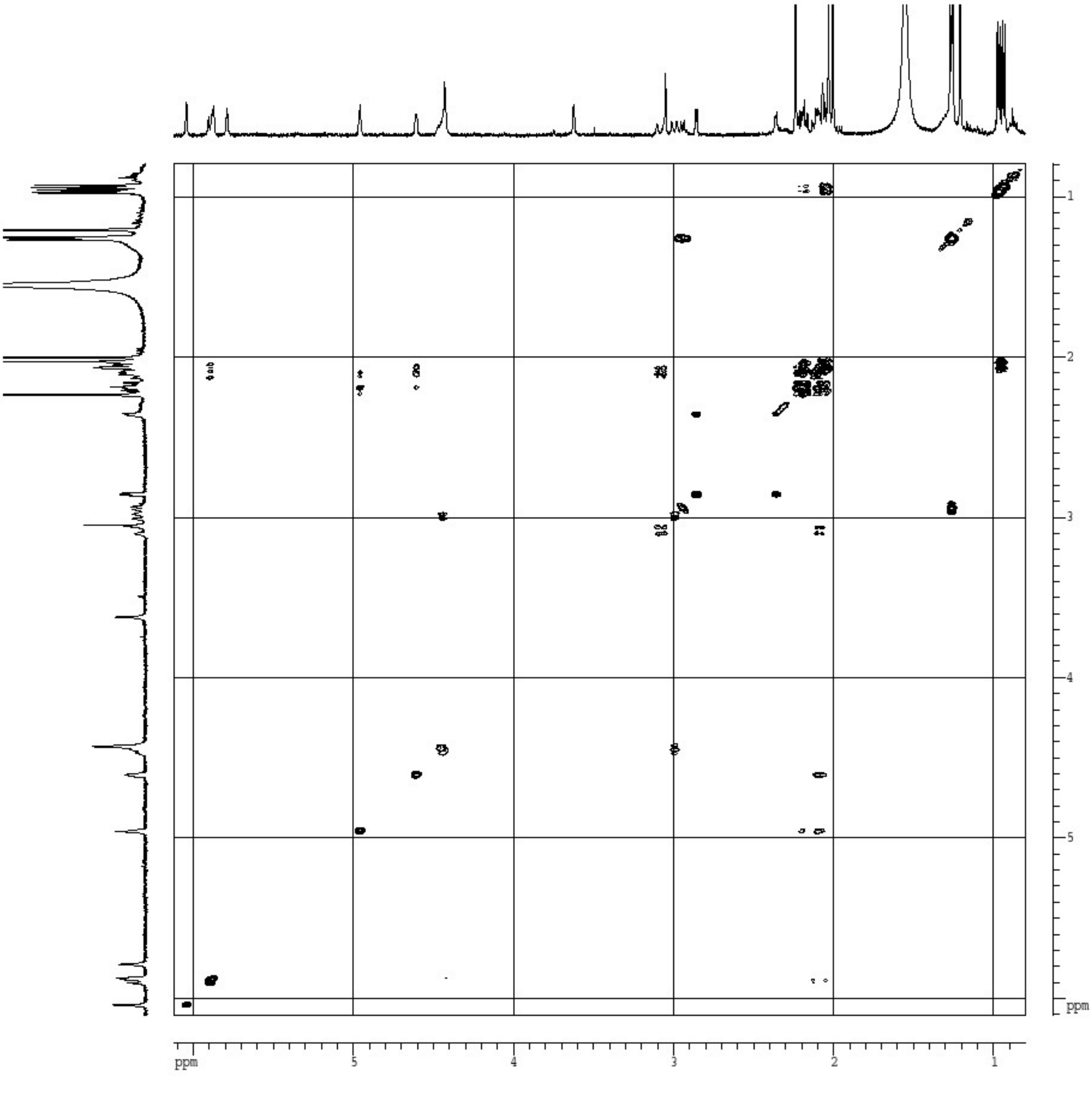

Figure S54. HMBC spectrum of the new compound **7**.

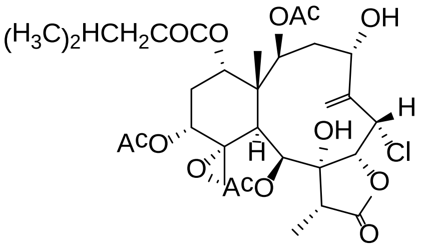

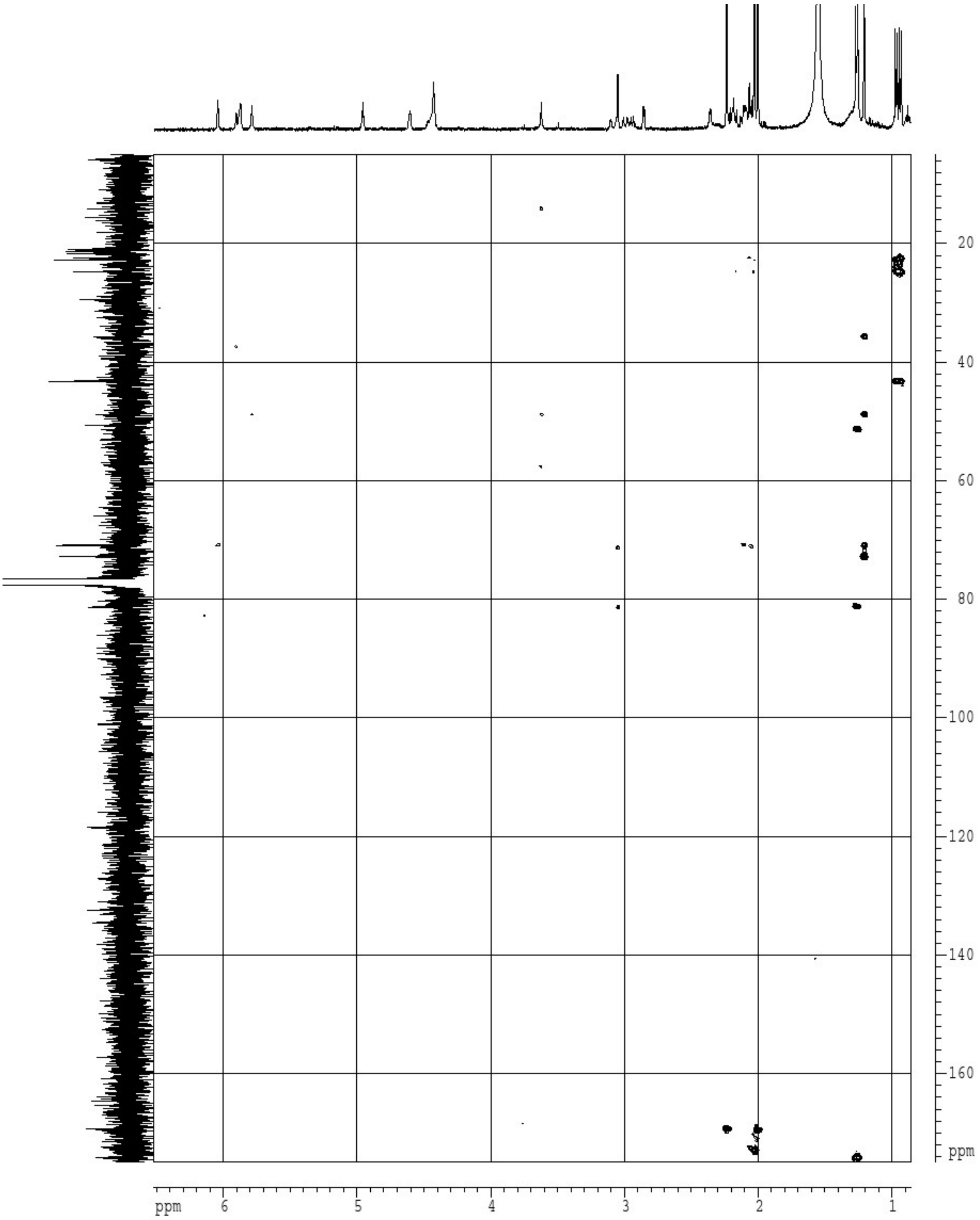

Figure S55. NOESY spectrum of the new compound **7**.

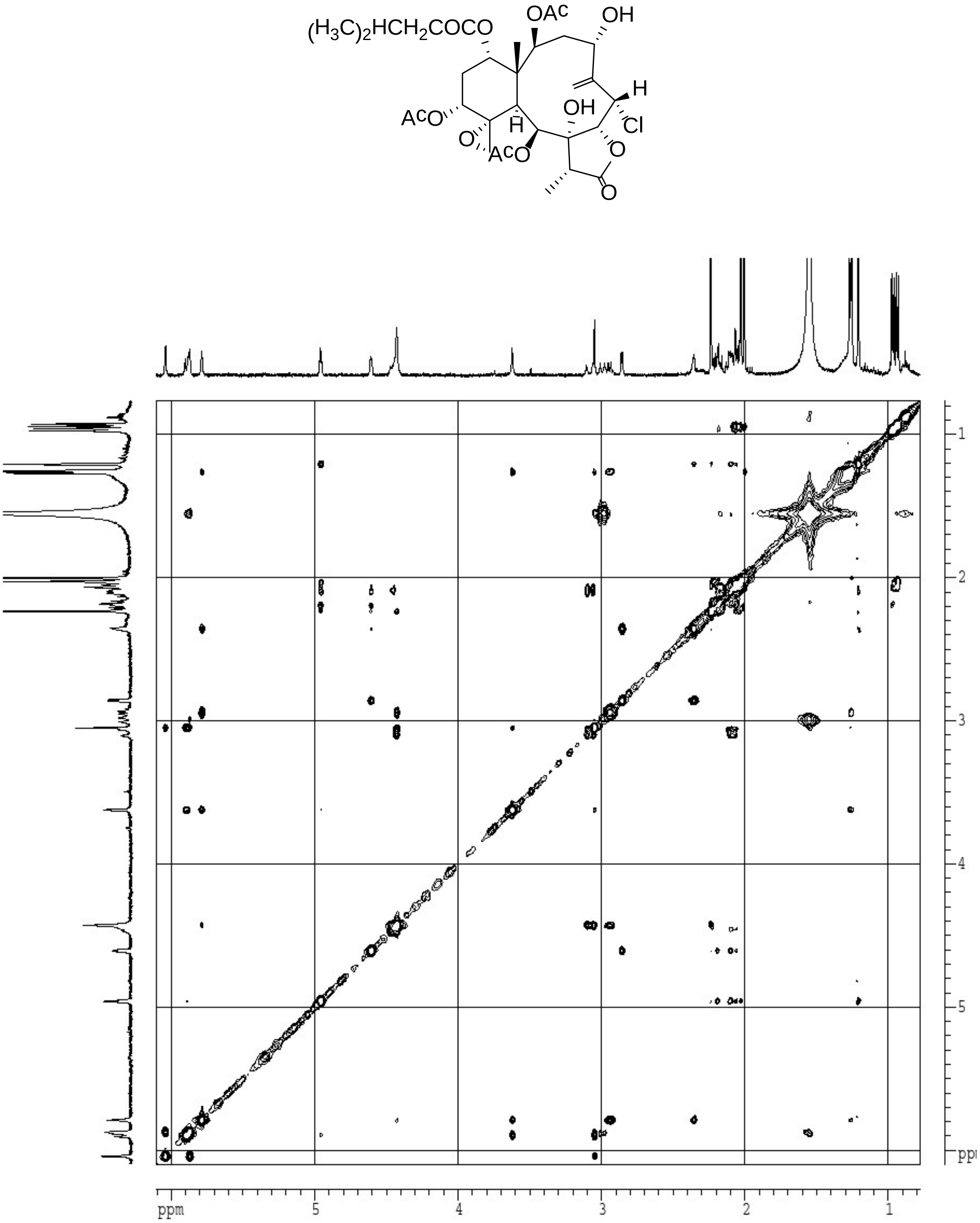

© 2014 by the authors; licensee MDPI, Basel, Switzerland. This article is an open access article distributed under the terms and conditions of the Creative Commons Attribution license (http://creativecommons.org/licenses/by/4.0/).
